# Chromosomal copy number variation reveals differential levels of genomic plasticity in distinct *Trypanosoma cruzi* strains

**DOI:** 10.1186/s12864-015-1680-4

**Published:** 2015-07-04

**Authors:** João Luís Reis-Cunha, Gabriela F. Rodrigues-Luiz, Hugo O. Valdivia, Rodrigo P. Baptista, Tiago A. O. Mendes, Guilherme Loss de Morais, Rafael Guedes, Andrea M. Macedo, Caryn Bern, Robert H. Gilman, Carlos Talavera Lopez, Björn Andersson, Ana Tereza Vasconcelos, Daniella C. Bartholomeu

**Affiliations:** Laboratório de Imunologia e Genômica de Parasitos, Departamento deParasitologia, Universidade Federal de Minas Gerais, Belo Horizonte, Brazil; Laboratório Nacional de Computação Científica, Petrópolis, Rio de Janeiro Brazil; Departamento de Bioquímica e Imunologia, Universidade Federal de Minas Gerais, Belo Horizonte, Brazil; University of California San Francisco, San Francisco, CA USA; Universidad Cayetano Heredia, Lima, MD Peru; Johns Hopkins University, Baltimore, MD USA; Department of Cell and Molecular Biology, Science for Life Laboratory, Karolinska Institutet, Stockholm, Sweden

**Keywords:** Chromosome copy number variation, *Trypanosoma cruzi*, Genomic plasticity

## Abstract

**Background:**

*Trypanosoma cruzi*, the etiologic agent of Chagas disease, is currently divided into six discrete typing units (DTUs), named TcI–TcVI. CL Brener, the reference strain of the *T. cruzi* genome project, is a hybrid with a genome assembled into 41 putative chromosomes. Gene copy number variation (CNV) is well documented as an important mechanism to enhance gene expression and variability in *T. cruzi*. Chromosomal CNV (CCNV) is another level of gene CNV in which whole blocks of genes are expanded simultaneously. Although the *T. cruzi* karyotype is not well defined, several studies have demonstrated a significant variation in the size and content of chromosomes between different *T. cruzi* strains. Despite these studies, the extent of diversity in CCNV among *T. cruzi* strains based on a read depth coverage analysis has not been determined.

**Results:**

We identify the CCNV in *T. cruzi* strains from the TcI, TcII and TcIII DTUs, by analyzing the depth coverage of short reads from these strains using the 41 CL Brener chromosomes as reference. This study led to the identification of a broader extent of CCNV in *T. cruzi* than was previously speculated. The TcI DTU strains have very few aneuploidies, while the strains from TcII and TcIII DTUs present a high degree of chromosomal expansions. Chromosome 31, which is the only chromosome that is supernumerary in all six *T. cruzi* samples evaluated in this study, is enriched with genes related to glycosylation pathways, highlighting the importance of glycosylation to parasite survival.

**Conclusions:**

Increased gene copy number due to chromosome amplification may contribute to alterations in gene expression, which represents a strategy that may be crucial for parasites that mainly depend on post-transcriptional mechanisms to control gene expression.

**Electronic supplementary material:**

The online version of this article (doi:10.1186/s12864-015-1680-4) contains supplementary material, which is available to authorized users.

## Background

American trypanosomiasis is a neglected tropical disease, caused by the protozoan *Trypanosoma cruzi*, a highly polymorphic parasite that belongs to the order Kinetoplastida and family Trypanosomatidae. The distribution of this disease ranges from southern Argentina to the southern United States of America, where it affects eight million people and accounts for 662,000 disability-adjusted life years [1–4].

The *T. cruzi* taxa is currently subdivided into six discrete typing units (DTUs), named TcI – TcVI, due to its high genotypic and phenotypic heterogeneity. The major *T. cruzi* DTUs involved in the domestic cycle of Chagas disease are TcI, TcII, TcV and TcVI [5–11]. The distinct DTUs are differently distributed in the Americas, with TcI prevalent in Central America and in the northern region of South America, while TcII, TcV and TcVI are more common in the Southern cone of South America [10].

*T. cruzi* replication is usually clonal [12, 13], but there is evidence of natural hybridization and genetic exchange between the strains [14–19]. The hybrid nature of *T. cruzi* DTU TcVI was confirmed during the whole-genome sequencing of the TcVI CL Brener clone [20]. Post-assembly comparisons of CL Brener contigs with reads from the *T. cruzi* Esmeraldo TcII strain allowed the differentiation of the two CL Brener haplotypes, named Esmeraldo-like, derived from a TcII ancestor, and non-Esmeraldo-like, derived from a TcIII ancestor [20].

Apparently, TcI has the smallest genome of all the *T. cruzi* DTUs [6, 21, 22], and it seems to have less intragenomic heterogeneity than TcII and TcVI [23]. However, sequences from different TcI strains may present more sequence variability between each other than the variability within the TcII and TcVI strains [8, 23].

Copy number variation (CNV)—the gain or loss of genomic material—may have a phenotypic impact by altering the fitness of an organism. CNV creates paralog genes that may evolve differently than the progenitor gene or that may alter the expression level of a gene or genomic region [24, 25]. In CL Brener, at least 50 % of the genome consists of repetitive sequences, represented primarily by large multigene families that encode surface proteins, retrotransposons, and telomeric and satellite repeats [20, 26]. The CL Brener genome contains approximately 1000 paralogous clusters with more than two genes, encompassing over 8000 genes. Several of these clusters are represented by surface protein-encoding genes that account for 18 % of the total of protein-encoding genes of CL Brener [20]. An increased gene copy number due to chromosomal amplification may contribute to alterations in gene expression, providing a strategy for organisms, such as *T. cruzi,* that depend mainly on post-transcriptional mechanisms to control gene expression [27, 28].

The *T. cruzi* karyotype has not been completely elucidated owing to the inability to perform cytogenetic analysis because there is no apparent chromosome condensation during the parasite cell cycle [8, 22, 29]. Using pulse-field gel electrophoresis (PFGE), various studies have shown a significant variation in chromosome size and content between different *T. cruzi* strains and even between clones of the same strain [5, 29–32]. To better characterize the *T. cruzi* karyotype, the genome sequence of the CL Brener strain was recently assembled into 41 putative chromosomes based on scaffold information, BAC-end sequences, and synteny maps with *Trypanosoma brucei* and *Leishmania major* [8, 33].

Read depth coverage (RDC) analysis allows the identification of extensive variations in the copy number of chromosomes among different species of *Leishmania* [34]. However, the chromosomal copy number variation (CCNV) among *T. cruzi* strains as determined by read depth coverage analysis has not yet been reported. In the present work, we sought to identify the CCNV in *T. cruzi* strains belonging to different DTUs, based on read depth coverage of the 41 CL Brener chromosomes. Identifying the CCNV will lead to explanations of some of the genome structural peculiarities of these DTUs. This analysis also led to the identification of a broader extent of CCNV in *T. cruzi* than previously speculated, especially in strains from the TcII and TcIII DTUs.

## Results

### Competitive mapping and SNP content

**Fig. 1:**
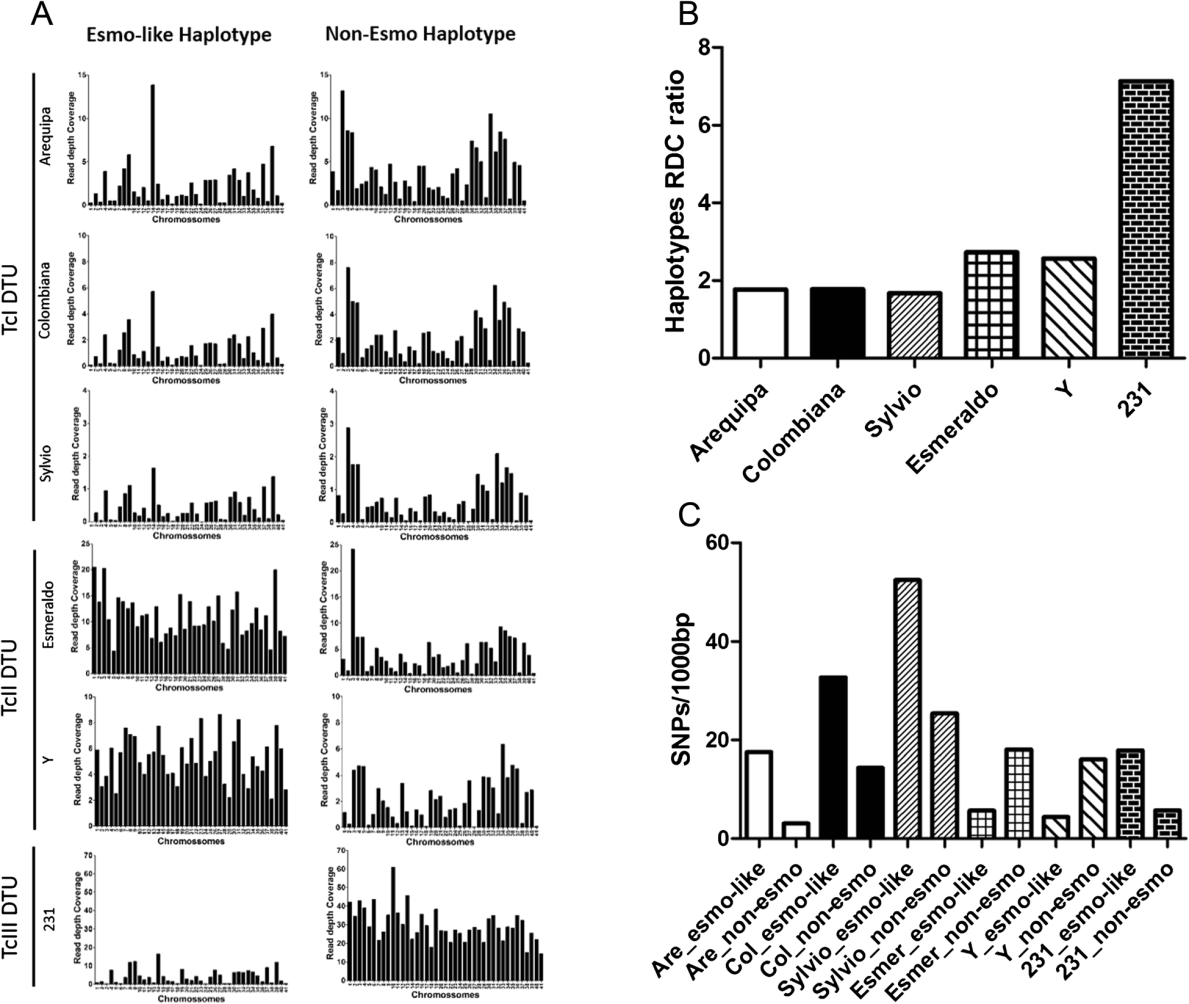
Competitive mapping and SNP content of the *T. cruzi* strains from different DTUs. In competitive mapping, whole-genome shotgun reads from the *T. cruzi* Arequipa, Colombiana, Sylvio, Esmeraldo, Y and 231 strains were simultaneously mapped to the 41 chromosomal sequences from Esmeraldo-like (Esmo-like) and non-Esmeraldo-like (Non-Esmo) CL Brener haplotypes, and their mean read depth coverage (RDC) was estimated. Each black bar corresponds to the mean RDC of each of the 41 CL Brener chromosomes (**a**). For each *T. cruzi* strain, the mean RDC of the CL Brener haplotype with the highest RDC in the competitive mapping (non-Esmeraldo for Arequipa, Sylvio and 231; Esmeraldo-like for Esmeraldo and Y) was divided by the mean RDC of the CL Brener haplotype with the lower RDC (Esmeraldo-like for Arequipa, Sylvio and 231; non-Esmeraldo for Esmeraldo and Y) (**b**). The SNP density of each *T. cruzi* strain and the 1563 Esmeraldo-like/non-Esmeraldo-like single-copy genes are shown (**c**)

To select the CL Brener haplotype most suitable as a reference in the mapping of the reads from the distinct *T. cruzi* strains, their quality-filtered reads were mapped simultaneously to the 41 chromosomal sequences from the CL Brener Esmeraldo-like and non-Esmeraldo-like haplotypes (Fig. 1a). As expected, the reads from the TcII strains (Esmeraldo and Y) mapped preferentially with chromosomes from the Esmeraldo-like haplotype, and the reads from the TcIII strain (231) mapped preferentially with chromosomes from the non-Esmeraldo-like haplotype. The reads from the strains from TcI DTU (Arequipa, Colombiana and Sylvio) mapped slightly better to the non-Esmeraldo-like than to the Esmeraldo-like CL Brener haplotype (Fig. 1b).

To further confirm the selection of the haplotype to be used as a reference for read mapping, the filtered reads from each strain were mapped separately to both CL Brener haplotypes, and then the number of SNPs/1000 bp in the CL Brener single-copy genes for each combination of strain-haplotype was estimated (Fig. 1c). The numbers of SNPs/1000 bp between each *T. cruzi* strain and the CL Brener Esmeraldo-like and non-Esmeraldo-like haplotypes were, respectively, 17.50 and 3.10 for Arequipa; 32.74 and 14.37 for Colombiana; 52.41 and 25.36 for Sylvio; 5.66 and 18.07 for Esmeraldo; 4.39 and 16.08 for Y; and 17.89 and 5.72 for 231.

Based on these results, the CL Brener non-Esmeraldo-like haplotype was selected as a reference for the mapping of Arequipa, Colombiana, Sylvio and 231 reads, and the CL Brener Esmeraldo-like haplotype was selected as a reference for the mapping of Esmeraldo and Y strain reads.

### Methodology to estimate chromosome copy number in *T. cruzi* strains

**Fig. 2:**
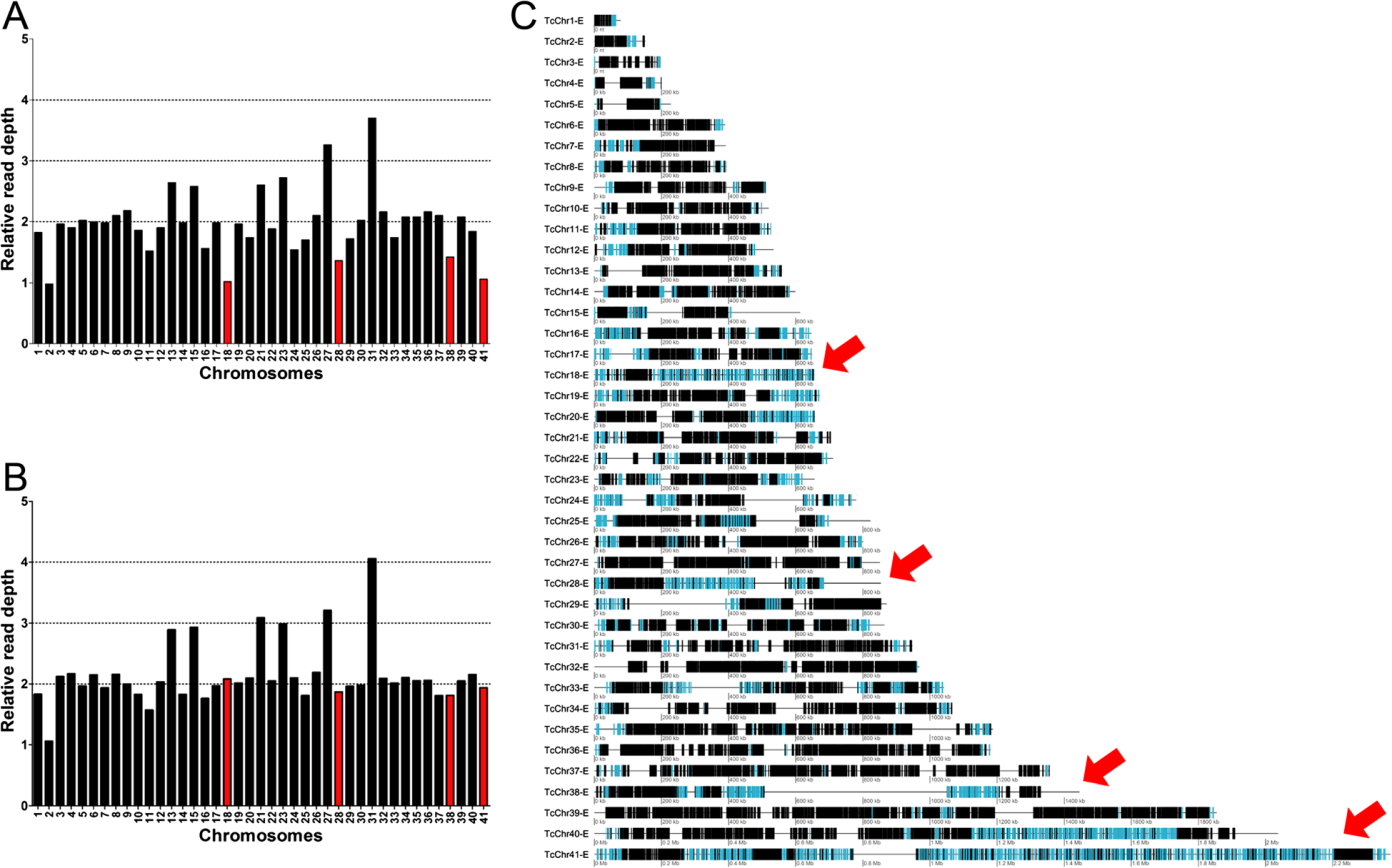
Methodologies to determinate the chromosomal copy number in *T. cruzi* strains. To estimate the chromosome ploidy of *T. cruzi* strains, two approaches were evaluated by mapping reads from the Y strain on the 41 CL Brener chromosomes. **a** The Whole Chromosome Ploidy Estimations (WCPE) predicts the ploidy based on the mean coverage of the whole-chromosome sequence. **b** The single-copy genes ploidy estimations (SCoPE) predict the chromosomal ploidy based only on the coverage of the 1563 Esmo-like/Non-Esmo single-copy genes present in each chromosome. The read depth was scaled to give a value of 2 for disomic chromosomes. **c** The 41 *T. cruzi* chromosome gene distribution is shown. Multicopy gene families are represented as blue boxes and the hypothetical and housekeeping genes as black boxes. The red arrows highlight the chromosomes with a higher content of multigene families or with the largest proportion of gap regions. Chromosomes with a higher density of multigene families or that present large gap regions tend to have a biased lower predicted ploidy in the WCPE when compared to the SCoPE method. Similar results were obtained for all the other strains evaluated

Approximately 50 % of the *T. cruzi* genome corresponds to repetitive sequences, including multigene families that account for much of the differences in the gene content of the assembled genomes of CL Brener (TcVI) and Sylvio (TcI) [20, 21]. The chromosomal sequence representation of the CL Brener non-Esmeraldo-like and Esmeraldo-like haplotypes [33] also contains large internal gap regions that may reduce the accuracy of the predicted ploidy based on RDC. To reveal the best methodology to determine the *T. cruzi* chromosome ploidy based on RDC, two approaches were evaluated. In the Whole Chromosome Ploidy Estimations (WCPE) approach, the chromosomal ploidy prediction for each chromosome was estimated based on the ratio between the mean RDC of each chromosome position and the genome coverage (Fig. 2a). This approach accounts for the coverage of all positions in a given chromosome to estimate its copy number, including repetitive and gap regions. In the single-copy genes ploidy estimations (SCoPE) approach, estimations of the chromosomal ploidy for each chromosome were based on the ratio between the mean coverage of all single-copy genes in a given chromosome and the genome coverage (Fig. 2b). This approach infers the copy number for each chromosome based only on the RDC of the 1563 1:1 orthologs between CL Brener Esmeraldo-like and non-Esmeraldo-like haplotypes, which were assumed to be single-copy genes in the haploid CL Brener genome content (Additional file 1: Table S1). As shown in Fig. 2, chromosomes that are rich in multigene families, repetitive sequences and gaps, such as chromosomes 18, 28, 38 and 41 (Fig. 2c), tend to have a lower predicted ploidy as determined using WCPE methodology when compared to the SCoPE approach using the Y strain reads. Similar results were obtained by mapping reads from the other *T. cruzi* strains on the 41 CL Brener chromosomes (data not shown). As the SCoPE approach is less prone to bias toward chromosomal repetitive content, this methodology was chosen to estimate the chromosomal ploidy for each of the strains used in this study.

### Chromosome copy number variation in *T. cruzi* strains

**Fig. 3:**
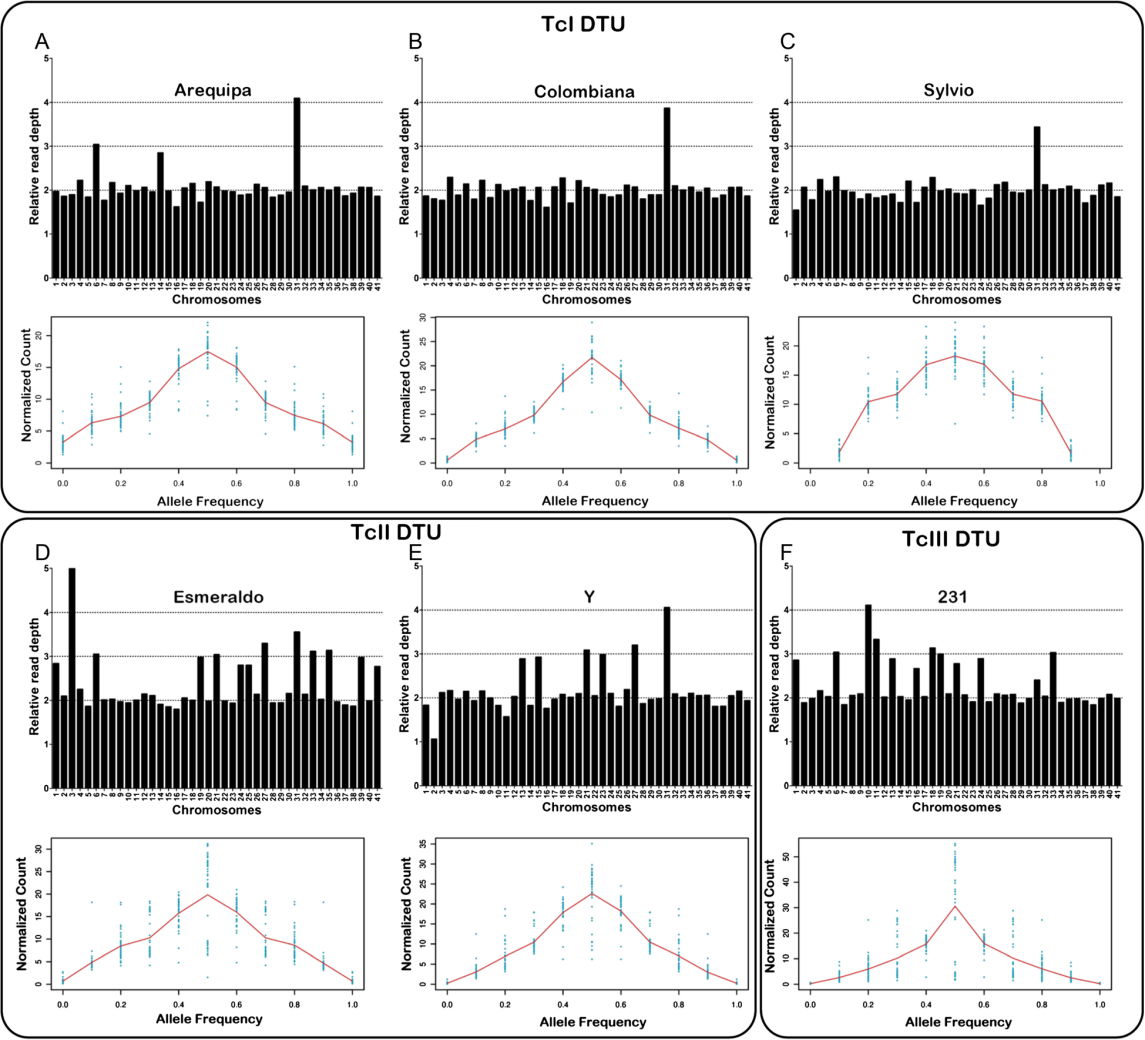
Predicted ploidy of the six *T. cruzi* strains. The predicted ploidy of each chromosome from the *T. cruzi* strains Arequipa (**a**), Colombiana (**b**) and Sylvio (**c**) from the TcI DTU; Y (**d**) and Esmeraldo (**e**) from the TcII DTU; and 231 (**f**) from the TcIII DTU, using as a reference the 41 CL Brener chromosome sequences, was estimated with the SCoPE approach. Each black bar corresponds to the ratio between the single-copy gene mean RDC in the chromosome and the single-copy gene mean RDC of the whole genome, representing its predicted chromosome copy number. The overall ploidy of each strain was estimated by the proportion of the alleles in heterozygous SNP positions, where a tendency of 0.4-0.5 represents a diploid chromosome and a proportion of 0.3 and/or 0.7 corresponds to a triploid chromosome (red line)

The SCoPE approach was used to estimate the chromosome ploidy of the *T. cruzi* Arequipa, Colombiana, Sylvio, Esmeraldo, Y and 231 strains. Initially, based on the mean RDC of 1563 CL Brener single-copy genes, the genome coverage was estimated for each strain: 47× for Arequipa, 28× for Colombiana, 9× for Sylvio, 52× for Esmeraldo, 34× for Y and 76× for 231. The normalized read depth coverage and the percentage of length coverage of each single-copy gene in each chromosome are provided in Additional file 2: Table S2. To determine the overall chromosome ploidy of each *T. cruzi* strain, the allele frequencies were estimated for each predicted heterozygous site. To this end, the proportion of each allele in the heterozygous sites divided by the total read depth for the site was determined and rounded to the first decimal place. Based on this estimation, a diploid chromosome usually has a tendency of 0.4-0.5 and a triploid of 0.3 and 0.6. A tetraploid chromosome has a more complex pattern, which can be 0.4-0.5, 0.2 and 0.8 or a combination of both. As the majority of the heterozygous SNPs show a proportion of 0.4-0.5, the overall ploidy of all the strains was assumed to be diploid (Fig. 3).

The chromosome CNV analysis revealed large differences between the *T. cruzi* strains from different DTUs and some differences between members of the same DTU. Apparently, strains from the TcI DTU have a more stable karyotype when compared to strains from the TcII and TcIII DTUs (Fig. 3). Strains from the TcI DTU usually only have an aneuploidy in chromosome 31, with the exception of the Arequipa strain, which also has a trisomy in chromosomes 6 and 14. The strains from the TcII DTU have a more plastic karyotype with several predicted supernumerary chromosomes and a monosomy case. The Esmeraldo strain has chromosome 3 as pentasomic; chromosomes 27, 31, 33 and 35 range from trisomic to tetrasomic; chromosomes 6, 19, 21 and 39 as trisomic; and chromosomes 1, 24, 25 and 41 range from disomic to trisomic. The Y strain, also from TcII DTU, has tetrasomy of chromosome 31; chromosomes 21 and 27 range from trisomic to tetrasomic; trisomy of chromosomes 13, 15, and 23; and monosomy of chromosome 2. The representative of TcIII DTU, 231, has tetrasomy of chromosome 10; chromosomes 11 and 18 range from trisomic to tetrasomic; trisomy of chromosomes 6 and 19; and chromosomes 1, 13, 16, 21, 24 and 31 have a ploidy ranging from disomic to trisomic (Fig. 3).

To further confirm the chromosomal ploidy, the distribution of base frequencies between the heterozygous SNP positions among all the CDSs in the 41 chromosomes of the six *T. cruzi* strains were estimated (Additional file 3: Figure S1). This analysis is in agreement with the CCNV results predicted by the SCoPE methodology. The only exceptions were chromosomes 20 and 23 from Sylvio and chromosome 7 from Esmeraldo, which were predicted as tetraploid by the heterologous SNP proportion and as diploid in the SCoPE analysis, and chromosomes 6 and 14 from Arequipa that were tetraploid by the heterologous SNP proportion and triploid in the SCoPE analysis.

**Fig. 4:**
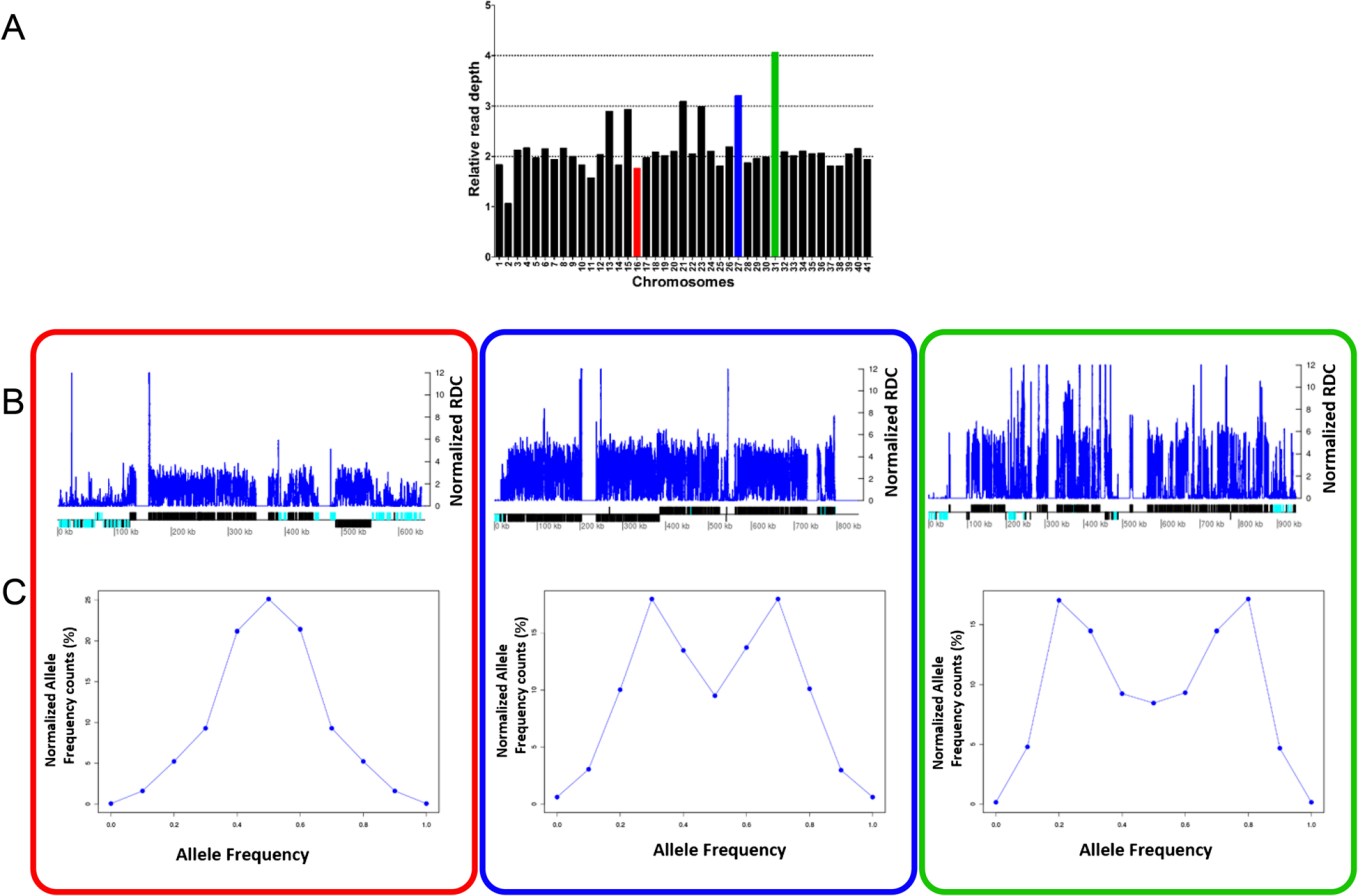
Correspondence between the ploidy predicted by the SCoPE approach and the normalized read depth coverage (RDC) along the entire chromosome sequences. The correspondence between the chromosomal ploidy and the normalized read depth coverage of chromosomes 16 (red box), 27 (blue box) and 31 (green box) of the *T. cruzi* Y strain is shown. **a** Chromosomal ploidy predicted by the SCoPE approach. **b** The blue line corresponds to the normalized RDC of each position, estimated by the ratio between the RDC and the genome coverage. Below, the protein-coding genes are depicted as rectangles drawn as proportional to their length, and their coding strand is indicated by their position above (top strand) or below (bottom strand) the central line. Cyan and black rectangles represent multigene families and hypothetical/housekeeping genes, respectively. Gaps are represented by gene-less regions with no read coverage. Chromosome 16 was predicted as disomic, 27 as trisomic and 31 as tetrasomic. The regions of low coverage correspond to regions rich in multigene families or chromosomal gap regions. **c** The predicted ploidy based on the proportion of the alleles in the heterozygous SNP positions for chromosomes 16, 27 and 31 is shown. The peak of 0.5 classifies chromosome 16 as diploid, the peaks of 0.3 and 0.6 classify chromosome 27 as triploid, and the peaks of 0.2-0.8 and 0.5 classify chromosome 31 as tetraploid

To evaluate if these predicted aneuploidies were produced by the gain or loss of a whole chromosome, or if they result from segmental duplication or loss of partial fragments from these chromosomes, the normalized read depth coverage of each position along each chromosome of the six *T. cruzi* strains was estimated (Additional file 4: Figure S2). Figure 4 represents the read depth coverage along each position of the predicted disomic, trisomic and tetrasomic chromosomes of the Y strain, as well as the base frequency distribution between the heterozygous SNP positions. As expected, with the exception of the regions that are rich in multigene families and gaps, the predicted ploidy along the entire chromosome is in agreement with the predicted ploidy based on the SCoPE and SNP analyses. This finding suggests that these aneuploidies are probably a result of a whole chromosomal duplication/loss.

**Fig. 5:**
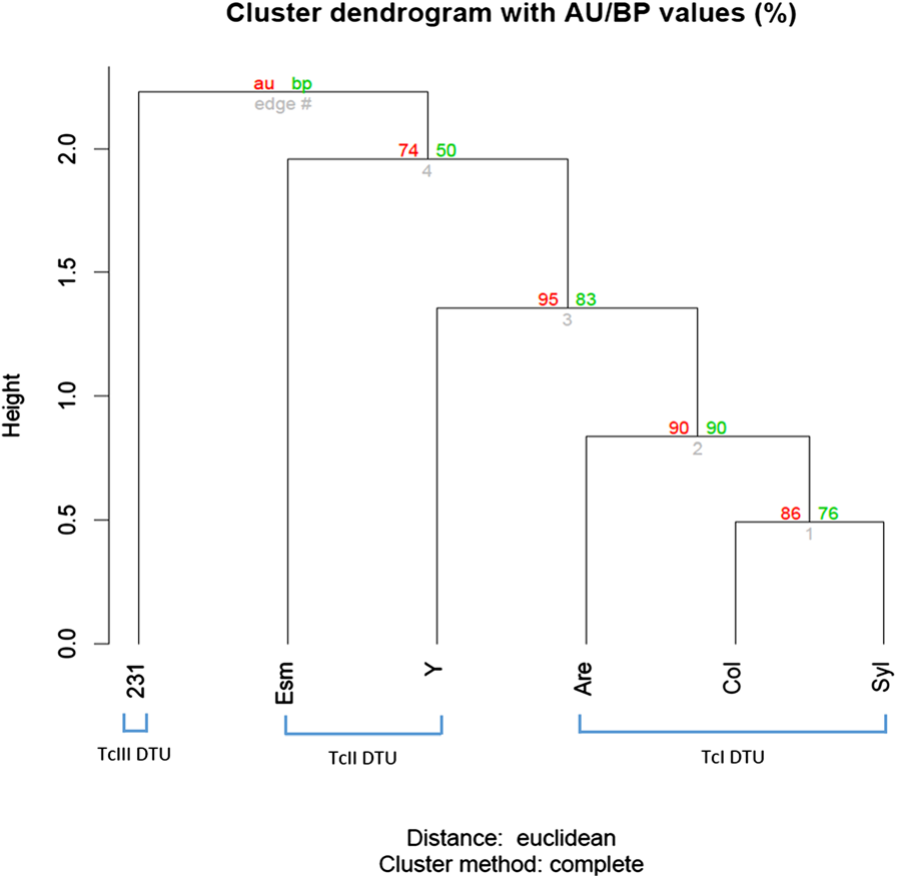
Dendrogram of the hierarchical analysis of the *T. cruzi* strains’ predicted ploidy. The hierarchical clustering analysis based on Euclidian distances of the predicted ploidy of each chromosome from the *T. cruzi* Arequipa, Colombiana, Sylvio, Esmeraldo, Y and 231 strains was performed using the Pvclust package and the R software platform. Two bootstrap resampling methods were used to assess the uncertainty in the hierarchical cluster analysis: the approximately unbiased (au) in red, and the bootstrap probability (bp) in green

To determine whether the ploidy profile of the six *T. cruzi* strains is in agreement with their phylogeny, a hierarchical clustering analysis was performed, using as input the predicted ploidy of each chromosome of each strain based on the SCoPE approach. The clustering analysis was based on their pairwise Euclidean distances using the Complete Linkage method (Fig. 5). In this analysis, all strains belonging to the TcI DTU clustered together, with Colombiana and Sylvio strains being closer to each other than to the Arequipa strain. This is likely due to the presence of exclusive aneuploidies in chromosomes 6 and 14 from Arequipa. Both TcII DTU strains showed a different pattern of chromosomal aneuploidies from each other and showed a smaller group consistency than the strains from the TcI DTU. The 231 TcIII DTU strain shows the most different pattern among the *T. cruzi* strains evaluated.

### Y Chromosome 11

**Fig. 6:**
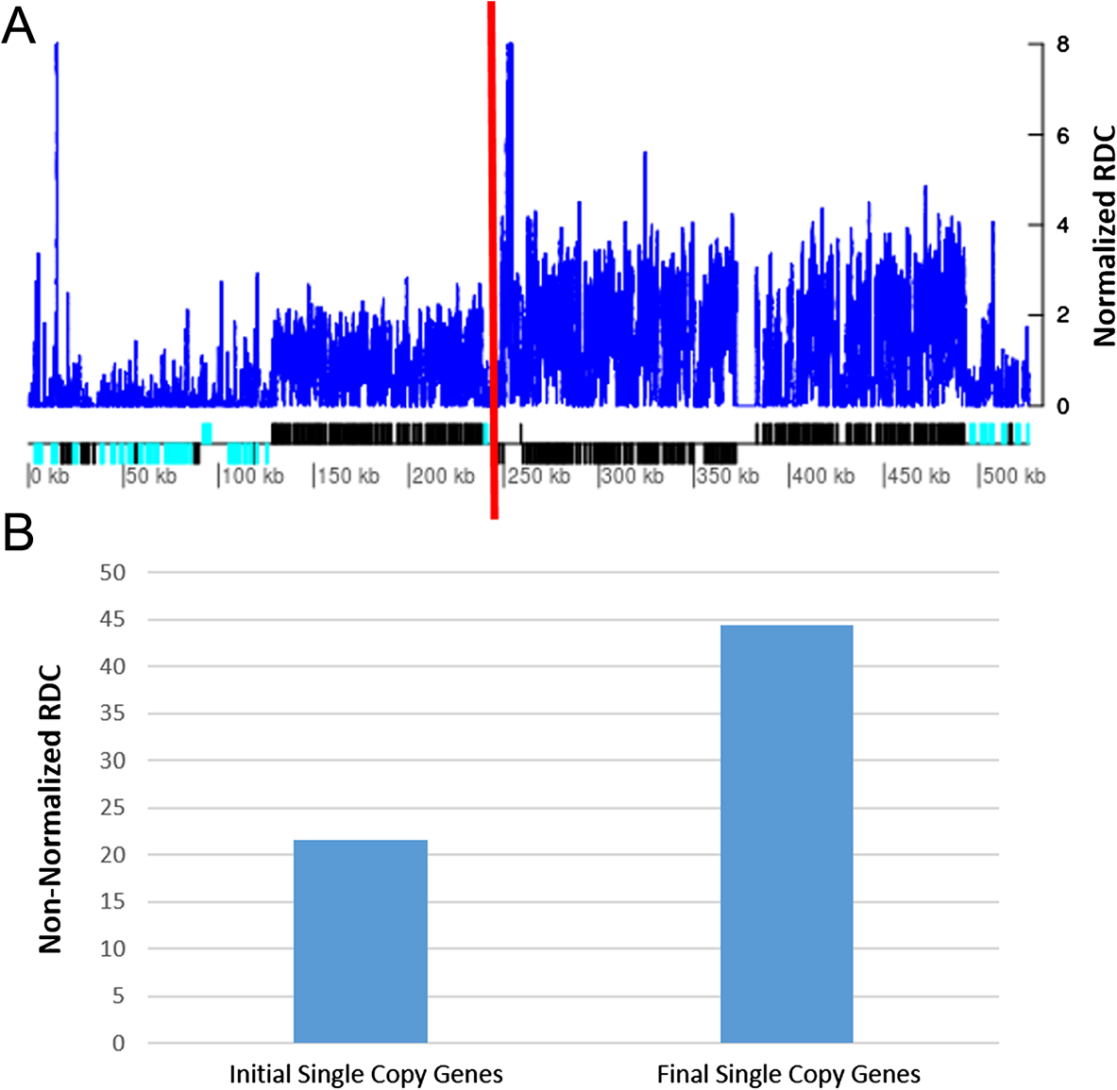
Predicted ploidy of chromosome 11 of the *T. cruzi* Y strain. The blue line corresponds to the normalized RDC of each position of chromosome 11, estimated by the ratio between the RDC and the genome coverage. Below, the protein-coding genes are depicted as rectangles drawn in proportion to their length, and their coding strand is indicated by their position above (top strand) or below (bottom strand) the central line. Black and cyan rectangles represent the housekeeping/hypothetical and multicopy gene families, respectively. Gaps are represented by gene-less regions with no read coverage. The red line corresponds to the 248-kb position in this chromosome, which separates the regions of low RDC and high RDC (**a**). The non-normalized mean RDC estimated based on the SCoPE approach of the genes that were upstream and downstream of the 248-kb position was estimated (**b**)

Chromosome 11 from the Y strain has some interesting features. As shown in Fig. 3, this chromosome displayed a smaller ploidy than disomic but a greater ploidy than monosomic. This led us to investigate whether this pattern occurs due to the loss of a chromosomal region instead of the loss of a chromosome copy. As showed in Fig. 6, there is a drastic change in the RDC starting at the 248-kb position in this chromosome that corresponds to a strand switch region. The first 248 kb of this chromosome has a smaller predicted ploidy when compared to the rest of the chromosome sequence (Fig. 6a). To further confirm this chromosomal region loss, the mean RDC of the single-copy genes located upstream and downstream of the 248-kb coordinate were estimated. The single-copy genes that were upstream of the 248-kb position had a non-normalized mean RDC of 20, while the single-copy genes downstream had an approximate mean RDC of 40 (Fig. 6b). This reduction of the RDC in the 5′ region when compared to the rest of the chromosome and the estimated genome coverage of 34× suggest a segmental loss of the 248 kb at the 5′ region in one copy of this chromosome in the Y strain. Alternatively, this pattern may be due to differences in the structure of chromosome 11 among the different cells of the parasite population because Y is not a cloned line.

### Chromosome 31 gene ontology

**Fig. 7:**
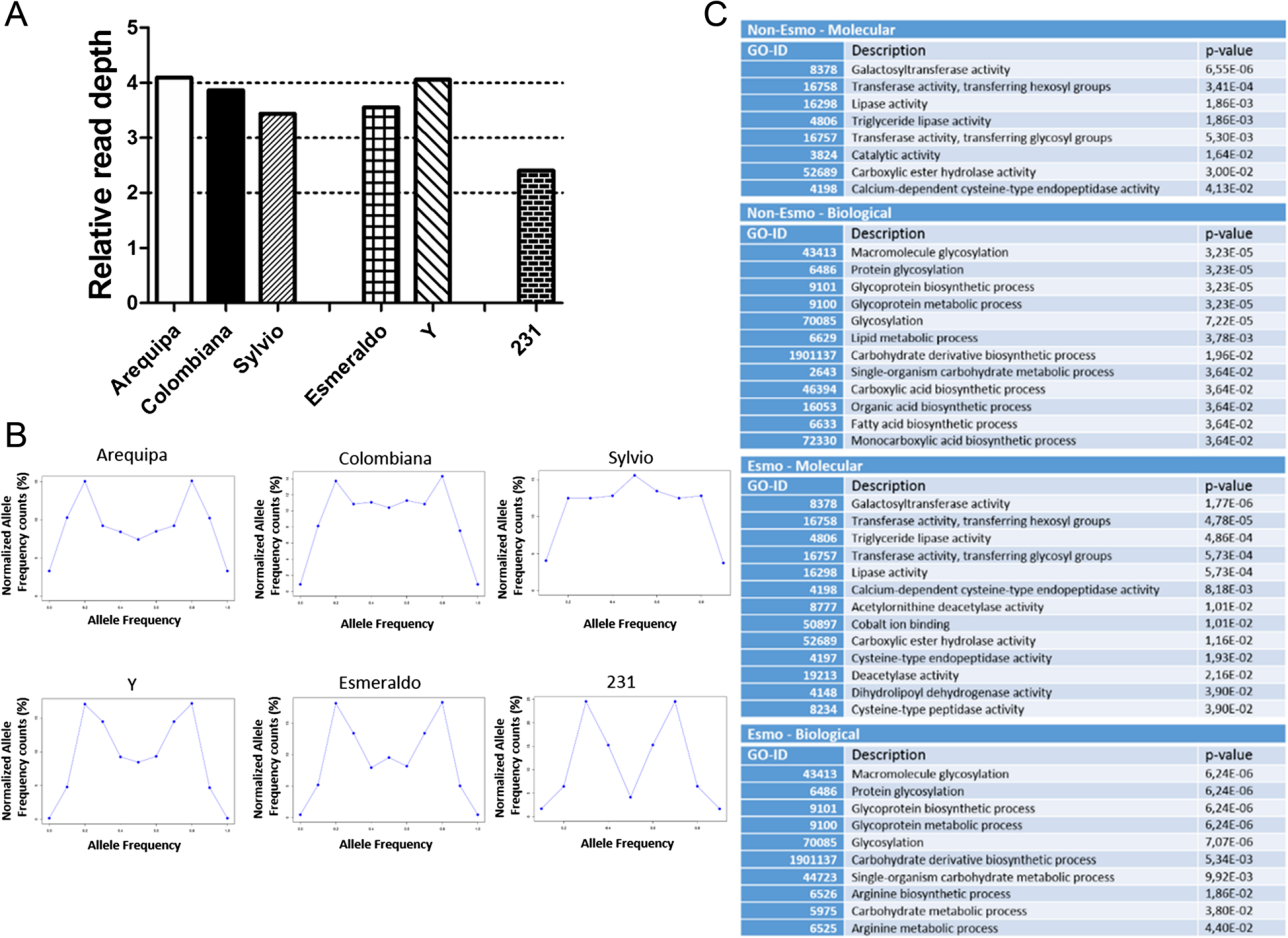
Ploidy and gene ontology analysis of chromosome 31. From the 41 CL Brener chromosomes, chromosome 31 was the only one that was supernumerary in all the studied strains based on the SCoPE analysis (**a**). The ploidy of chromosome 31 from each strain, estimated by the ratio of the alleles in the heterozygous SNP positions, classifies Arequipa, Colombiana, Sylvio, Esmeraldo and Y as tetraploid and 231 as triploid (**b**). Gene ontology analysis shows that this chromosome is enriched in genes involved in the glycosylation and glycoprotein biosynthetic processes (**c**)

From all the 41 CL Brener chromosomes, chromosome 31 was the only one that was supernumerary in all the strains analyzed, in both SCoPE (Fig. 7a) and heterozygous SNP analyses (Fig. 7b). To identify gene functions that were overrepresented in this chromosome when compared to the whole genome, a gene ontology analysis of both Esmeraldo-like and non-Esmeraldo-like CL Brener chromosome 31 was performed (Fig. 7c, Additional file 5: Table S3). This analysis shows that this chromosome is enriched in genes involved in glycosylation and glycoprotein biosynthetic processes in both CL Brener haplotypes.

## Discussion

The availability of the sequence representation of the 41 putative chromosomes from both Esmeraldo-like (TcII) and non-Esmeraldo (TcIII) CL Brener haplotypes [33] allowed a comparative analysis of *T. cruzi* strain ploidy using the unassembled next generation sequencing reads. However, the repetitive nature of the *T. cruzi* genome along with the presence of gap regions in the sequence representation of CL Brener chromosomes hampers the effectiveness of CCNV comparisons based on the read depth coverage (RDC) of whole-chromosome sequences. To overcome these limitations, we propose the SCoPE approach that is based on the RDC of single-copy genes that are conserved between Esmeraldo-like and non-Esmeraldo-like haplotypes as well as in the six *T. cruzi* strains evaluated in this study (Additional file 2: Table S2). These single-copy genes were used as chromosomal markers for unique genomic sequences to normalize the CCNV estimations, revealing large differences in chromosomal copy number between *T. cruzi* strains (Fig. 3).

It is well known that *T. cruzi* strains present a distinct profile of chromosomal bands, which present different sizes and numbers in Pulse Field Gel Electrophoresis, suggesting a variable karyotype among the DTUs. These differences were mainly attributed to a differential repetitive content in the genomes of distinct *T. cruzi* strains, or to chromosomal fusion/break events during the parasite evolution [5, 22, 30, 31, 35]. In fact, approximately 50 % of the CL Brener genome corresponds to repetitive sequences, many of them clustered into regions containing multigene families encoding surface proteins and transposable elements [20, 33]. These clusters are extremely variable in their gene content and length and are regions of synteny loss when comparing the two CL Brener haplotypes (El-Sayed 2005a) and the CL Brener and Sylvio genomes [21]. Despite these variations, single-copy genes are, in general, highly conserved and syntenic among the *T. cruzi* strains [8, 21, 22, 36] (Baptista in preparation) and therefore represent an adequate source for sequence normalization in comparative CCNV analyses.

The occurrence of whole chromosome aneuploidy in *T. cruzi* was previously suggested based on whole genome oligonucleotide tiling arrays and competitive genomic hybridization between 16 *T. cruzi* strains and CL Brener clone as reference [8]. However, the absolute number of individual chromosomes in each strain was not estimated [8]. Also, the comparative genomic hybridization analysis does not allow the detection of chromosomal expansion/deletions that are present simultaneously in CL Brener and in the tested strain [8]. Our approach, however, used the haploid content of CL Brener chromosomes as reference for read mapping, allowing the detection of chromosomal copy number variations that may be also present in CL Brener.

### Competitive mapping

To identify the CL Brener haplotype suitable to be used as a reference, each *T. cruzi* strain read collection from the TcI (Arequipa, Colombiana and Sylvio), TcII (Esmeraldo and Y) and TcIII (231) strains was simultaneously mapped to both Esmeraldo-like and non-Esmeraldo-like CL Brener haplotypes, using a competitive approach (Fig. 1a). As the mapping quality cutoff was very stringent, the conserved regions that mapped simultaneously to both CL Brener haplotypes were excluded, and only reads that mapped preferentially to one haplotype were considered. As expected, reads from the TcII strains, namely Esmeraldo and Y, mapped better to the Esmeraldo-like haplotype (CL Brener haplotype derived from a TcII ancestor genome), and reads from TcIII mapped better to the non-Esmeraldo haplotype (derived from the TcIII ancestor) (Fig. 1b). All strains belonging to the TcI DTU—Arequipa, Colombiana and Sylvio—mapped slightly better to the non-Esmeraldo haplotype, suggesting a closer proximity to TcI compared to TcIII as previously described [11, 14, 15, 21]. Interestingly, all TcI strains had a higher RDC for the Esmeraldo-like chromosome 14 than for the same chromosome from the non-Esmeraldo haplotype. Another example is the Esmeraldo chromosome 3, which had a slightly better RDC for the non-Esmeraldo than for the Esmeraldo-like. A careful inspection of the RDC along the entire sequences of these chromosomes revealed that this unexpected profile was due to an extremely high RDC of a specific region in both chromosomes. In the case of chromosome 14, the genomic region encompassing the gene Tc00.10470535007099.80 (ABC transporter putative) in the Esmeraldo-like haplotype displays a very high RDC, and this region is absent in the non-Esmeraldo-like haplotype. Therefore, it is likely that this is a repetitive sequence where reads from both CL Brener haplotypes were collapsed in the assembled Esmeraldo-like haplotype. Likewise, in the case of chromosome 3, approximately 1500 nucleotides of the 5′ end of the gene Tc00.1047053508533.10 (hypothetical protein conserved) from the non-Esmeraldo-like haplotype are missing in the corresponding allele (Tc00.1047053404001.20) of the Esmeraldo-like haplotype. These genes are not included in the 1563 copy genes used by SCoPE to estimate the CCNV, further validating this methodology.

### Chromosomal copy number variation and strain ploidy

All six *T. cruzi* strains evaluated in this study had an overall genome ploidy predicted as diploid, based on both the SCoPE approach and the allele proportion in heterozygous positions, when the whole genome was evaluated (Fig. 3). This result is in agreement with previous estimations for *T. cruzi* [8, 9, 37] and *Leishmania* species that classified these parasites as diploid, with the exception of *L. braziliensis* (M2904) that had an overall trisomic chromosomal pattern [34].

The CCNV among *T. cruzi* strains was initially estimated by the SCoPE approach (Fig. 3). The strains from the TcI DTU (Colombiana, Sylvio and Arequipa) had a low number of whole chromosome expansions (1, 1 and 3, respectively), while the TcII strains (Esmeraldo and Y) and the TcIII strain (231) had a larger number of these expansions (13, 6 and 12, respectively). These predictions were further confirmed by the heterologous SNP analysis, which predicted the same aneuploidies, with the exception of chromosomes 20 and 23 from Sylvio, 7 from Esmeraldo and 6 and 14 from Arequipa that were predicted as tetrasomic. In these chromosomes, the heterozygous SNP analysis predicted an even broader chromosomal expansion than those estimated by the SCoPE approach. To evaluate if the CCNV predicted using the single-copy genes was not impacted by segmental duplications or loss of partial fragments from these chromosomes, we estimated the RDC of all the positions from all 41 chromosomes of the six *T. cruzi* strains (Fig. 4, Additional file 4: Figure S2). Excluding chromosomal gaps and regions containing clusters of multigene families, the RDC of the whole chromosome is in agreement with the SCoPE CCNV prediction, validating this approach (Fig. 4).

Gene copy number variation is well documented as an important mechanism to enhance gene expression and variability not only in *T. cruzi*, *but also in T. brucei and Leishmania* [8, 20, 28, 36, 38–40]. The CCNV arises as a new level of CNV, expanding whole blocks of genes simultaneously [34]. The occurrence of CCNV was already associated to increased fitness in stress conditions and to drug resistance in *Saccharomyces cerevisiae* and *Candida albicans* [41–43]. The karyotype plasticity and CNV found in *T. cruzi* could also represent a framework for the natural selection of favorable phenotypes, such as higher expression of virulence-factors and increased diversity, resulting in an enhanced adaptability of the parasite [8, 44].

The mechanisms involved in the generation of CCNV in *T. cruzi* are still unknown. Even though it has been previously proposed that meiosis events occur in *Trypanosoma brucei* [45] and *Leishmania* [46], this process has not been demonstrated in *T. cruzi*. However, a non-meiotic hybridization model has been proposed to explain the generation of hybrid *T. cruzi* DTUs [47]. According to this model, during the mammalian stage of the parasite, the nucleus of two diploid cells fuse, resulting in a polyploidy progeny that can undergo recombination between alleles. This new polyploid cell may lose some of its supernumerary chromosomal copies, eventually returning to the diploid state [48]. This could be one of the mechanisms related to the generation of CCNV in *T. cruzi*, as some of these extra chromosomal copies may be maintained by the parasite.

For all the *T. cruzi* DTUs evaluated, TcI had the fewest number of chromosomal expansions, exhibiting the most stable karyotype (Fig. 3). The reduced number of chromosomal duplications could be a contributing factor for the low levels of heterozygosity found in TcI [21, 49]. It has been demonstrated that the mismatch repair machinery, which repairs base miss-incorporation and erroneous insertions and deletions during DNA recombination and replication, is more efficient in TcI than in the other DTUs and is a known factor in the reduction of heterozygosity in TcI [50, 51]. TcI strains also have the smallest genome size compared to the other *T. cruzi* DTUs [6], which were associated with the reduction of multigene family clusters within this DTU [21]. Based on our results, we propose that this reduction in genome size is also associated with the lower number of chromosomal duplications in TcI when compared to other DTUs. Similarly, data from Rogers 2011 showed a correlation between CCNV based on RDC analysis and DNA content estimated using flow cytometry in different *Leishmania* species [34]. Variation in DNA content among *T. brucei* species and isolates was also observed [52] and could be related with some level of CCNV in this parasite, which so far has not been formally demonstrated. Although TcI strains have less intragenomic heterogeneity, sequences belonging to different strains are more distant from each other within TcI than within TcII and TcVI [8, 23]. This increased variation suggests a reduced number of genetic exchange events among strains from the TcI DTU, which could result in a lower rate of chromosomal ploidy variations.

For the TcII DTU, Esmeraldo had 13 chromosomal expansions while Y had 6, suggesting that there are extensive differences in chromosomal duplications within TcII DTU (Fig. 3). Recently, substantial recombination and genetic exchange among strains from the TcII DTU that coexist in the same geographical area was proposed based on microsatellite genotype data [19]. The broader chromosomal expansion in Esmeraldo may be explained by the fact that haplotypes that constitute this strain are more distant from each other than the ones that constitute Y [15], which suggests that Esmeraldo suffered more recombination events than Y, making it more susceptible to acquiring aneuploidies [15, 48]. Leishmania isolates also display a broad range of CCNV. As TcI DTU strains evaluated in this study, different strains of *L. major* (Friedlin and LV39) had the same CCNV pattern. On the other hand, as observed in TcII DTUs, strains from both *L. mexicana* and *L. donovani* had extensive CCNV within the species [34]. It will be interesting to compare the efficiency of processes associated with the maintenance of genomic stability in *T. cruzi* DTUs and *Leishmania* species, and investigate the occurrence of CCNV in *T. brucei*, which could help to elucidate the mechanisms behind the CCNV in these parasites. Widespread aneuploidy found in *T. cruzi* and *Leishmania*, implies that caution should be taken when selecting markers for population genetic studies based on the hypothesis of diploidy [13]. In this case, it would be imperative selecting markers from genomic regions known to be diploid.

Hierarchical clustering analysis based on the predicted ploidy of each *T. cruzi* chromosome clustered all TcI strains together with high confidence scores, further confirming the genome structural stability within DTUI (Fig. 5). Aside from the TcI DTU, strains from the TcII and TcIII DTUs had a variable CCNV pattern, suggesting a higher genome plasticity between and within these DTUs. It is interesting that the two strains from the TcII DTU (Esmeraldo and Y) had a different pattern of chromosomal ploidy, suggesting that chromosomal expansions are highly variable and may have originated several times during the evolution of the TcII DTU. The analysis of a broader number of strains would be required to correctly estimate the ratio of CCNV within and between the *T. cruzi* DTUs.

### Y chromosome 11

In the first 248 kb of the Y chromosome 11, we identified 22 single-copy genes, which had half the mean RDC compared with the remaining chromosome sequence, which contains 15 single-copy genes (Fig. 6). This large change in RDC starts in a strand switch region, which is frequently associated with rearrangements in Trypanosomatid genomes [36, 53]. This finding suggests that Y chromosome 11 could have an arm loss. Another possibility is that the CL Brener chromosome 11 is divided into two distinct chromosomes in Y, chromosomes 11a and 11b, at the 248-kb position, where 11a is haploid and 11b is diploid. Events of chromosomal break or fusion may explain the variable band pattern in PFGE among the *T. cruzi* strains [5, 22, 30–32, 35]. These events are easily detected in RDC analysis when there are large aneuploidies between fragments of the same chromosome. Alternatively, because Y is not a cloned population, this result may represent a mosaic structure of the parasite population, where some cells may have the entire chromosome 11 sequence while other cells may have an arm loss.

### Chromosome 31

Chromosome 31 was the only one that was supernumerary in all six *T. cruzi* samples evaluated in this study (Fig. 7). Gene ontology analysis showed that this chromosome has an enhanced number of genes related to glycoprotein biosynthesis and glycosylation processes (Fig. 7c). CL Brener has approximately 100 genes annotated as putative glycosyltransferase genes, which are involved in the synthesis of a variety of glycoconjugates and are abundantly and differentially expressed in all *T. cruzi* stages [54]. From these 100 genes, 54 are UDP-GlcNAc-dependent glycosyltransferases. Chromosome 31 has 9 of the 27 UDP-GlcNAc-dependent glycosyltransferase gene copies in the CL Brener Esmeraldo-like haplotype and 13 of the 27 copies in the non-Esmeraldo haplotype. This enzyme is involved in the transfer of N-acetylglucosamine (GlcNAc) from the UDP-GlcNAc precursor to the hydroxyl group of serine and threonine residues, resulting in O-linked oligosaccharides in *T. cruzi* mucins [55]. Comparative genomic hybridization among *T. cruzi* strains also shows gene CNV in another gene involved in the synthesis of glycans on *T. cruzi* mucins, the beta-galactofuranosyl transferase genes [8, 56]. Mucins are heavy glycosylated glycoproteins, and their glycan content may account for up to 60 % of the total mucin weight [55, 57]. These proteins are the most abundant component on the *T. cruzi* surface, covering the whole parasite with approximately 2 × 10^6^ copies per cell [39, 55]. Mucins are responsible for protecting the parasite from both the vector and the mammal defensive mechanisms and ensure the anchorage point and invasion of specific cells and tissues [55]. One of the forces driving the expansion of chromosome 31 in all the *T. cruzi* strains may be the need to glycosylate this large number of proteins that cover the parasite surface and are directly involved in parasite survival in both invertebrate and vertebrate hosts. Although chromosome 31 was also expanded in several *Leishmania* species [34], we found no large syntenic regions between *T. cruzi* and *Leishmania* chromosome 31, suggesting that the chromosome 31 expansion in *Leishmania* is driven by different evolutionary pressures.

## Conclusions

It is well known that *T. cruzi,* as well as *Leishmania* and *T. brucei*, relies on gene duplication to increase the expression levels of key genes and to allow the generation of novel genes without loss of function [8, 20, 28, 36, 38–40]. Our study highlights the genome-wide CCNV in *T. cruzi* as a new level of gene expansion mechanism, allowing the rapid generation of diversity within the parasite. The estimation of chromosomal aneuploidies based on the RDC of single-copy genes comes as a new approach to evaluate the CCNV in *T. cruzi*, reducing the bias of repetitive and gap regions in the analysis and improving chromosomal comparisons between DTUs. As previously observed in *Leishmania* [34], aneuploidy appears to be well tolerated in trypanosomatids, due to their predominantly asexual replication mechanism. The chromosome copy number can vary considerably between strains from different *T. cruzi* DTUs and even within the same DTU. TcI appears to be more stable, and TcII had large differences between its strains, suggesting that this mechanism is widely used by the parasite to expand groups of genes. One of the limitations of our approach is that we are not able to investigate the karyotype structure of each strain, and the analysis is limited to comparing their differences based on the CL Brener predicted chromosomes because there are no other *T. cruzi* chromosomal sequences already published in the literature. Due to the extensive repetitive content, third-generation long read single-molecule sequencing is required not only to close the gaps in CL Brener chromosomes, but also to generate reliable chromosomal sequences of the other five DTUs allowing better CCNV estimations. Only three DTUs (TcI, II and III) were evaluated in this work. Therefore, it would be interesting to evaluate CCNV in the other *T. cruzi* DTUs, to better investigate this variation in the parasite. Finally, the expansion of chromosome 31, which is enriched with genes related to glycosylation pathways in all six strains evaluated, highlights the importance of this biochemical process to the parasite’s survival.

## Methods

### Parasite cloning in a semi-solid medium

For cloning the *T. cruzi* Arequipa (TcI) and 231 (TcIII) strains, 10^3^ epimastigotes were plated into a semi-solid medium (low-melting agarose 0.75 %, brain heart infusion 48.4 %, liver infusion tryptose (LIT) 48.4 %, 2.5 % defibrinated blood, and 250 μg/mL penicillin/streptomycin) and incubated at 28 °C for 35 days. Single clones were obtained and transferred to 25-cm^3^ culture flasks with 5 mL of LIT medium and 10 % fetal bovine serum.

### Parasite culture and DNA isolation

*T. cruzi* epimastigotes from Arequipa (TcI), Colombiana (TcI) and Y (TcII) strains were cultured in LIT medium supplemented with 10 % fetal bovine serum. A total of 1 × 10^8^ parasites from each strain were centrifuged at 3000 g in an Eppendorf 5804 Centrifuge. The parasites where washed three times with ice-cold PBS, suspended in PBS with 100 μg/mL proteinase K and incubated at 25 °C for 10 min. The genomic DNA was obtained with the Wizard® Genomic DNA Purification Kit (Promega) by following the manufacturer instructions. The DNA integrity was evaluated by agarose gel electrophoresis. The DNA samples were submitted to a genotyping protocol according to Souto et al., 1996 [58], de-Freitas et al., 2006 [15] and Burgos et al., 2007 [59].

### Genome sequencing

A whole-genome shotgun library (WGSG) and sequencing of the *T. cruzi* Arequipa (TcI), Colombiana (TcI) and Y (TcII) strains were performed at the Computational Genomics Unity Darcy Fontoura de Almeida (UGCDFA) of the National Laboratory of Scientific Computation (LNCC) (Petrópolis, RJ, Brazil). For the 454 GS-FLX Titanium sequencing, each unpaired library was constructed using 5 μg of genomic DNA (gDNA) and by following the GS FLX Titanium series protocols. All titrations, emulsions, PCR, and sequencing steps were carried out according to the manufacturer’s protocol. One full PicoTiterPlate (PTP) was used for sequencing each library. In addition to the 454 sequencing, the Ion Proton™ was also used for unpaired sequencing. A total of 1 μg of gDNA was used to prepare the deep sequencing libraries. All steps were also performed according to manufacturer’s protocol. Reads from Sylvio (TcI) were kindly provided by Dr. Bjorn Andersson (Karolinska Institut). The 231 (TcIII) sequences were obtained using the Illumina Hiseq 2000 NGS platform (Baptista et al., in preparation). The Illumina and 454 read libraries from the Esmeraldo strain were downloaded from the National Center for Biotechnology Information (NCBI) (Additional file 6: Table S4).

### Preprocessing of reads

The reads were checked for quality using the FASTQC tool (http://www.bioinformatics.babraham.ac.uk/projects/fastqc/). Reads smaller than 30 nt and with a Phred score lower than 20 [60, 61] were removed from the libraries using the fast_quality_trimmer, from the fastx toolkit (“http://hannonlab.cshl.edu/fastx_toolkit/index.html).

### Mapping and competitive mapping

Whole genome shotgun reads from the *T. cruzi* Arequipa, Colombiana, Sylvio, Esmeraldo, Y, and 231 strains were mapped to the 41 chromosomes from both Esmeraldo-like and non-Esmeraldo-like CL Brener haplotypes, version 6, downloaded from Tritrypdb (http://tritrypdb.org/tritrypdb/). Mapping for each read library was performed simultaneously for both CL Brener haplotypes for competitive mapping, or separately for single mapping, using Bowtie 2 [62]. To account for the divergence between strains, the Bowtie2 preset “very sensitive” parameter was used, with the mismatch parameter changed to 1. The competitive mapping was used to select the CL Brener haplotype closely related to each read library, and single mapping was used to estimate the CCNV in each strain. Mapped reads of each strain were filtered using a mapping quality threshold of 30 using SAMtools v1.1 [63]. The read depth coverage (RDC) for each position of each chromosome of the Esmeraldo-like and non-Esmeraldo-like haplotypes for each *T. cruzi* strain was obtained by an in-house PERL script and BEDtools genomecov v2.16.2 [64]. The competitive mapping graphs were generated using GraphPad Prism V5.01 and scripts developed in PERL and R.

### Single-copy genes and chromosomal ploidy

Ortholog genes between Esmeraldo-like and non-Esmeraldo-like CL Brener haplotypes were identified using OrthoMCL v2.0 [65], based on a combined approach of “reciprocal best hits” and a “Markov clustering algorithm” (MCL). Initially, an “all vs. all” local alignment using the BLASTp package 2.2.21 with an E-value of 1e-5 as a cut-off was performed. The E-values were converted into log base to create the similarity matrix. An MCL with a 1.5 inflation parameter was applied to produce the ortholog clusters. A total of 1563 1:1 orthologs were selected and assumed to be single-copy genes in the haploid CL Brener genome (Additional file 1: Table S1). These single copy genes were selected to be used as chromosomal markers for unique genomic sequences, allowing CCNV estimations without the bias of repetitive regions. The mean RDC of the single-copy genes in each of the 41 CL Brener chromosomes, based on the mapped reads from the Arequipa, Colombiana, Sylvio and 231 strains to the non-Esmeraldo-like haplotype, and the Esmeraldo and Y to the Esmeraldo-like haplotype, were generated by PERL scripts. The mean RDC of all single-copy genes in all chromosomes of each strain was assumed to be the genome coverage. The predicted copy number of each chromosome was determined based on the mean RDC of the single-copy genes in a given chromosome and normalized by the genome coverage. The genome coverage was estimated as 47× for Arequipa, 28× for Colombiana, 9× for Sylvio, 52× for Esmeraldo, 34× for Y and 76× for 231. The CCNV graphs were generated with GraphPad Prism V5.01 software and scripts in PERL and R.

### SNP content

Single-nucleotide polymorphisms (SNPs) of the mapped reads from the single-copy genes in the *T. cruzi* Arequipa, Colombiana, Sylvio, Esmeraldo, Y and 231 strains to the CL Brener Esmeraldo-like and non-Esmeraldo-like haplotypes were obtained using the SAMtools function mpileup [63]. To reduce the chance of incorrectly identifying a SNP due to sequencing artifacts, we set the minimum number of mapped reads to 10. To reduce the bias of collapsed regions, the maximum number of reads mapped in a SNP position was set to double the genome coverage of the corresponding genome. The SNP density for each strain was calculated and plotted using GraphPad Prism V5.01.

### Heterozygous SNPS

Heterozygous SNPs between the CL Brener chromosome and the mapped reads for the six *T. cruzi* stains were obtained from the filtered SAMtools mpileup results [63]. To be considered as a reliable SNP, the position RDC must be at least 10. To reduce the bias of collapsed regions, the position RDC must also be lower than twice the genome coverage. For each chromosome, the proportion of the alleles in each predicted heterozygous site was obtained and rounded to the first decimal place. Base frequencies were rounded in ten categories, ranging from 0.1 to 1, and an approximate distribution of base frequencies for each chromosome was plotted in R. To estimate the overall ploidy of each genome, the same methodology was applied, but the heterozygous positions from all CDSs from all chromosomes were employed simultaneously.

### Cluster dendrogram

A hierarchical clustering analysis of all predicted *T. cruzi* chromosomal ploidy was performed using the Pvclust package [66] implemented in R (www.r-project.org) (R Development 2010). First, a distance matrix was built with pairwise Euclidean distances between the strains and the dendrogram was generated by the complete linkage method. To assess the uncertainty in hierarchical cluster analysis, we used the two bootstrap resampling methods implemented in Pvclust: bootstrap probability (BP) by ordinary bootstrap resampling and the approximately unbiased (AU) probability from multiscale bootstrap resampling, which provides better estimations than BP. Both methods were calculated with 10,000 iterations.

### Gene ontology

Gene ontology categories that were significantly overrepresented in the genes of the CL Brener chromosome 31 were detected using the hypergeometric distribution analysis in BiNGO [67] with Benjamini and Hochberg false discovery rate correction.

### Availability of supporting data

The Sequence Read Archives (SRA) supporting the results of this article are available in the NCBI GenBank repository, accession numbers: Arequipa(SRS838181), Colombiana(SRS841912), Esmeraldo(SRR833799, SRR833800, SRR058517, SRR058509, SRR058520, SRR058518, SRR058519, SRR058515, SRR058516, SRR058513, SRR058514, SRR058510, SRR058511, SRR058512) and Y(SRS842149). The 231 strain SRA is available ate the European Nucleotide Archive repository, by the accession number: PRJEB9129.

## Additional files

Additional file 1: Table S1.
*T. cruzi* CL Brener single copy genes. Additional file 2: Table S2. Normalized RDC of the single copy genes from all six *T. cruzi* strains evaluated. Additional file 3: Figure S1. Heterozygous SNPs proportion predicted ploidy of all the chromosomes from the *T. cruzi* strains: Arequipa, Colombiana, Sylvio, Esmeraldo, Y and 231. Additional file 4: Figure S2. Normalized RDC of each position on the 41 CL Brener chromosomes by the reads from the *T. cruzi* strains: Arequipa, Colombiana, Sylvio, Esmeraldo, Y and 231. Additional file 5: Table S3. Gene ontology analysis of the Esmeraldo-like and Non-Esmeraldo chromosome 31. Additional file 6: Table S4.
*Trypanosoma cruzi* strains sequencing information.
