# Supplementary material for: Chromosomal copy number variation reveals differential levels of genomic plasticity in distinct Trypanosoma cruzi strains
Source: BMC Genomics. 2015 Jul 4;16(1):499. doi: 10.1186/s12864-015-1680-4 (PMC4491234; doi:10.1186/s12864-015-1680-4)
Supplement: Additional file 3: Figure S1. — Heterozygous SNPs proportion predicted ploidy of all the chromosomes from the T. cruzi strains: Arequipa, Colombiana, Sylvio, Esmeraldo, Y and 231. [file 12864_2015_1680_MOESM3_ESM.pptx]

## Slide 1
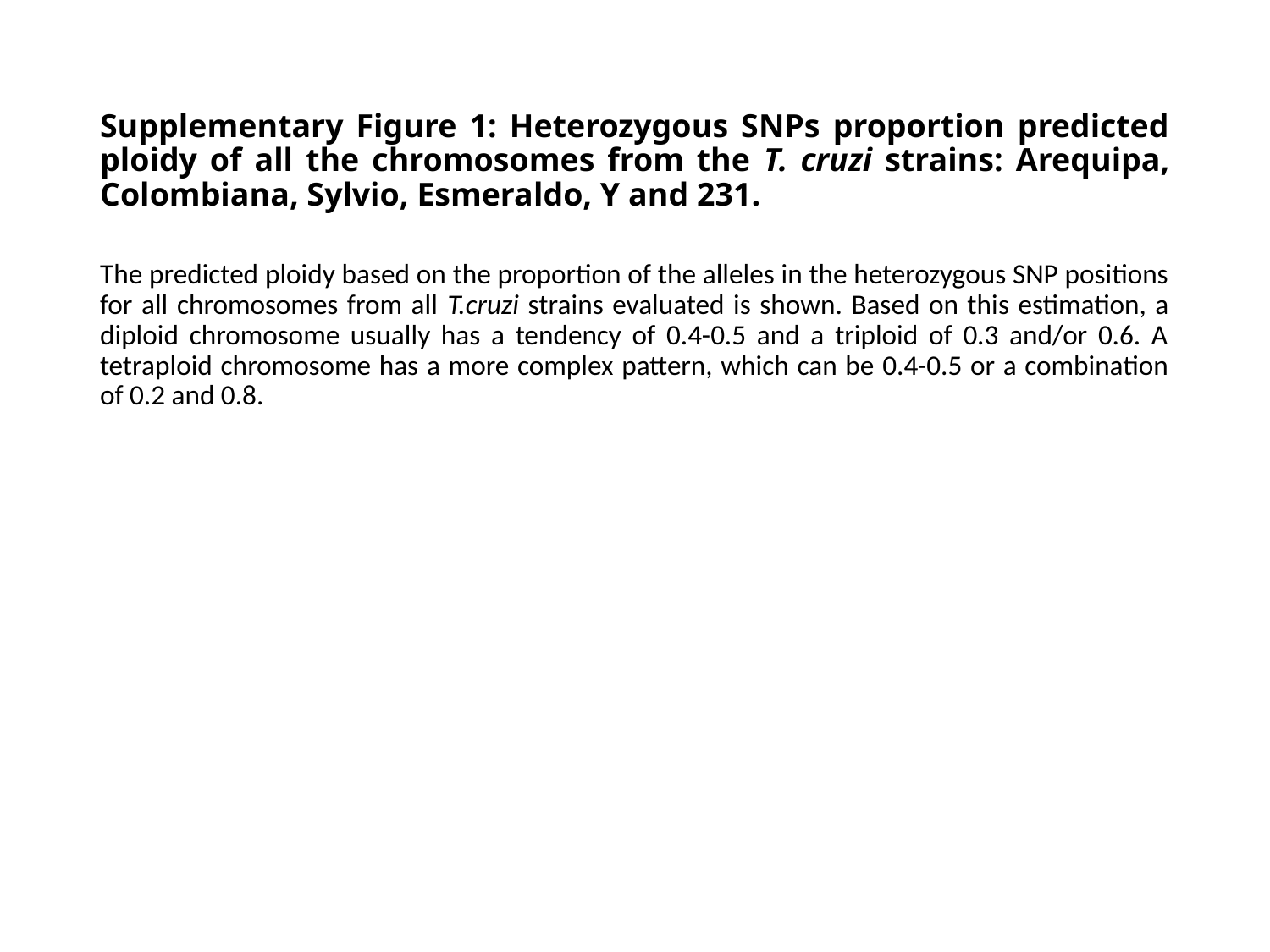

# Supplementary Figure 1: Heterozygous SNPs proportion predicted ploidy of all the chromosomes from the T. cruzi strains: Arequipa, Colombiana, Sylvio, Esmeraldo, Y and 231.
The predicted ploidy based on the proportion of the alleles in the heterozygous SNP positions for all chromosomes from all T.cruzi strains evaluated is shown. Based on this estimation, a diploid chromosome usually has a tendency of 0.4-0.5 and a triploid of 0.3 and/or 0.6. A tetraploid chromosome has a more complex pattern, which can be 0.4-0.5 or a combination of 0.2 and 0.8.

## Slide 2
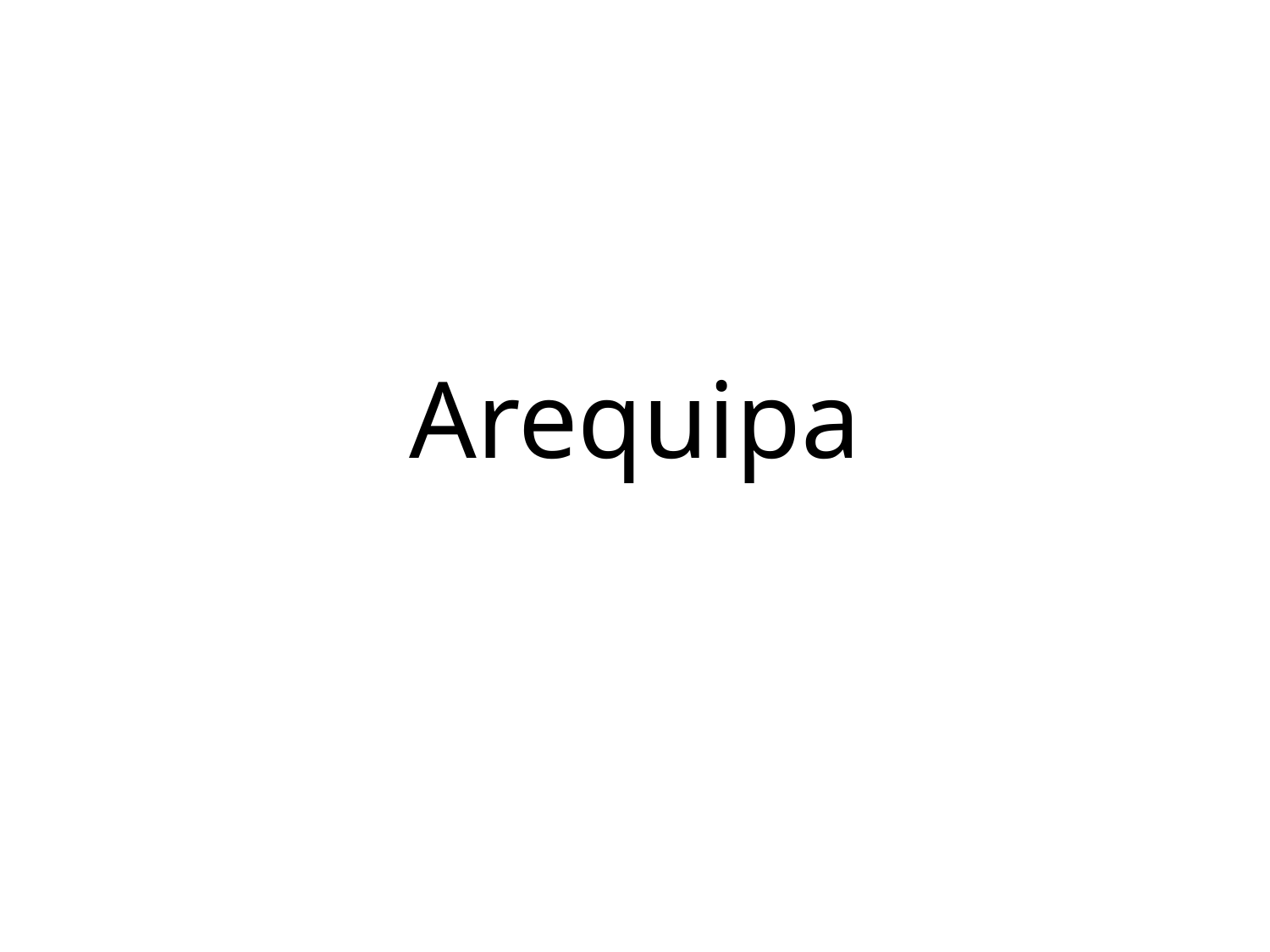

# Arequipa

## Slide 3
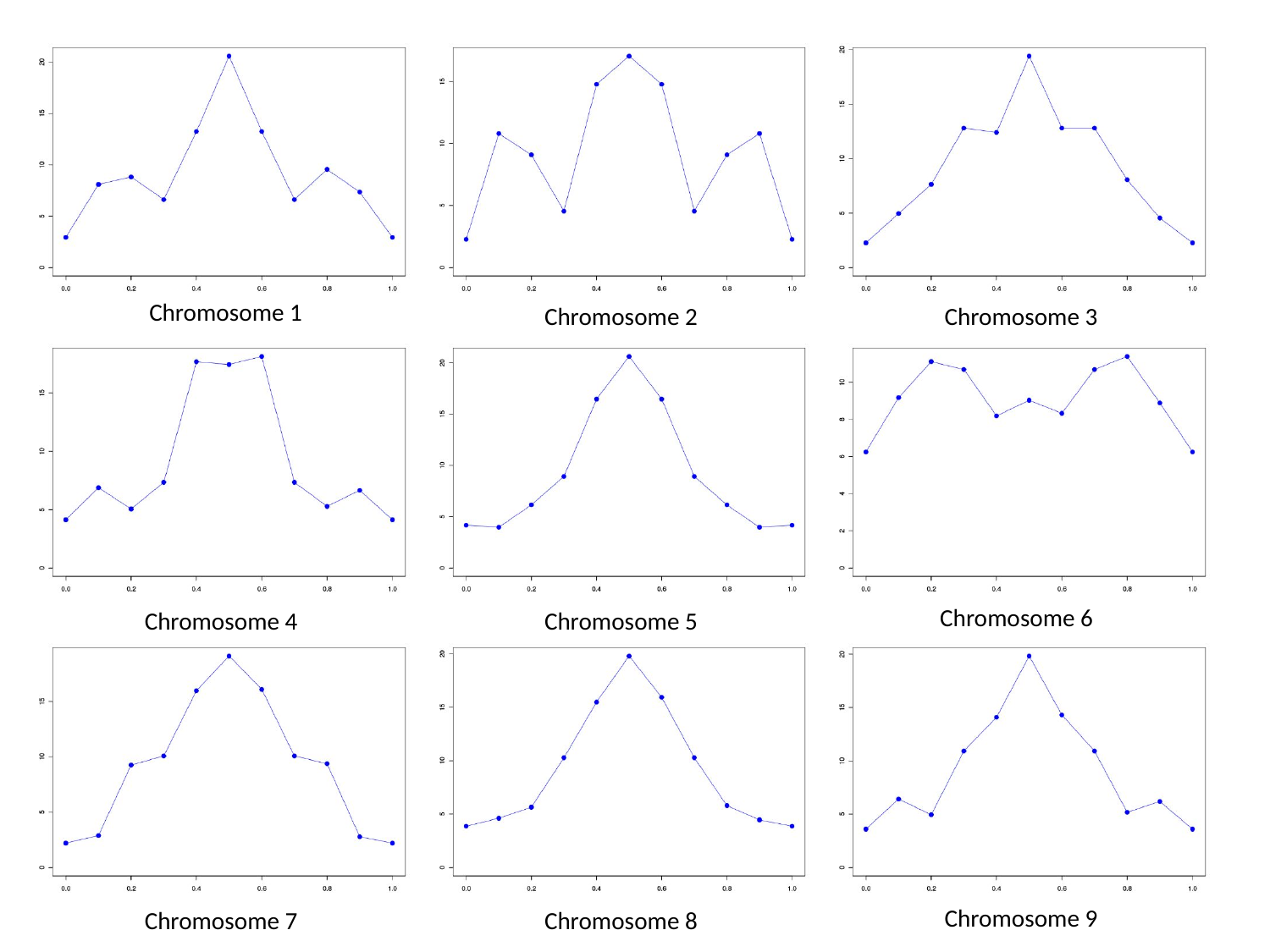

Chromosome 1
Chromosome 3
Chromosome 2
Chromosome 6
Chromosome 5
Chromosome 4
Chromosome 9
Chromosome 8
Chromosome 7

## Slide 4
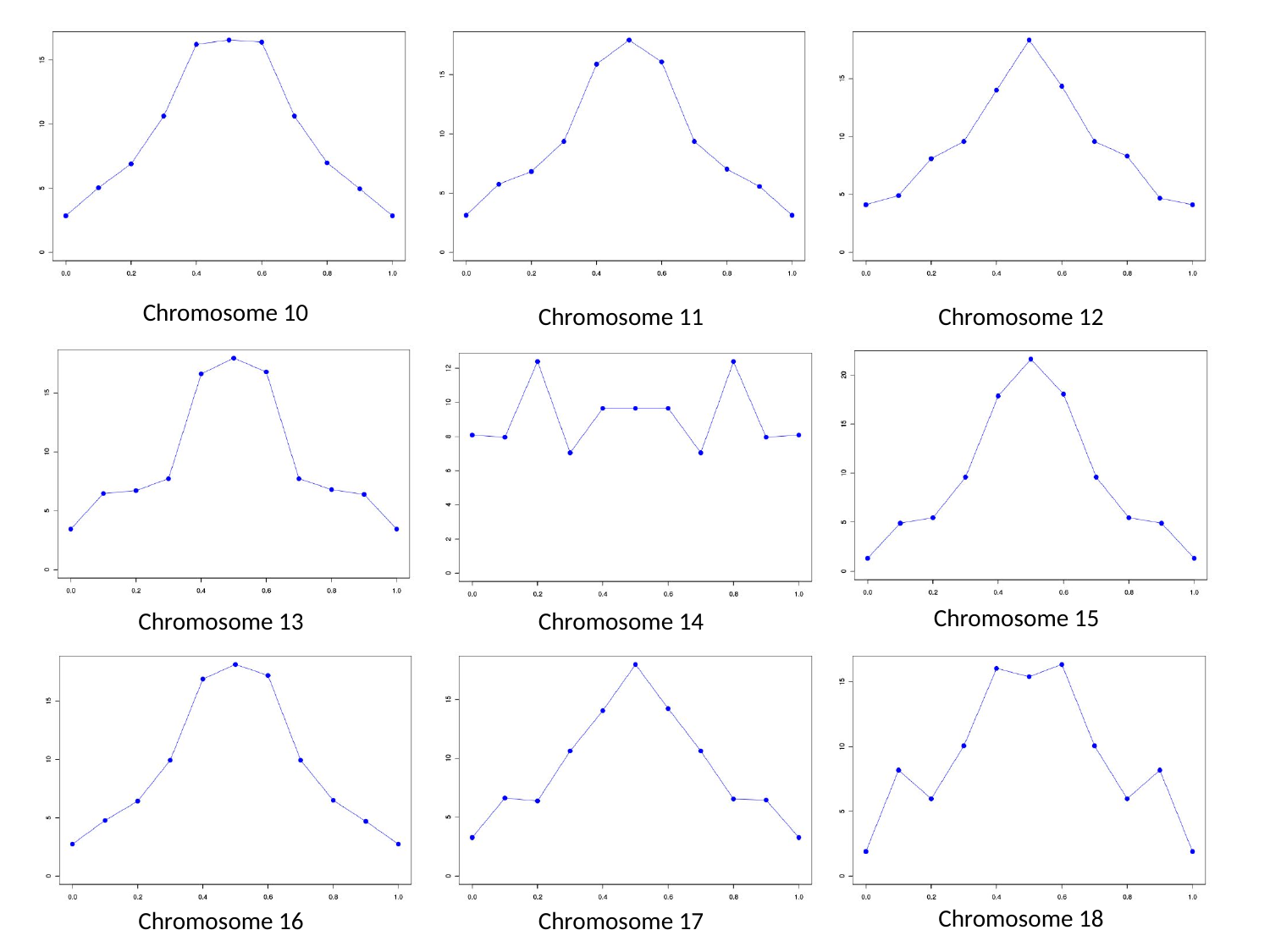

Chromosome 10
Chromosome 12
Chromosome 11
Chromosome 15
Chromosome 14
Chromosome 13
Chromosome 18
Chromosome 17
Chromosome 16

## Slide 5
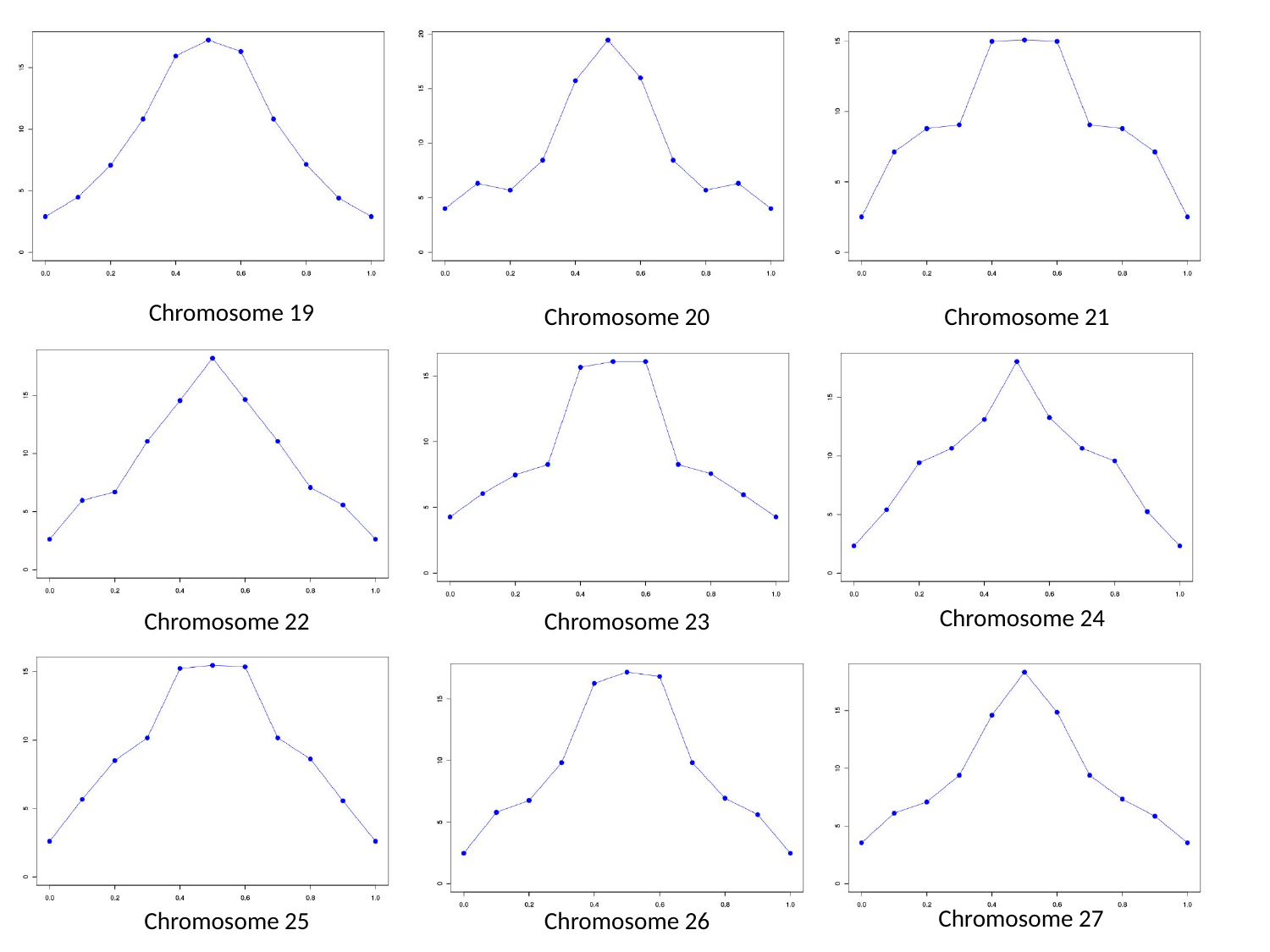

Chromosome 19
Chromosome 21
Chromosome 20
Chromosome 24
Chromosome 23
Chromosome 22
Chromosome 27
Chromosome 26
Chromosome 25

## Slide 6
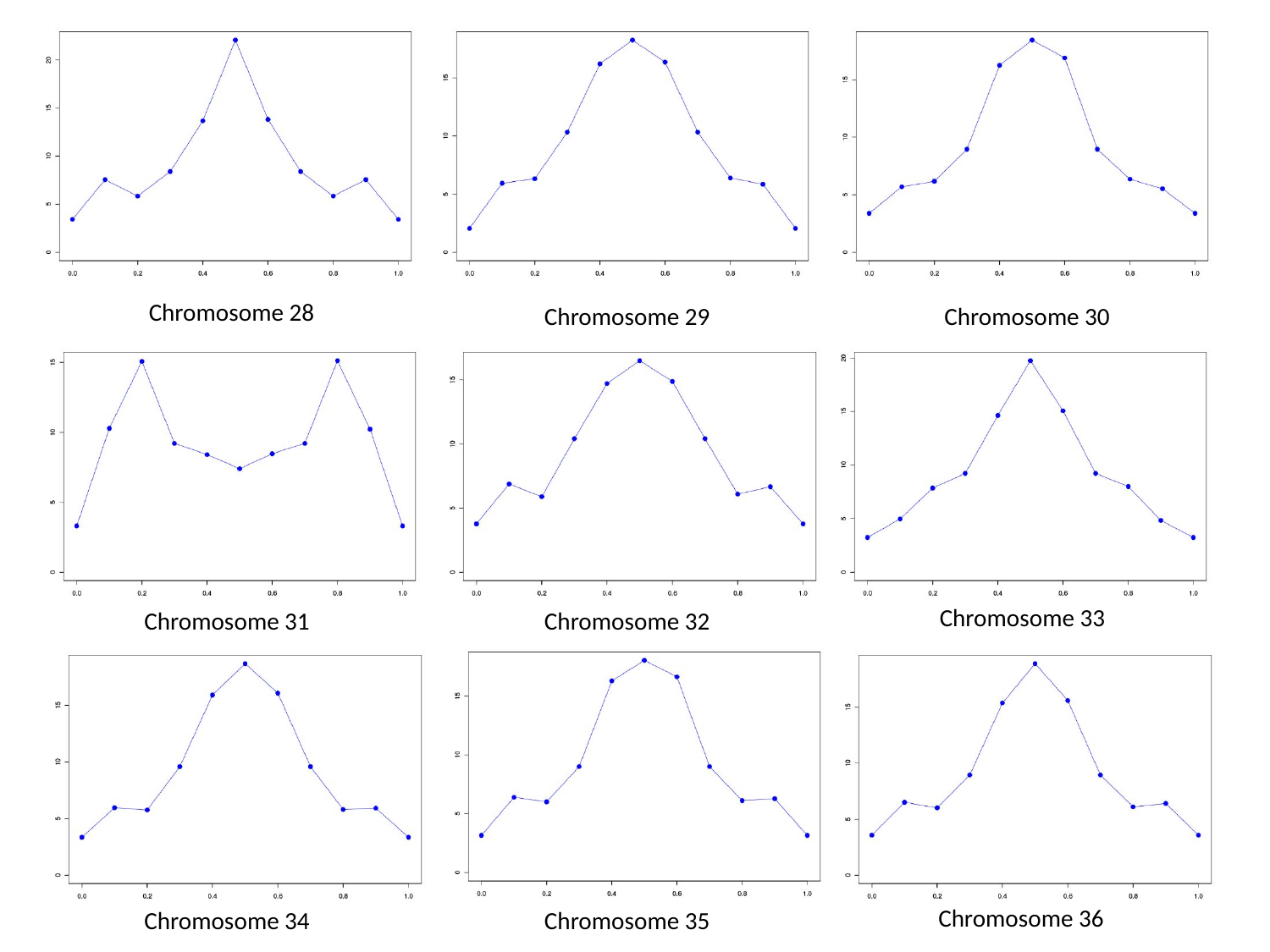

Chromosome 28
Chromosome 30
Chromosome 29
Chromosome 33
Chromosome 32
Chromosome 31
Chromosome 36
Chromosome 35
Chromosome 34

## Slide 7
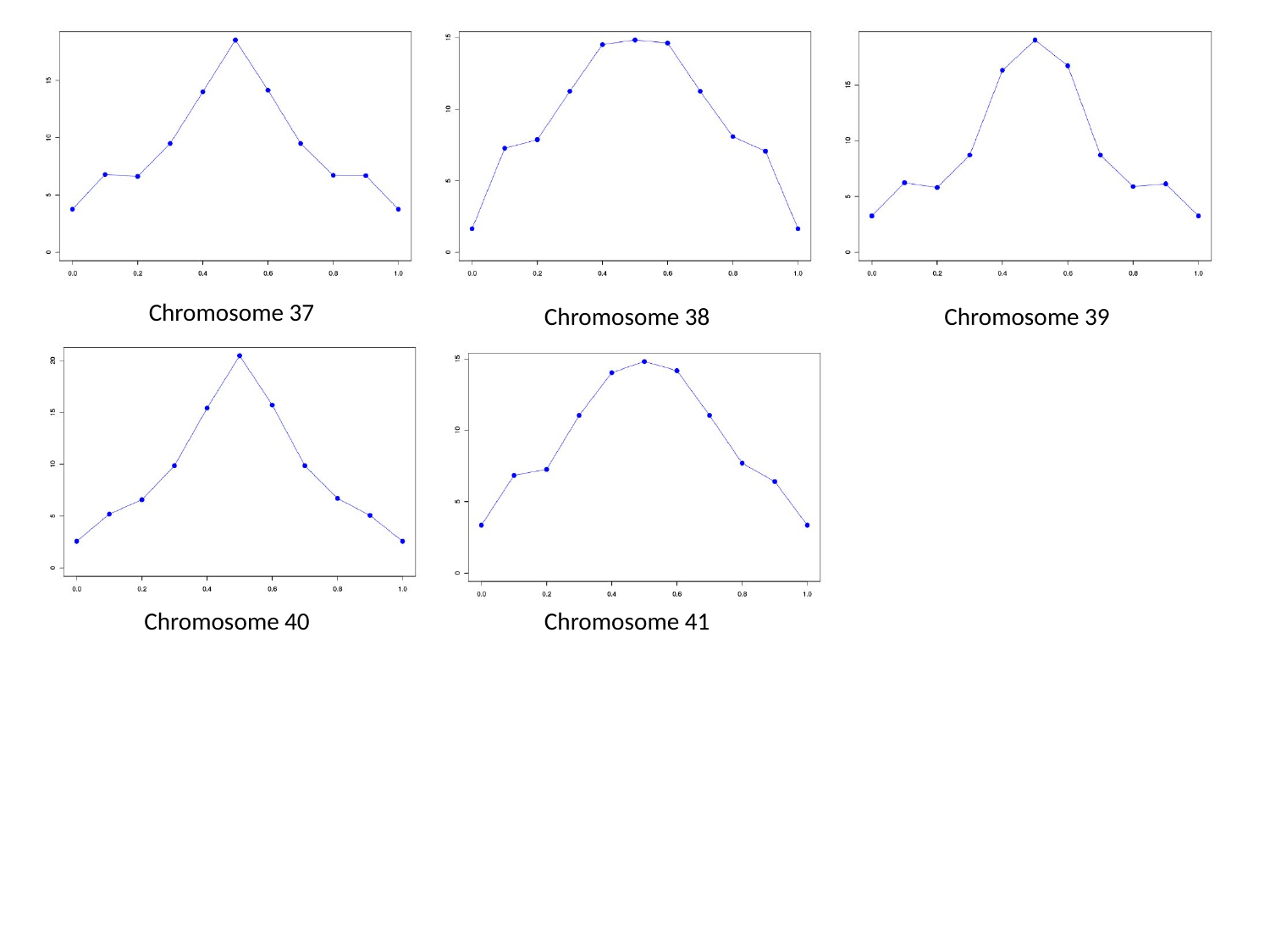

Chromosome 37
Chromosome 39
Chromosome 38
Chromosome 41
Chromosome 40

## Slide 8
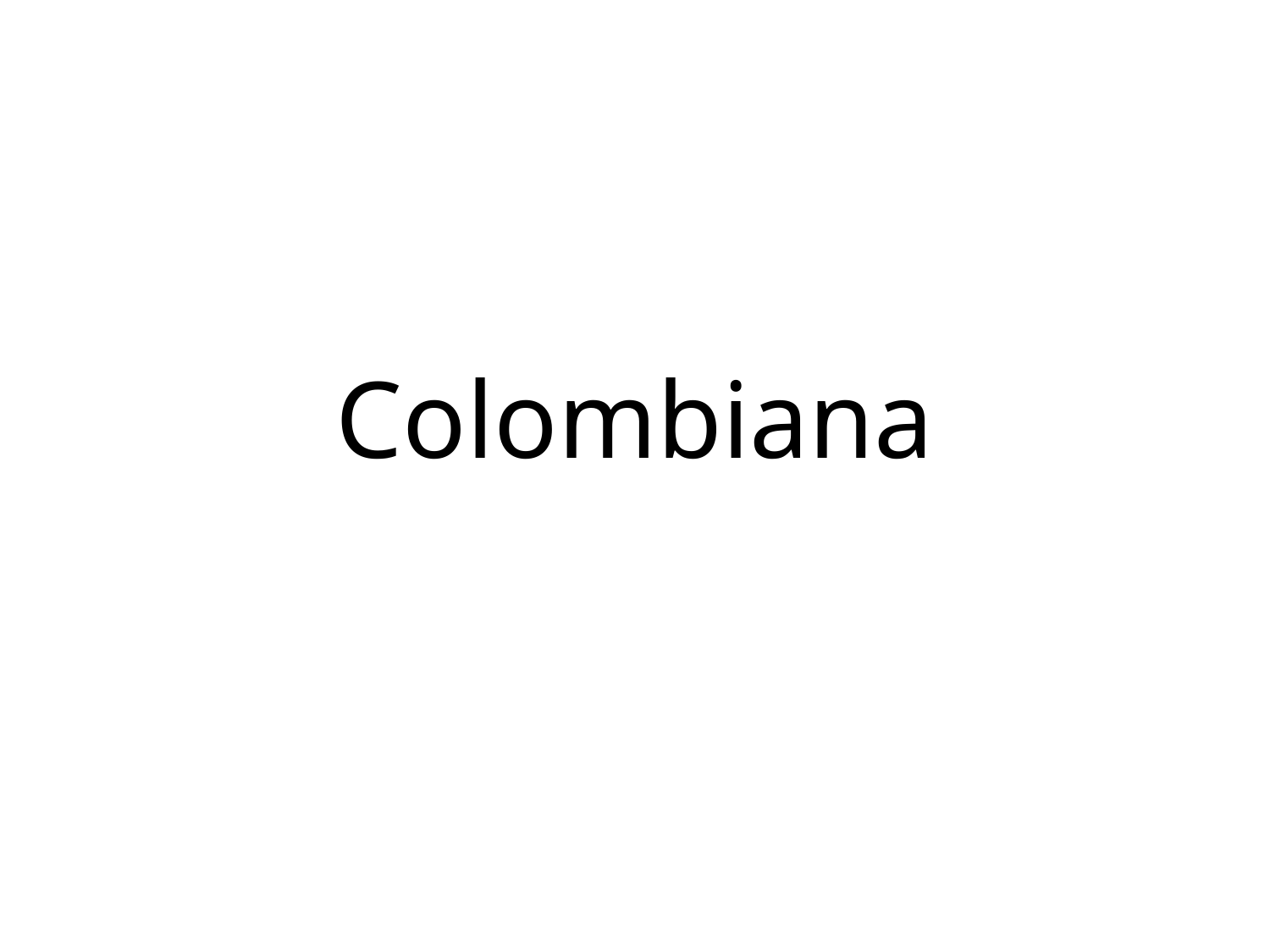

# Colombiana

## Slide 9
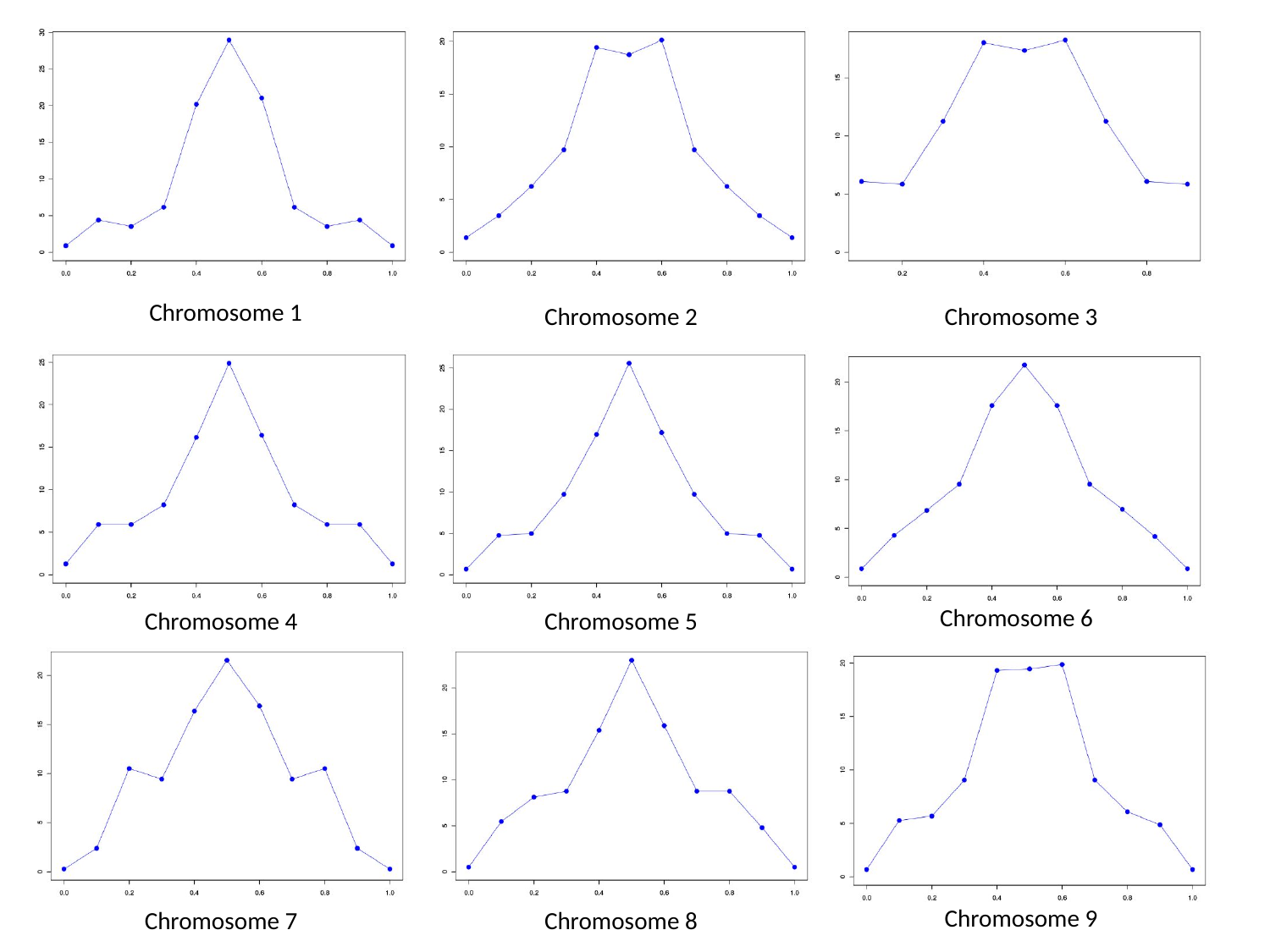

Chromosome 1
Chromosome 3
Chromosome 2
Chromosome 6
Chromosome 5
Chromosome 4
Chromosome 9
Chromosome 8
Chromosome 7

## Slide 10
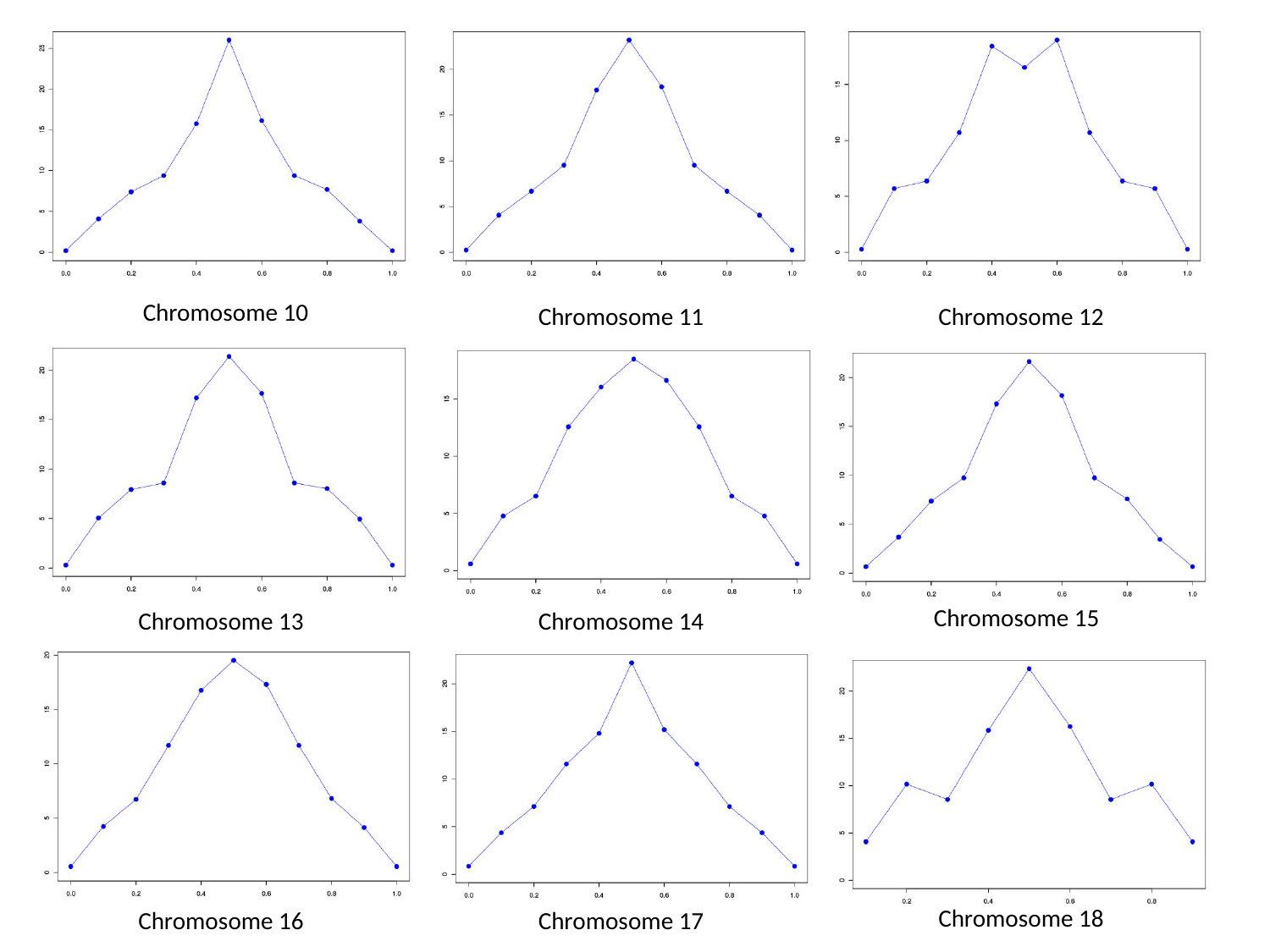

Chromosome 10
Chromosome 12
Chromosome 11
Chromosome 15
Chromosome 14
Chromosome 13
Chromosome 18
Chromosome 17
Chromosome 16

## Slide 11
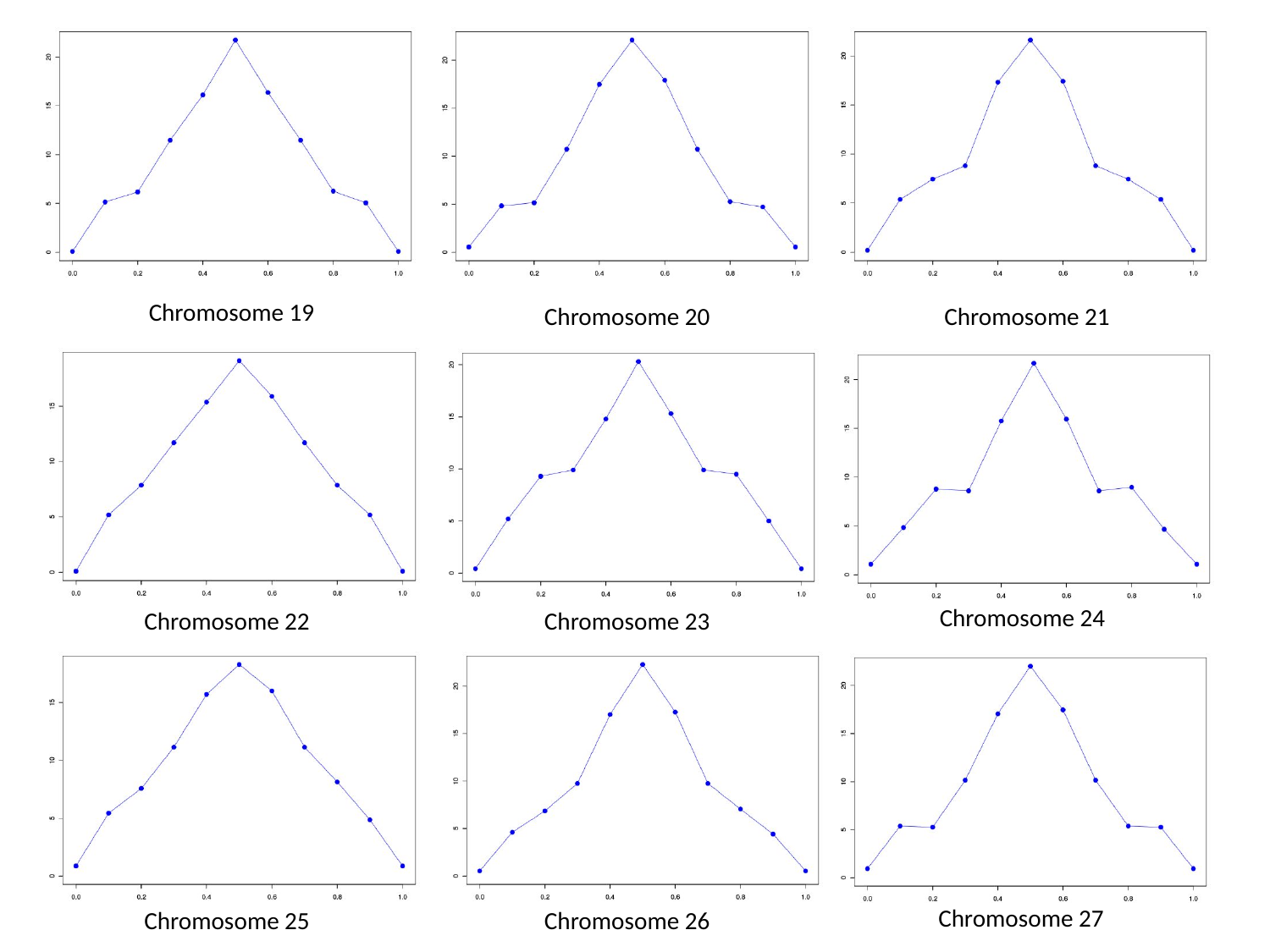

Chromosome 19
Chromosome 21
Chromosome 20
Chromosome 24
Chromosome 23
Chromosome 22
Chromosome 27
Chromosome 26
Chromosome 25

## Slide 12
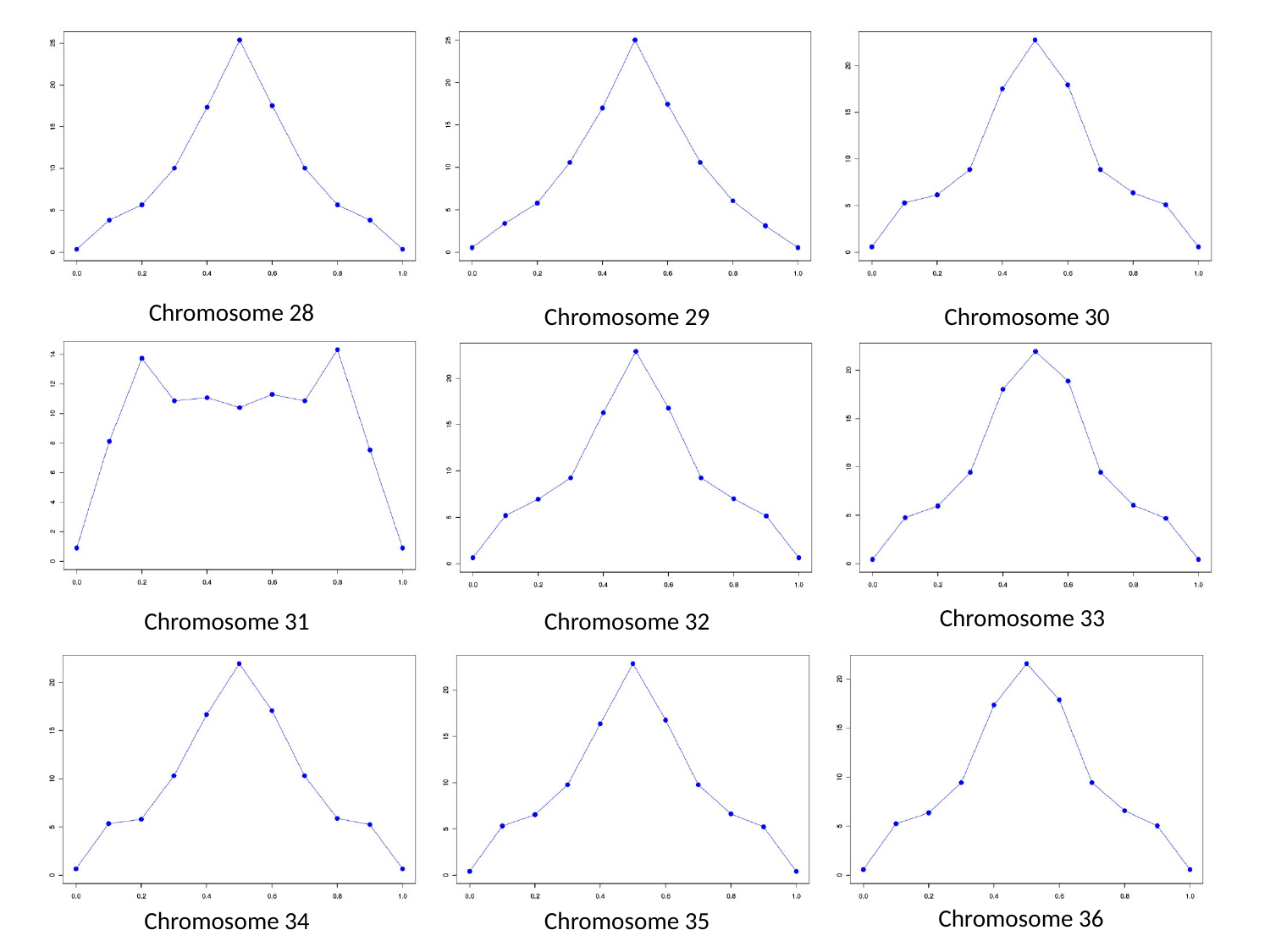

Chromosome 28
Chromosome 30
Chromosome 29
Chromosome 33
Chromosome 32
Chromosome 31
Chromosome 36
Chromosome 35
Chromosome 34

## Slide 13
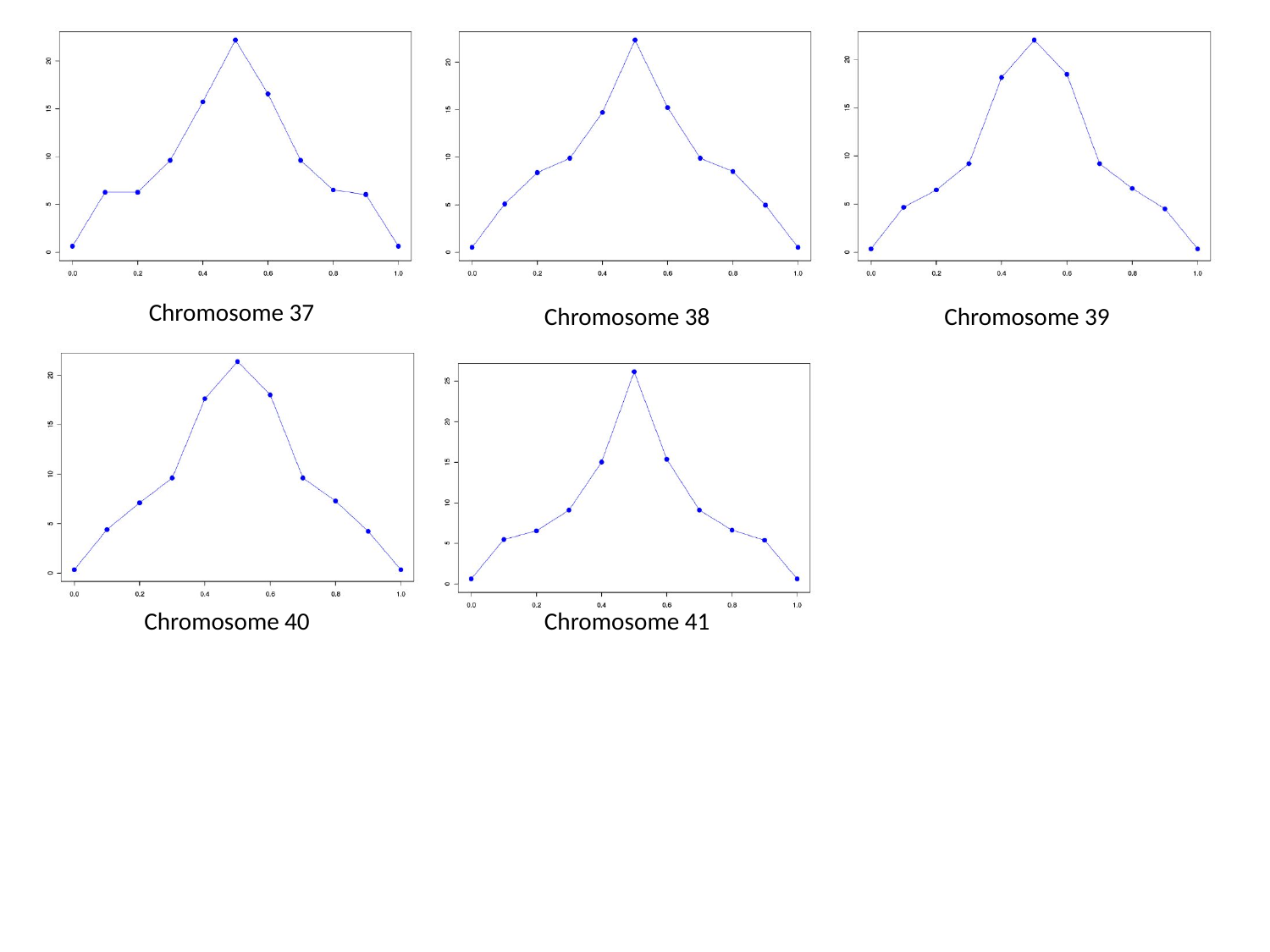

Chromosome 37
Chromosome 39
Chromosome 38
Chromosome 41
Chromosome 40

## Slide 14
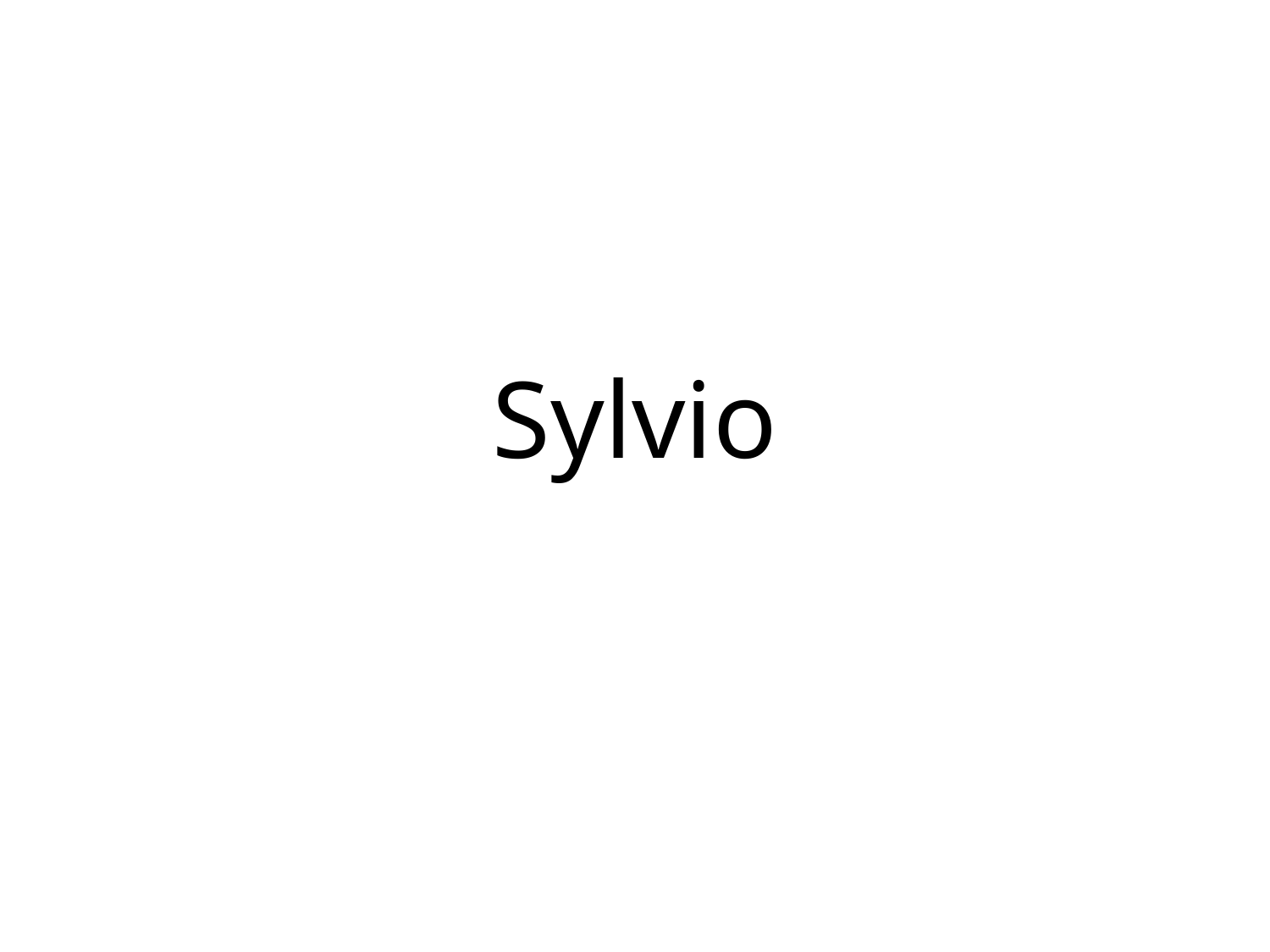

# Sylvio

## Slide 15
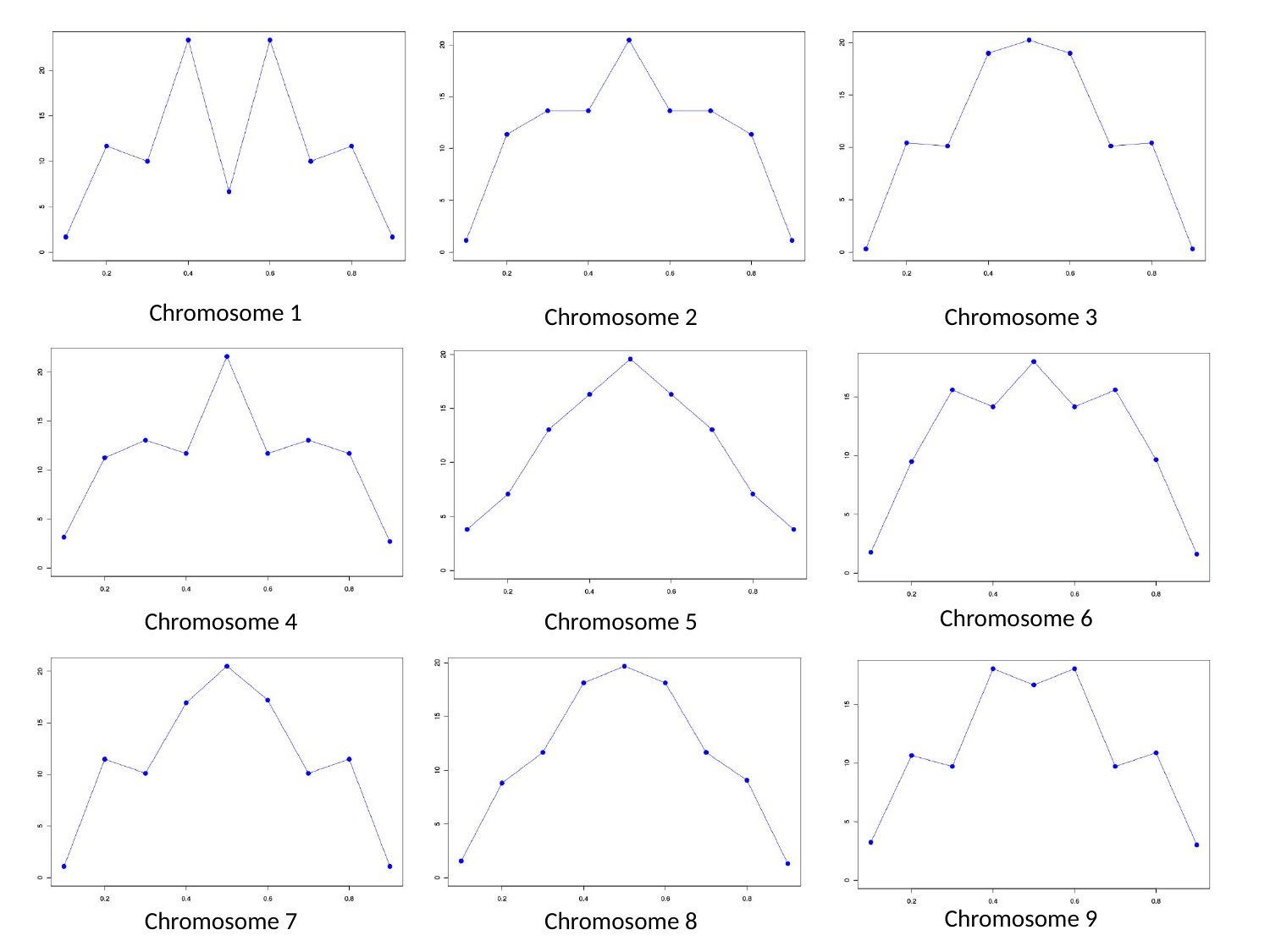

Chromosome 1
Chromosome 3
Chromosome 2
Chromosome 6
Chromosome 5
Chromosome 4
Chromosome 9
Chromosome 8
Chromosome 7

## Slide 16
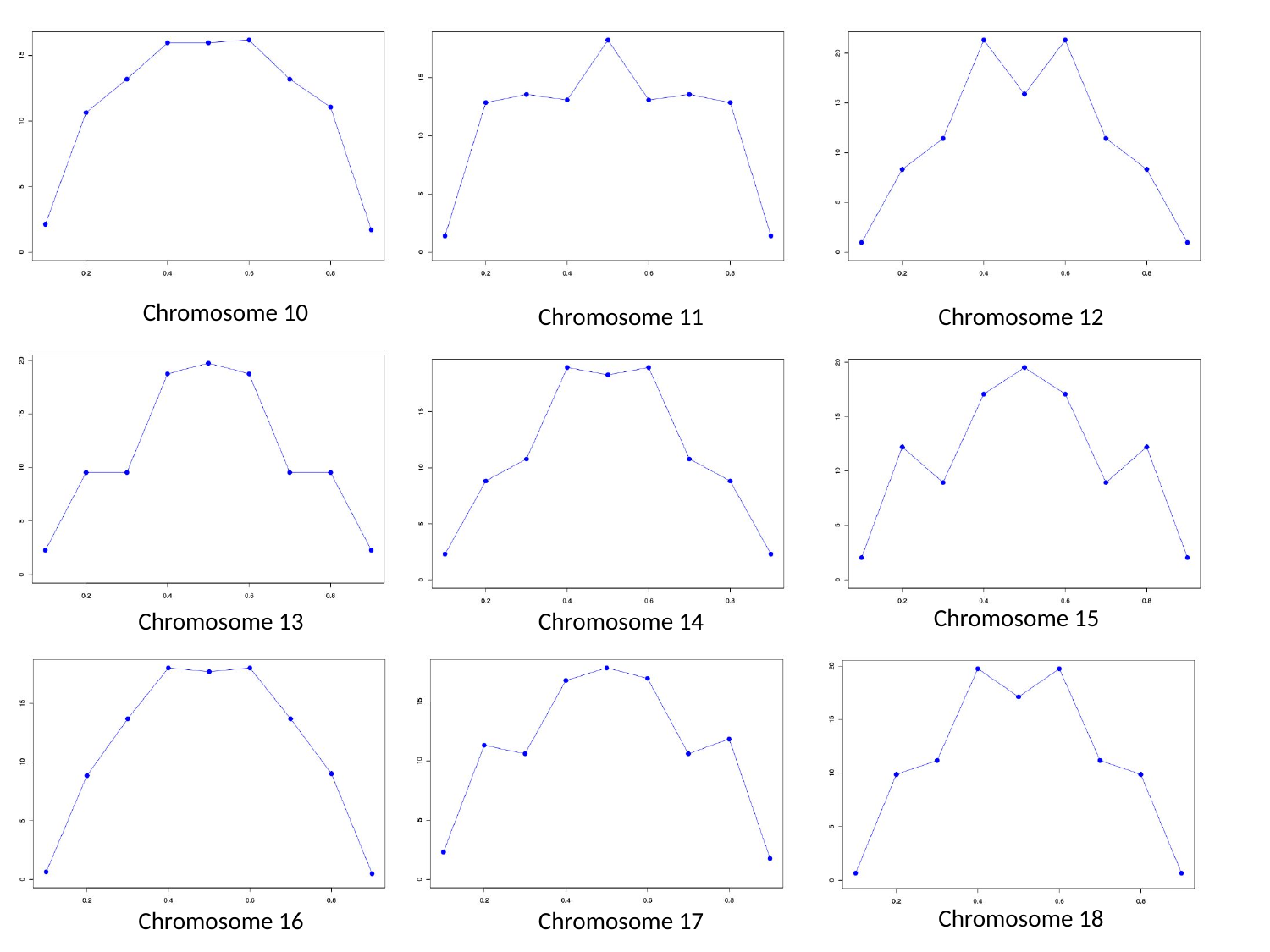

Chromosome 10
Chromosome 12
Chromosome 11
Chromosome 15
Chromosome 14
Chromosome 13
Chromosome 18
Chromosome 17
Chromosome 16

## Slide 17
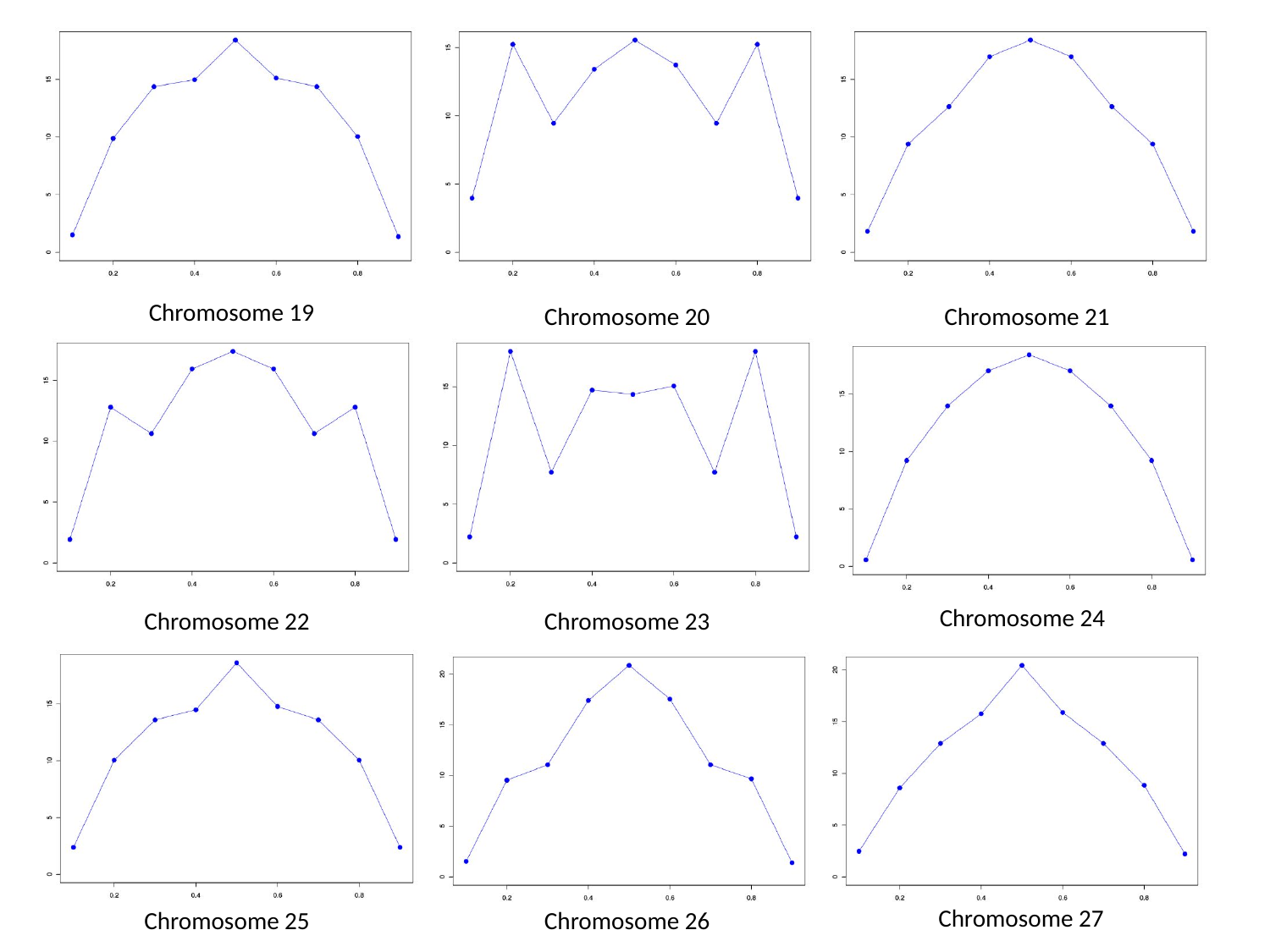

Chromosome 19
Chromosome 21
Chromosome 20
Chromosome 24
Chromosome 23
Chromosome 22
Chromosome 27
Chromosome 26
Chromosome 25

## Slide 18
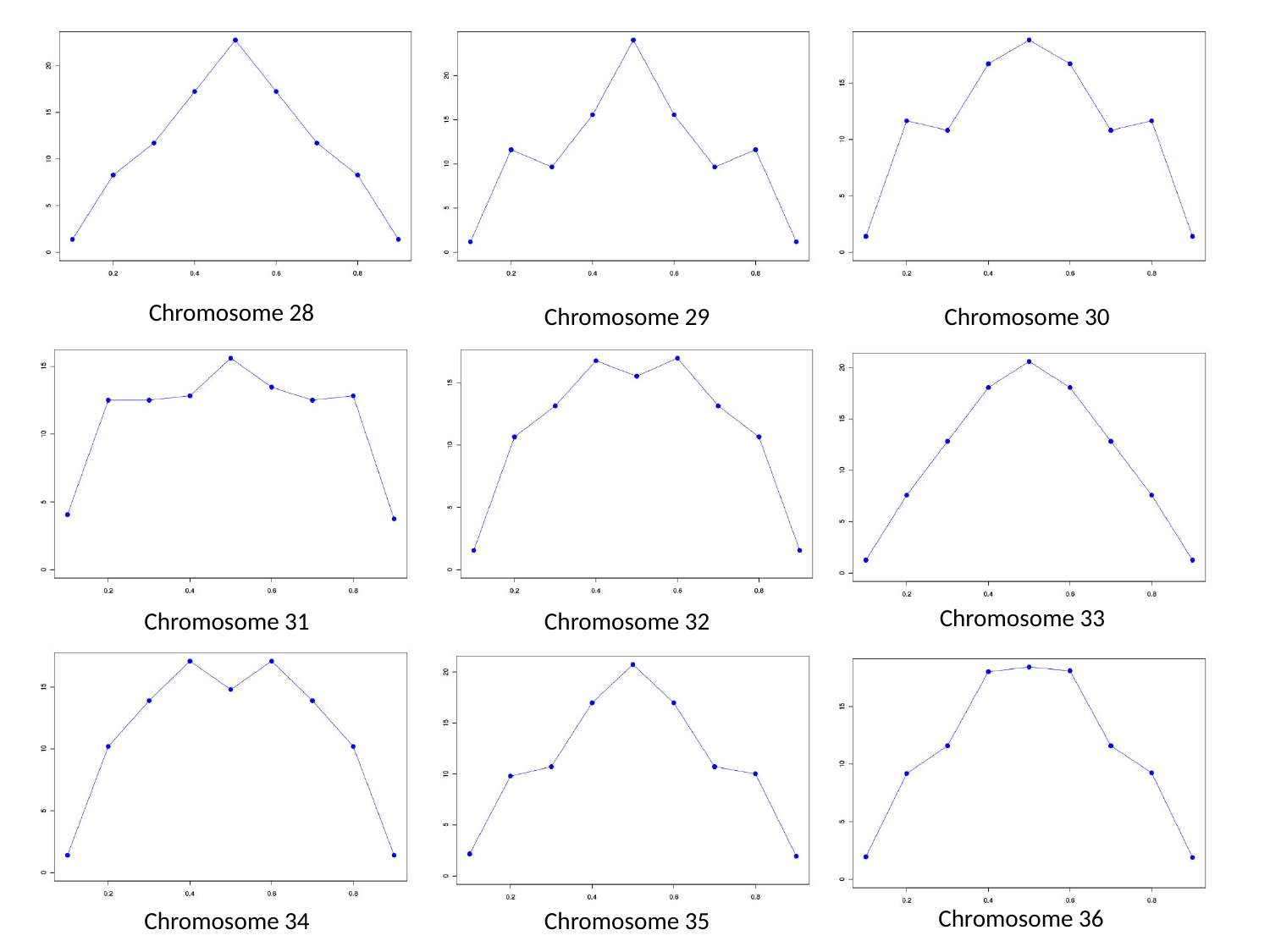

Chromosome 28
Chromosome 30
Chromosome 29
Chromosome 33
Chromosome 32
Chromosome 31
Chromosome 36
Chromosome 35
Chromosome 34

## Slide 19
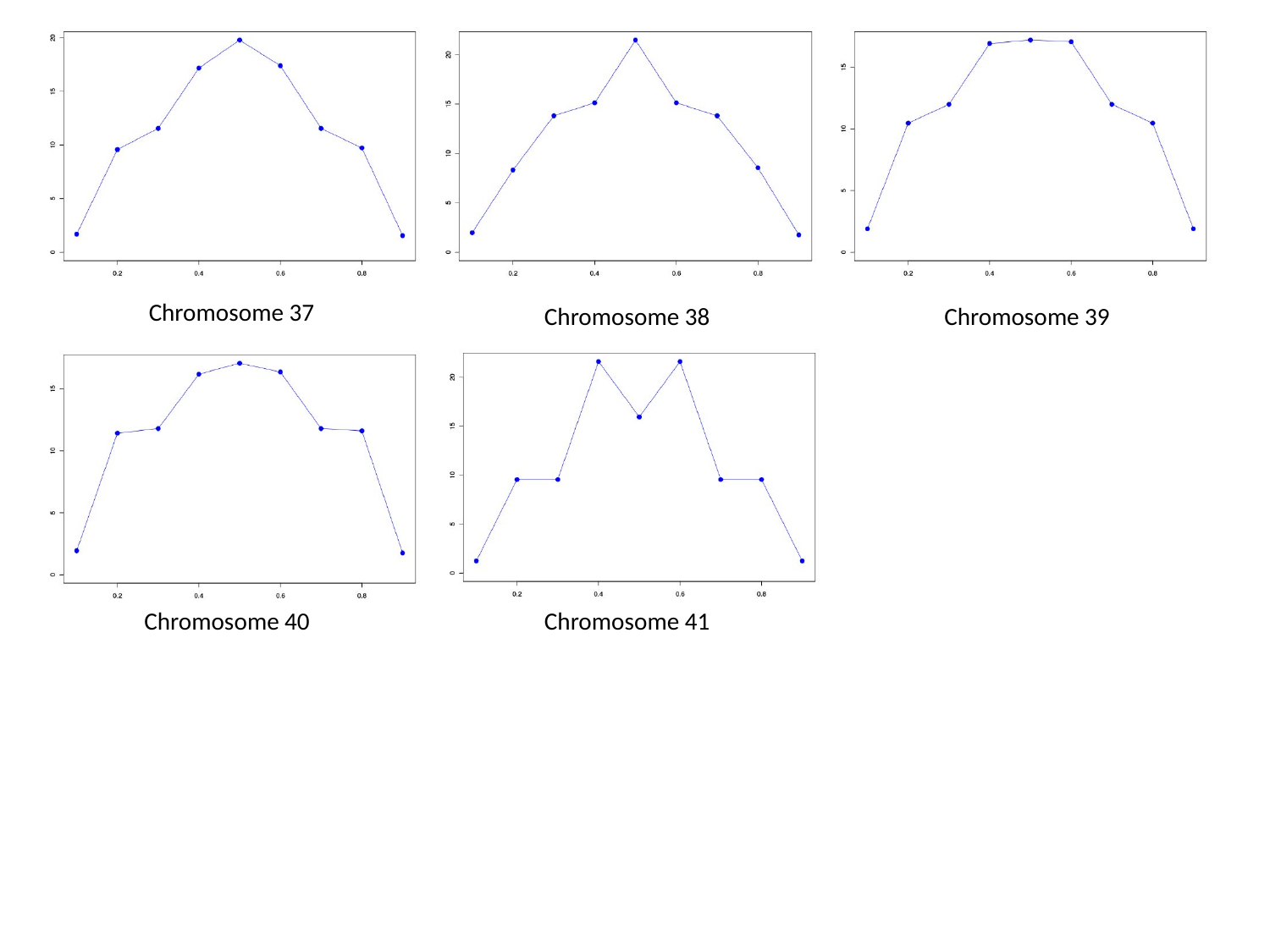

Chromosome 37
Chromosome 39
Chromosome 38
Chromosome 41
Chromosome 40

## Slide 20
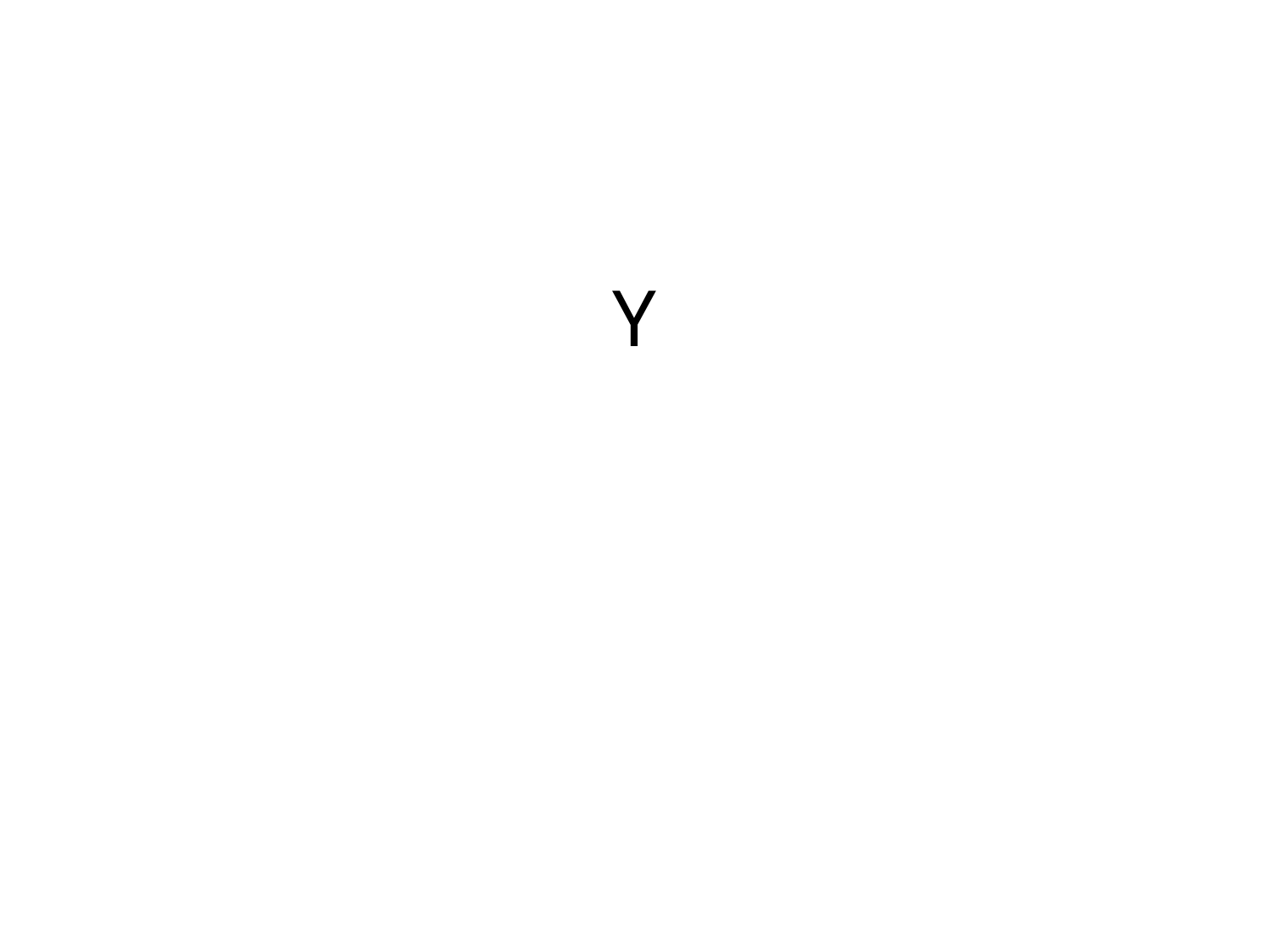

Y

## Slide 21
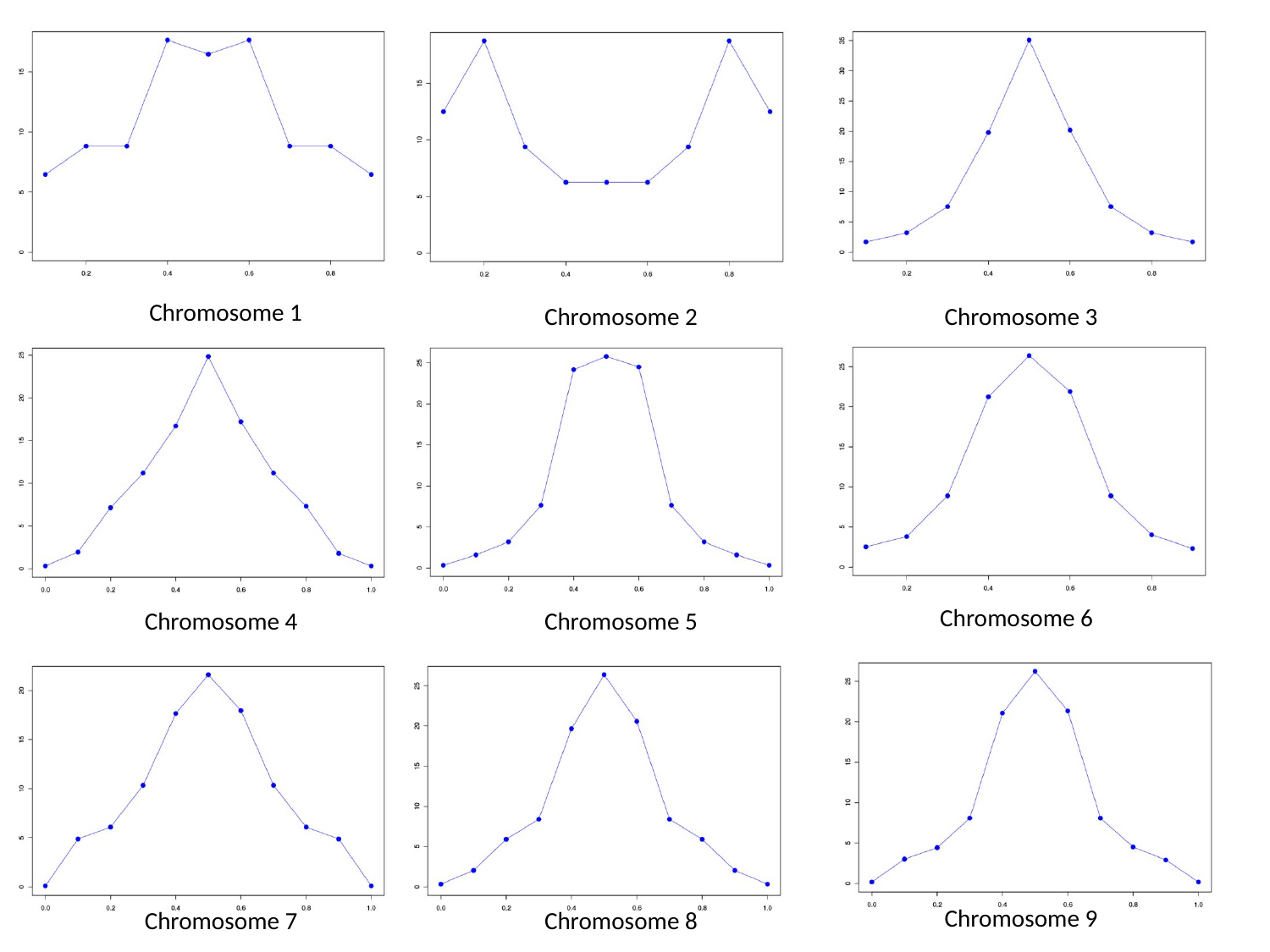

Chromosome 1
Chromosome 3
Chromosome 2
Chromosome 6
Chromosome 5
Chromosome 4
Chromosome 9
Chromosome 8
Chromosome 7

## Slide 22
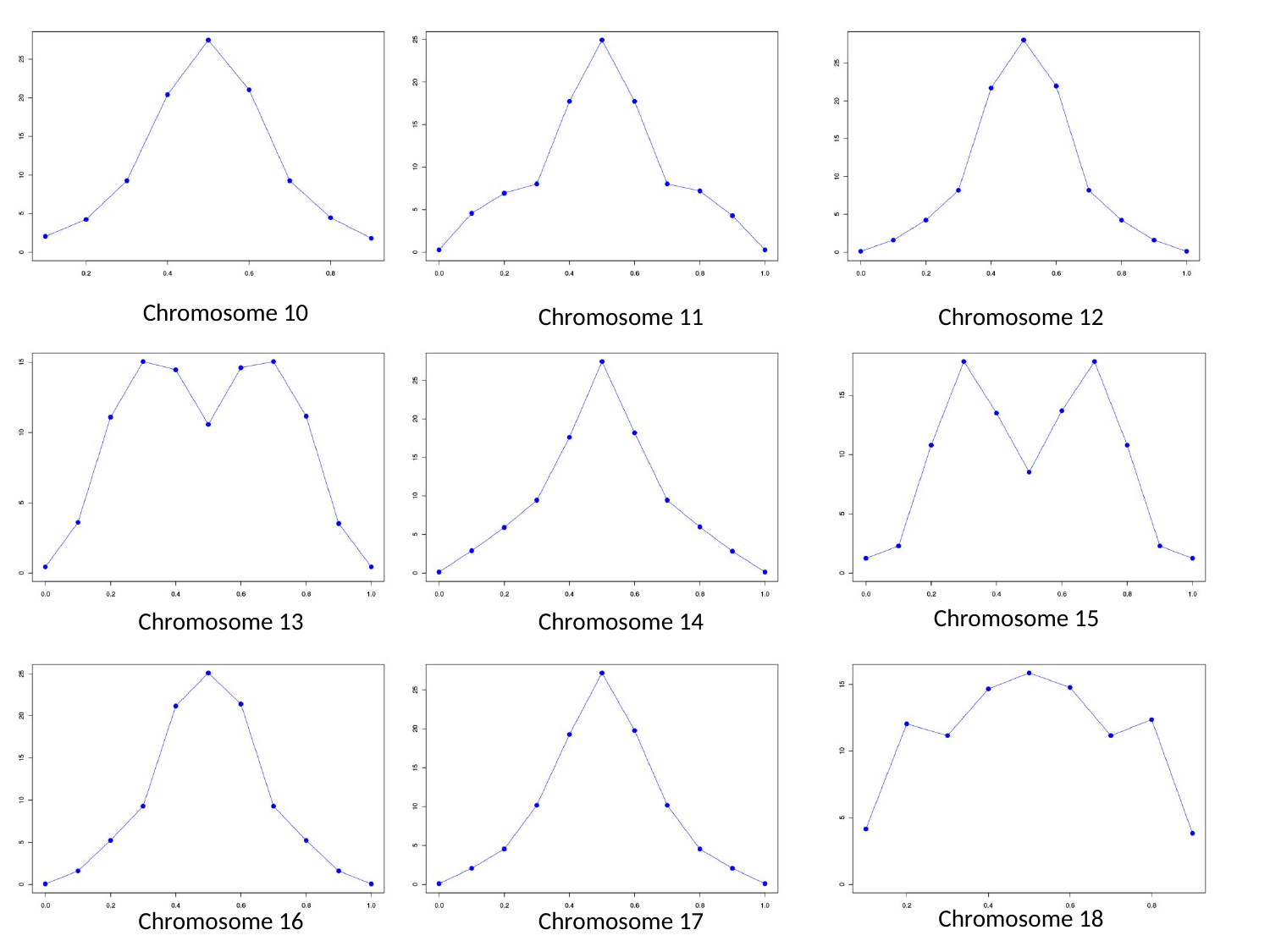

Chromosome 10
Chromosome 12
Chromosome 11
Chromosome 15
Chromosome 14
Chromosome 13
Chromosome 18
Chromosome 17
Chromosome 16

## Slide 23
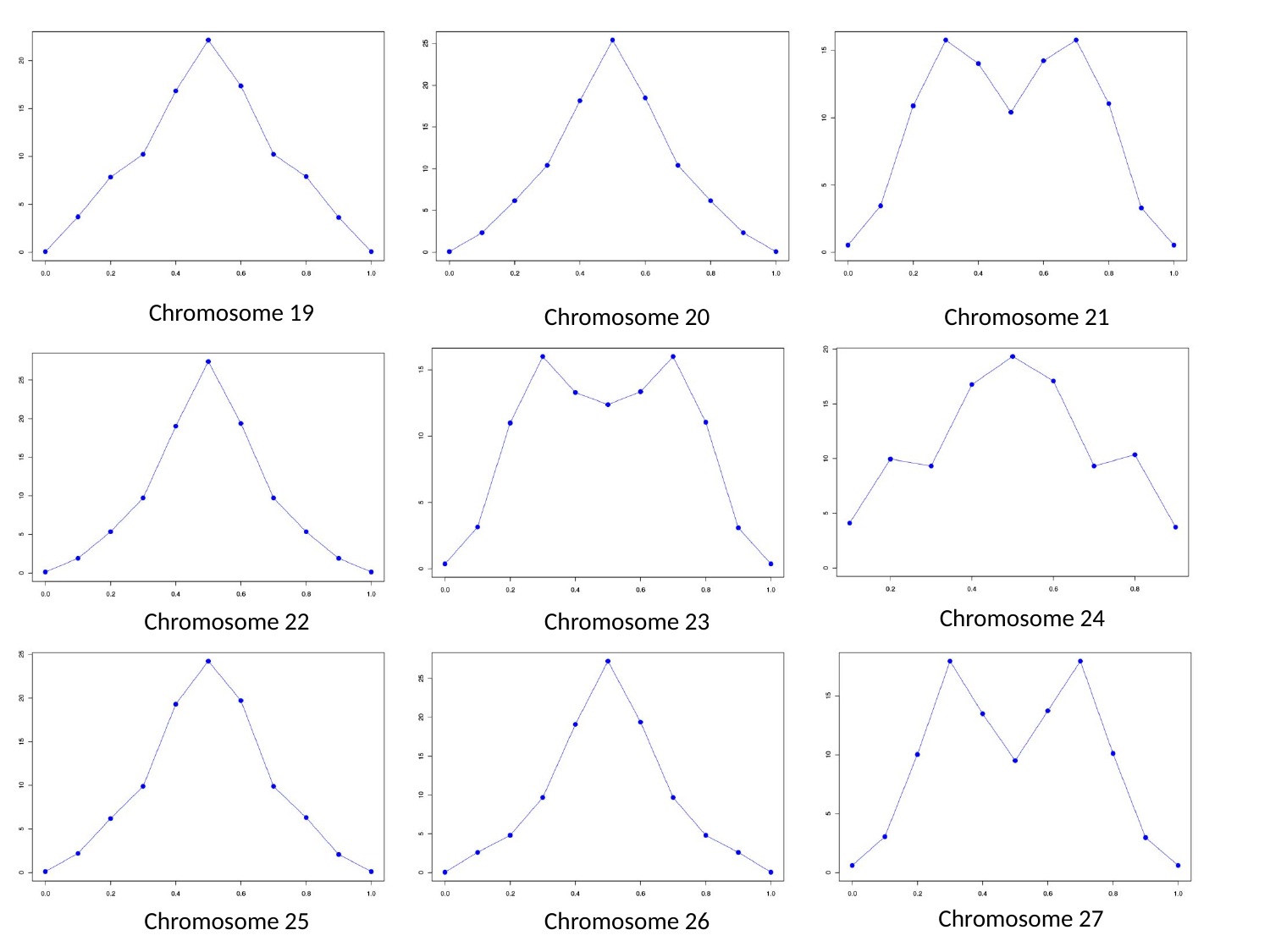

Chromosome 19
Chromosome 21
Chromosome 20
Chromosome 24
Chromosome 23
Chromosome 22
Chromosome 27
Chromosome 26
Chromosome 25

## Slide 24
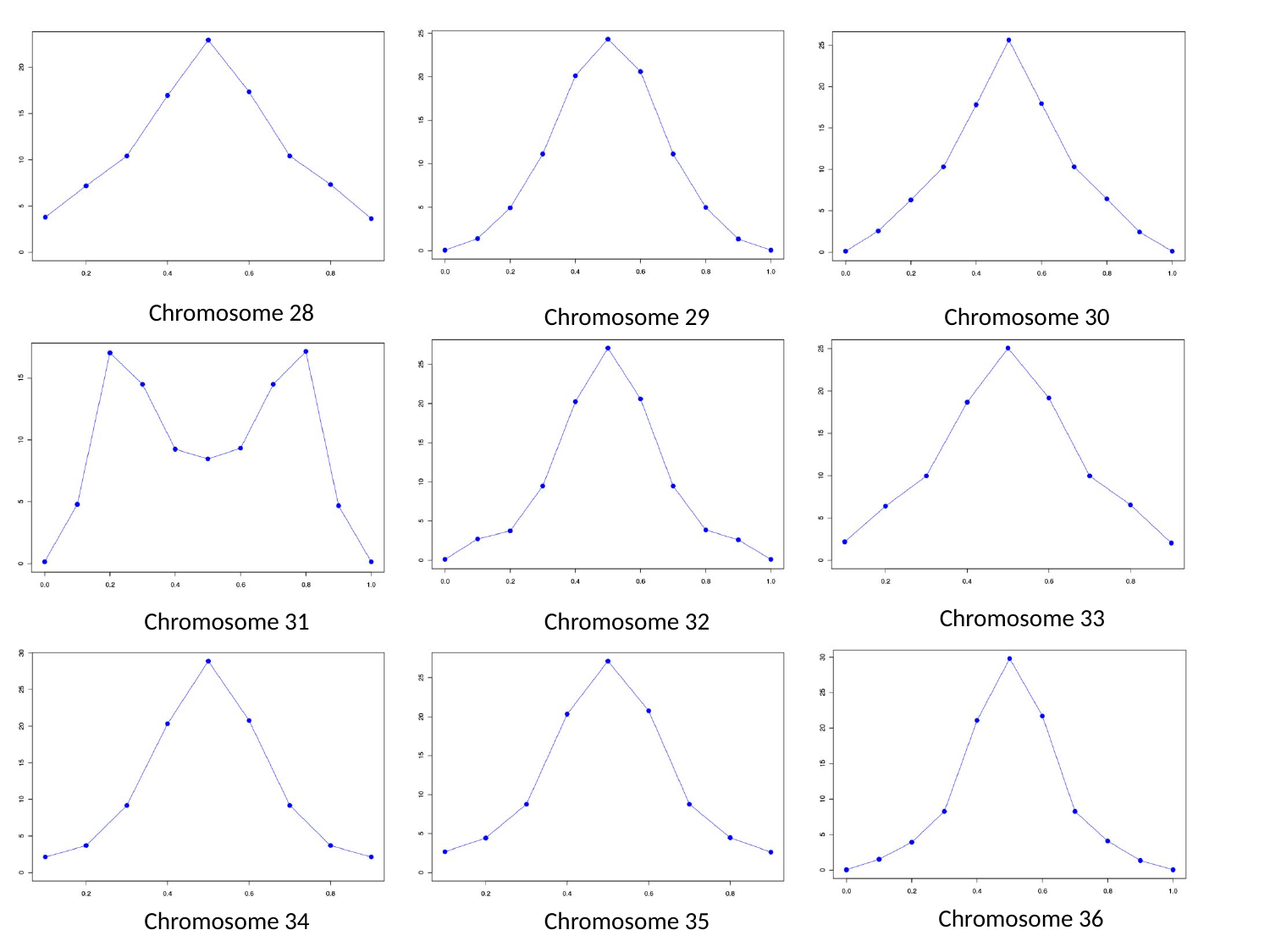

Chromosome 28
Chromosome 30
Chromosome 29
Chromosome 33
Chromosome 32
Chromosome 31
Chromosome 36
Chromosome 35
Chromosome 34

## Slide 25
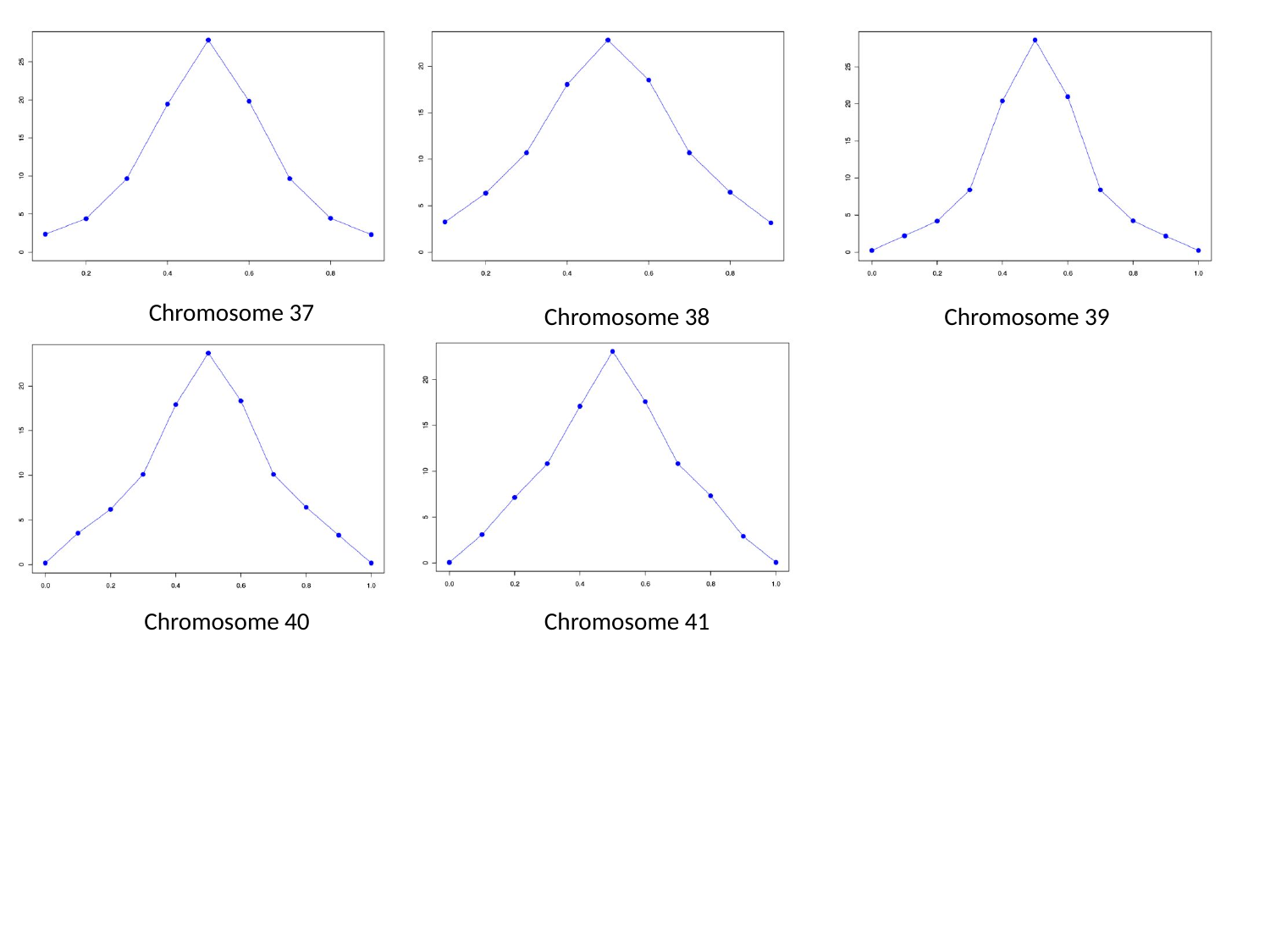

Chromosome 37
Chromosome 39
Chromosome 38
Chromosome 41
Chromosome 40

## Slide 26
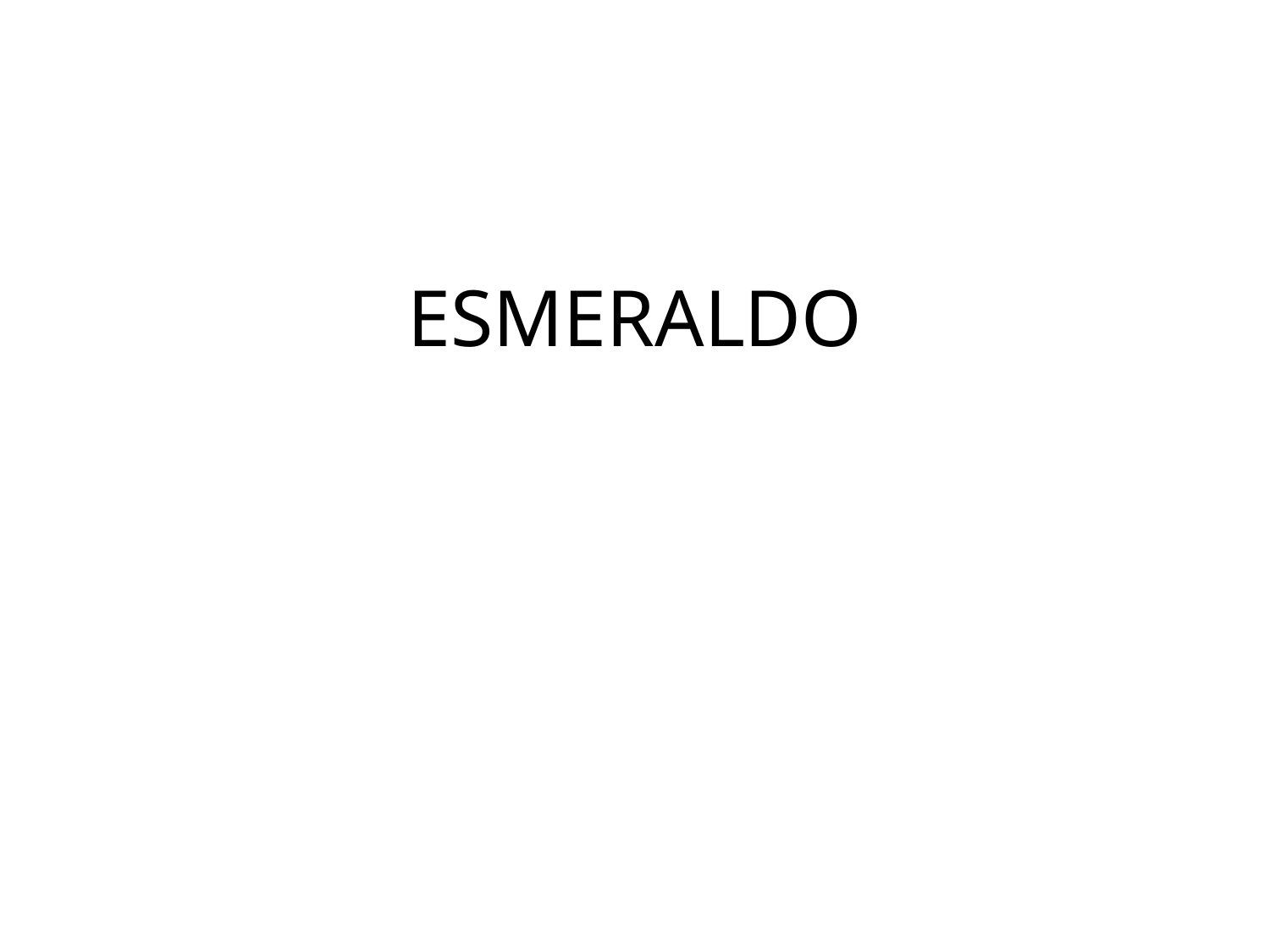

ESMERALDO

## Slide 27
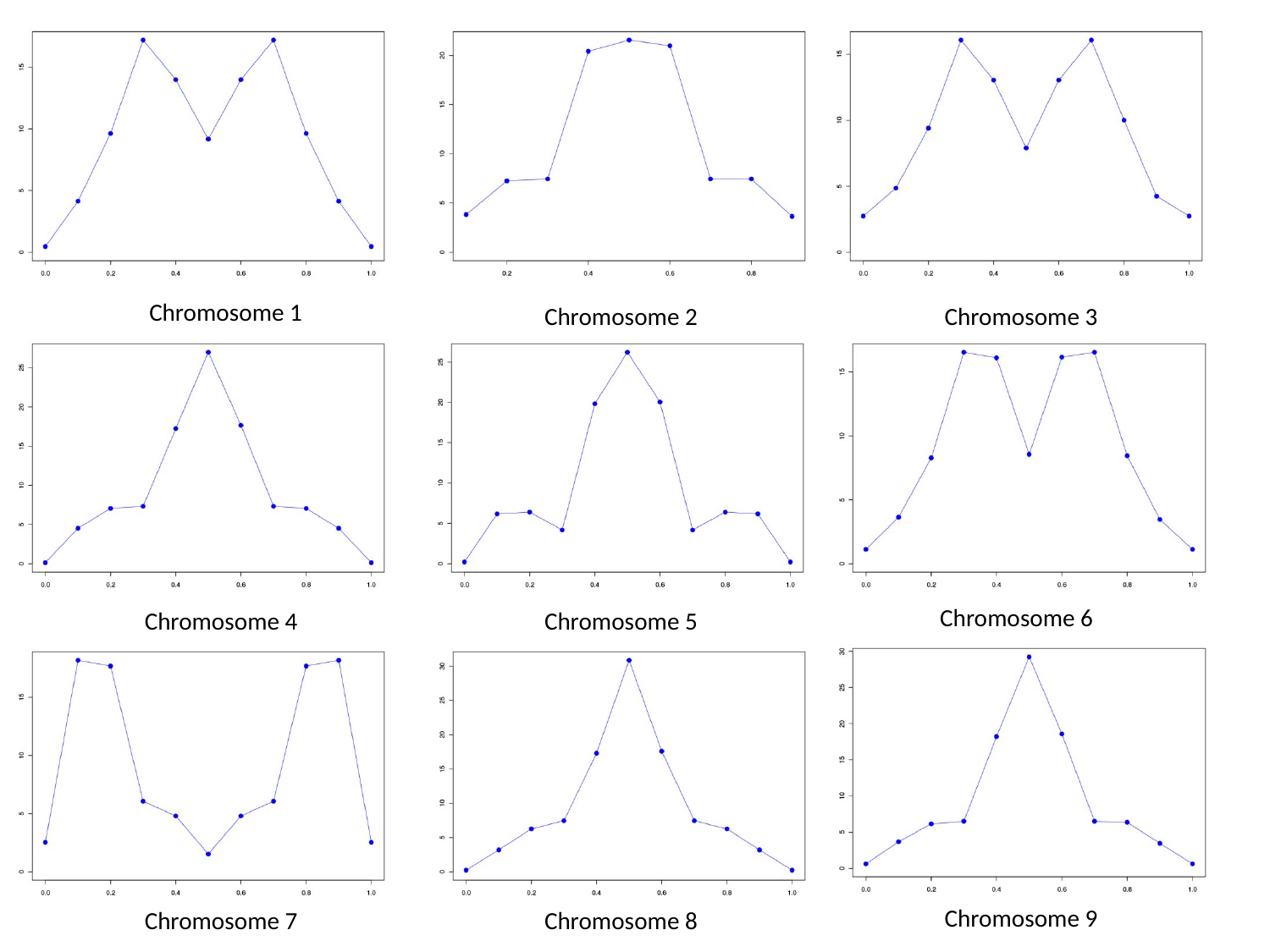

Chromosome 1
Chromosome 3
Chromosome 2
Chromosome 6
Chromosome 5
Chromosome 4
Chromosome 9
Chromosome 8
Chromosome 7

## Slide 28
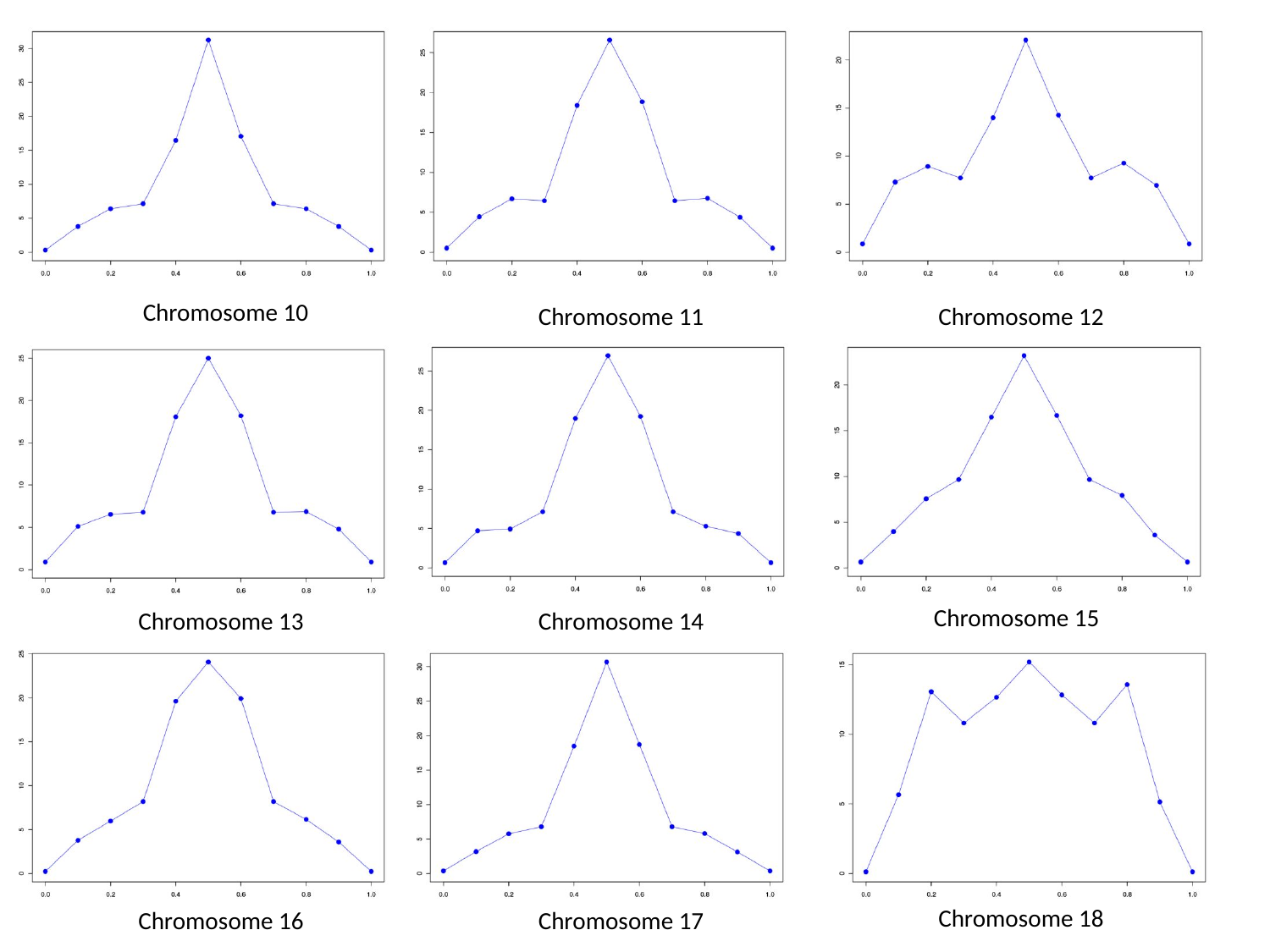

Chromosome 10
Chromosome 12
Chromosome 11
Chromosome 15
Chromosome 14
Chromosome 13
Chromosome 18
Chromosome 17
Chromosome 16

## Slide 29
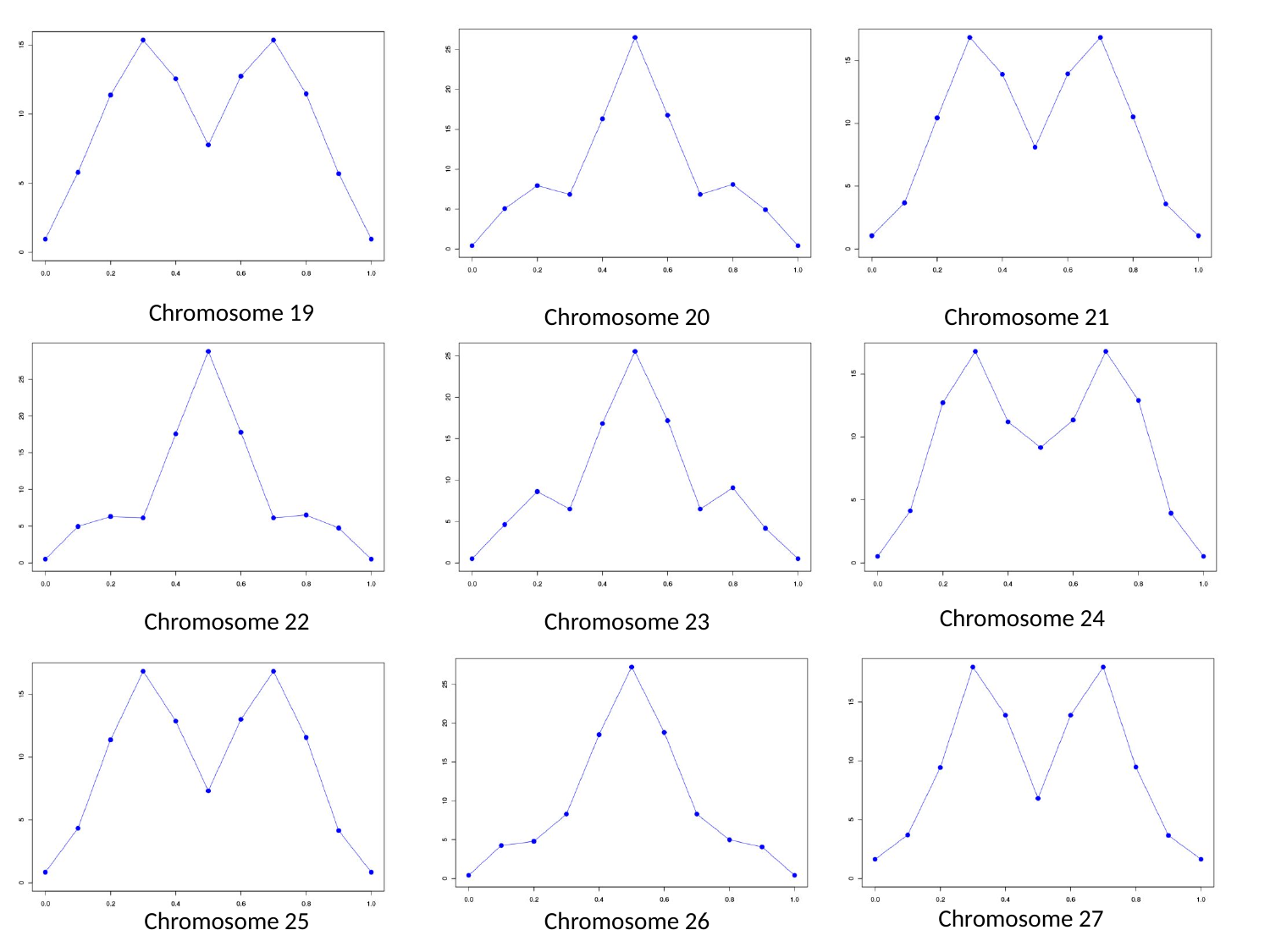

Chromosome 19
Chromosome 21
Chromosome 20
Chromosome 24
Chromosome 23
Chromosome 22
Chromosome 27
Chromosome 26
Chromosome 25

## Slide 30
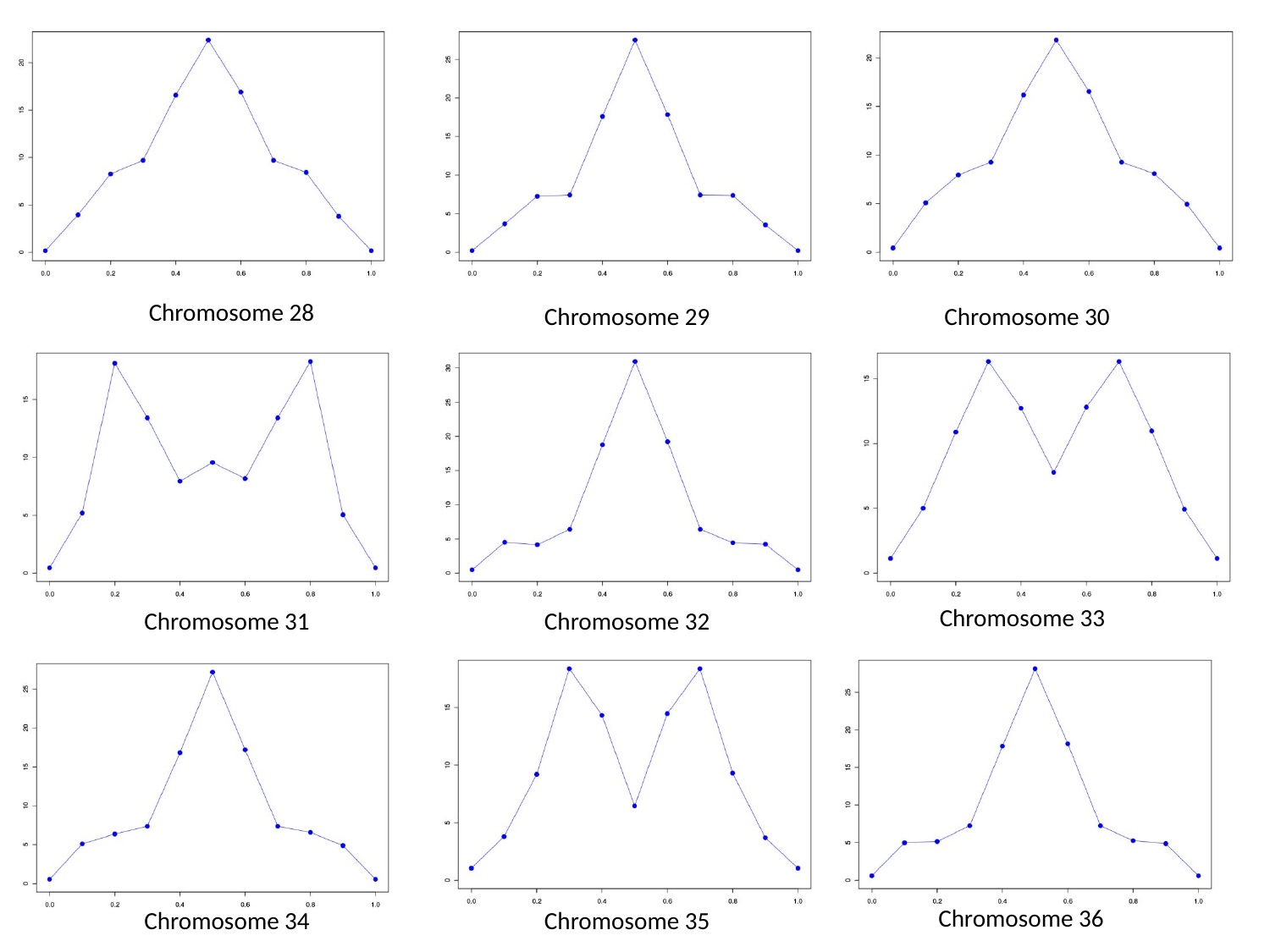

Chromosome 28
Chromosome 30
Chromosome 29
Chromosome 33
Chromosome 32
Chromosome 31
Chromosome 36
Chromosome 35
Chromosome 34

## Slide 31
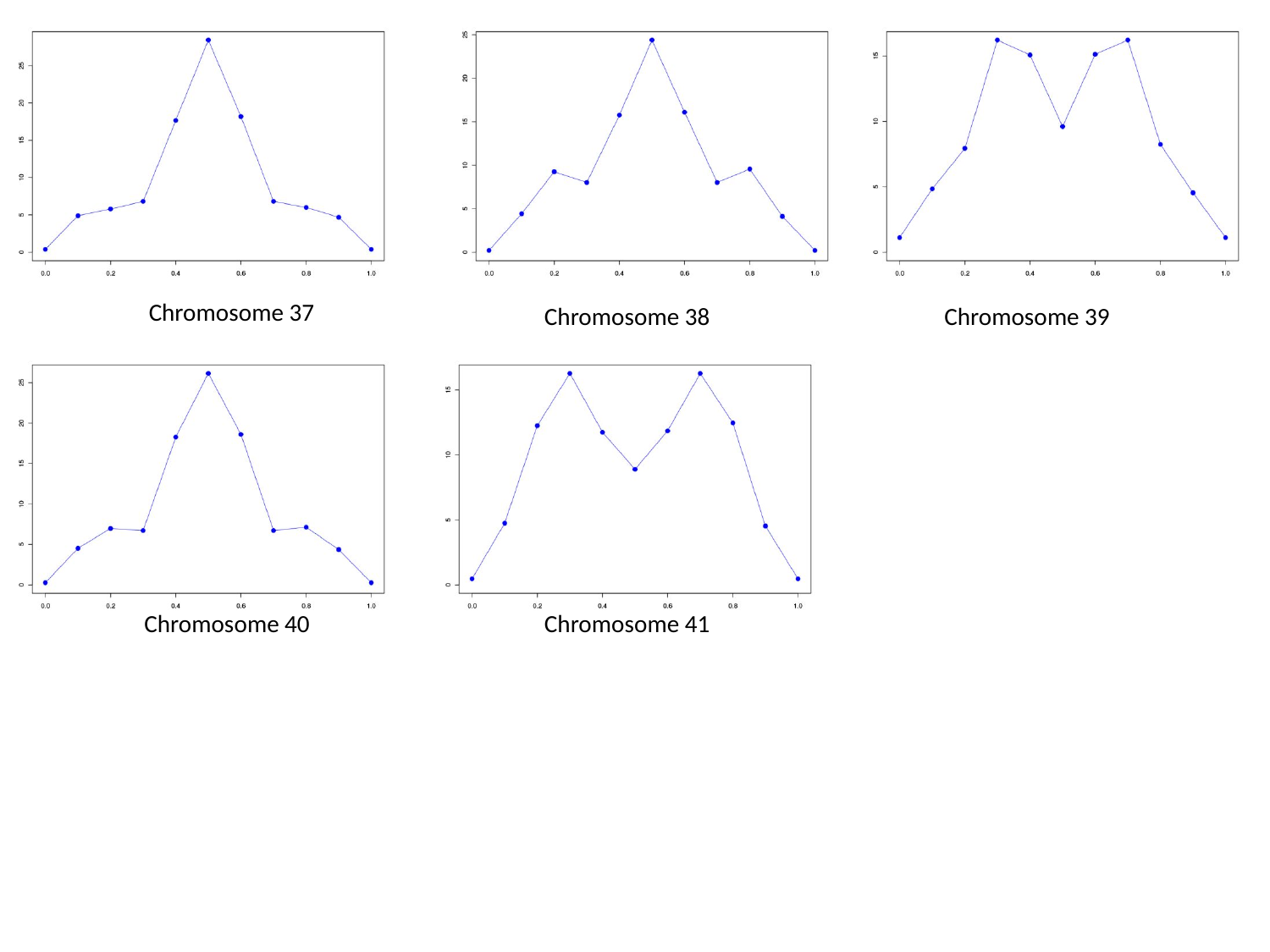

Chromosome 37
Chromosome 39
Chromosome 38
Chromosome 41
Chromosome 40

## Slide 32
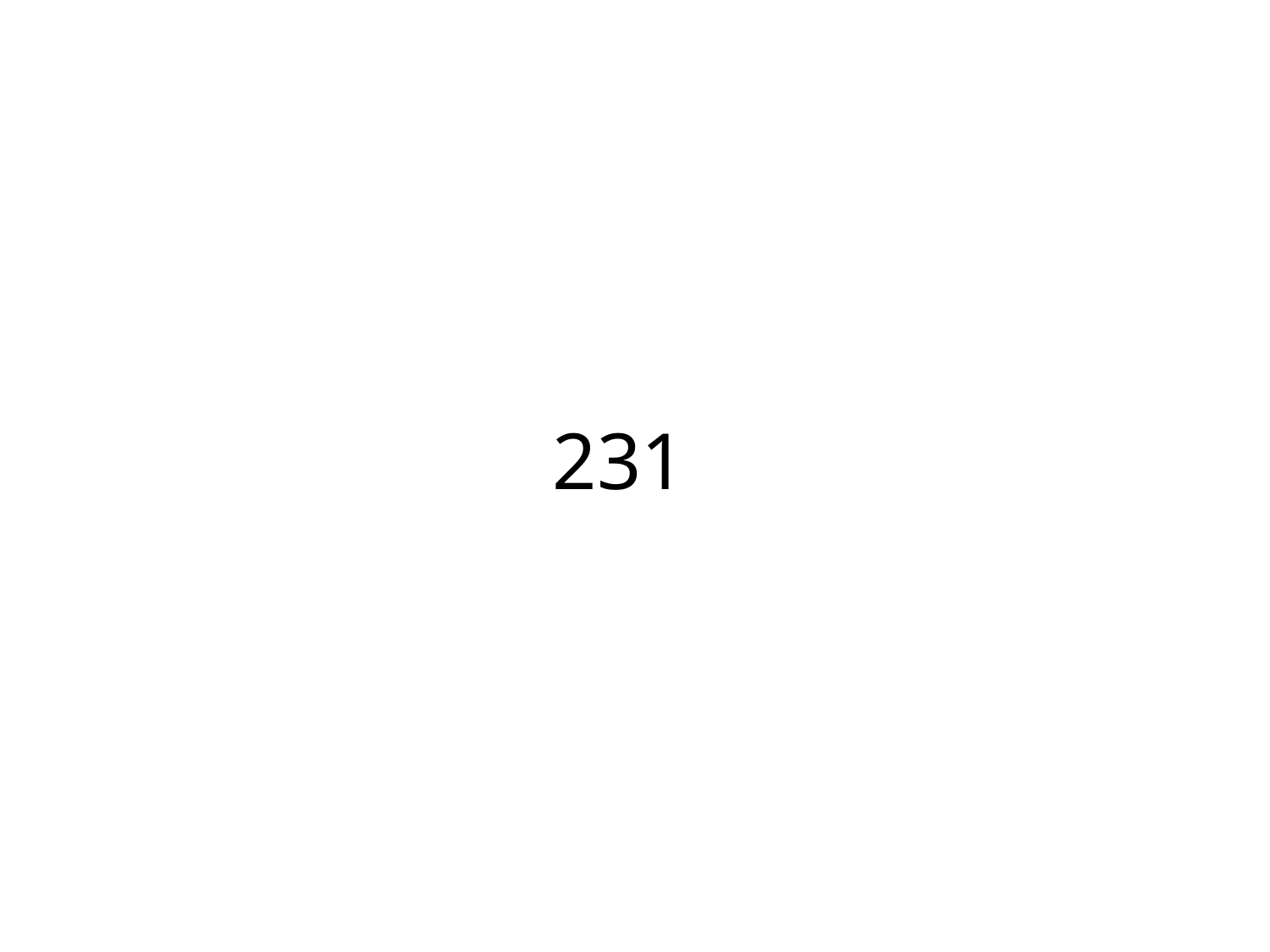

231

## Slide 33
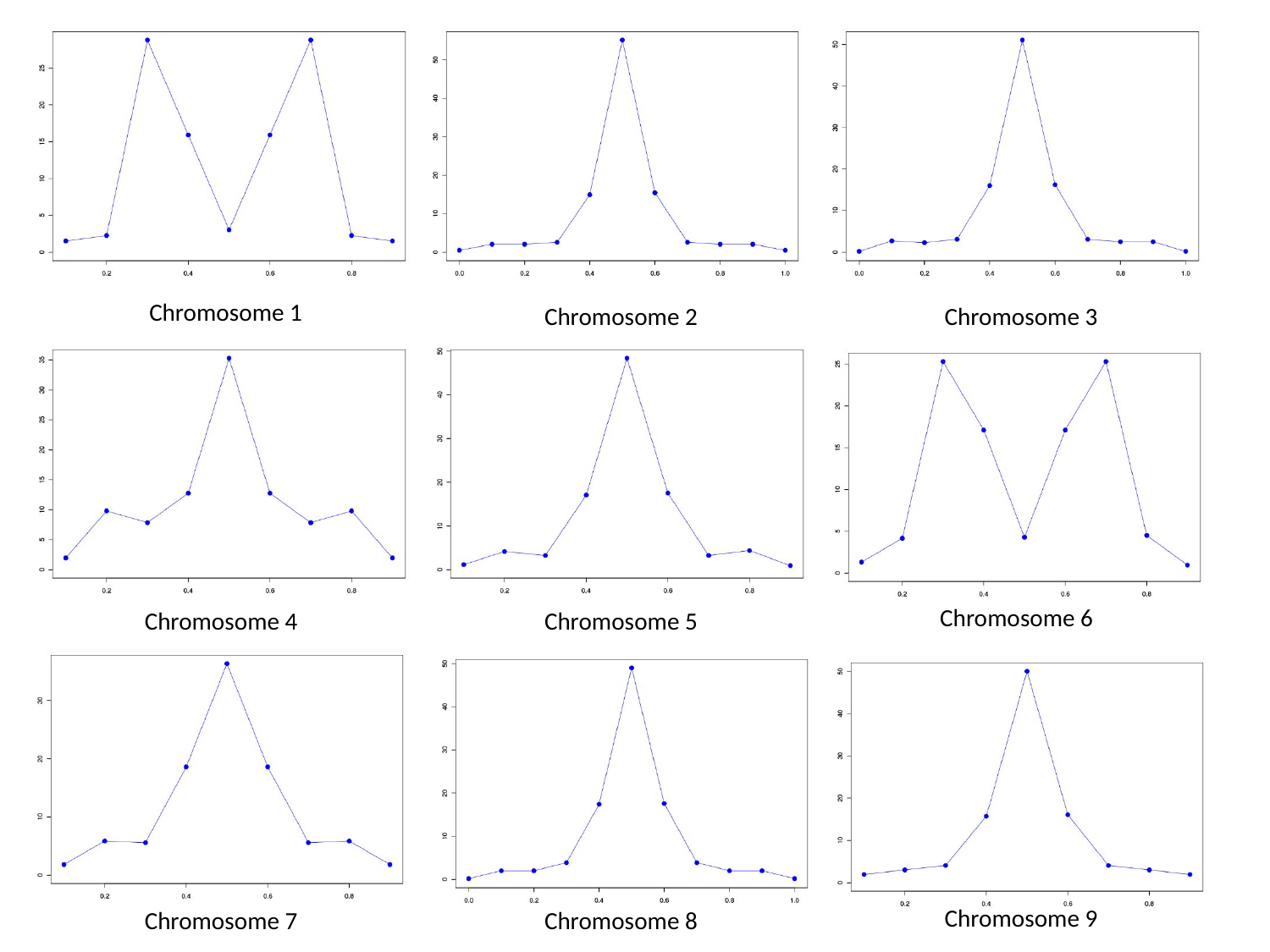

Chromosome 1
Chromosome 3
Chromosome 2
Chromosome 6
Chromosome 5
Chromosome 4
Chromosome 9
Chromosome 8
Chromosome 7

## Slide 34
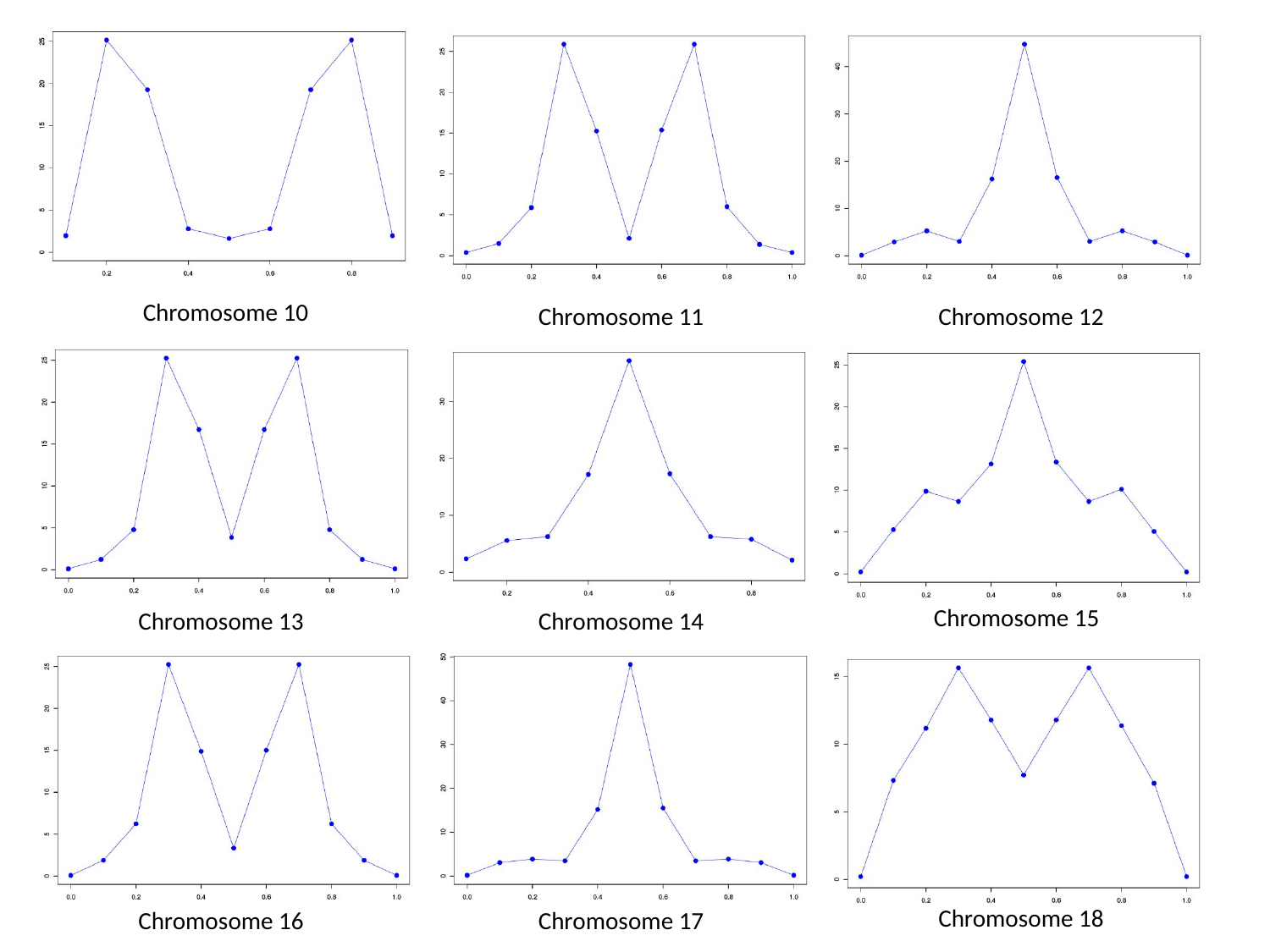

Chromosome 10
Chromosome 12
Chromosome 11
Chromosome 15
Chromosome 14
Chromosome 13
Chromosome 18
Chromosome 17
Chromosome 16

## Slide 35
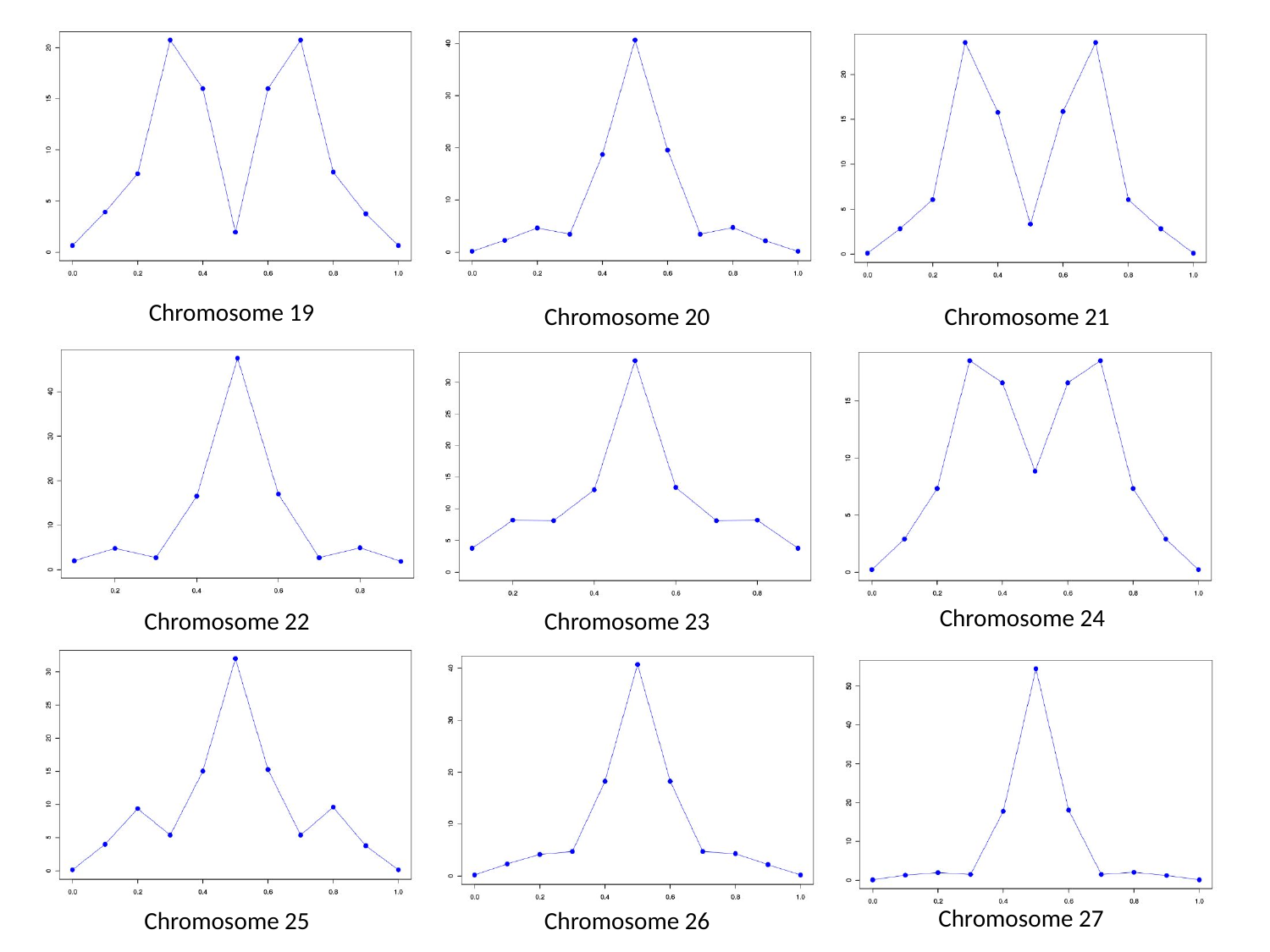

Chromosome 19
Chromosome 21
Chromosome 20
Chromosome 24
Chromosome 23
Chromosome 22
Chromosome 27
Chromosome 26
Chromosome 25

## Slide 36
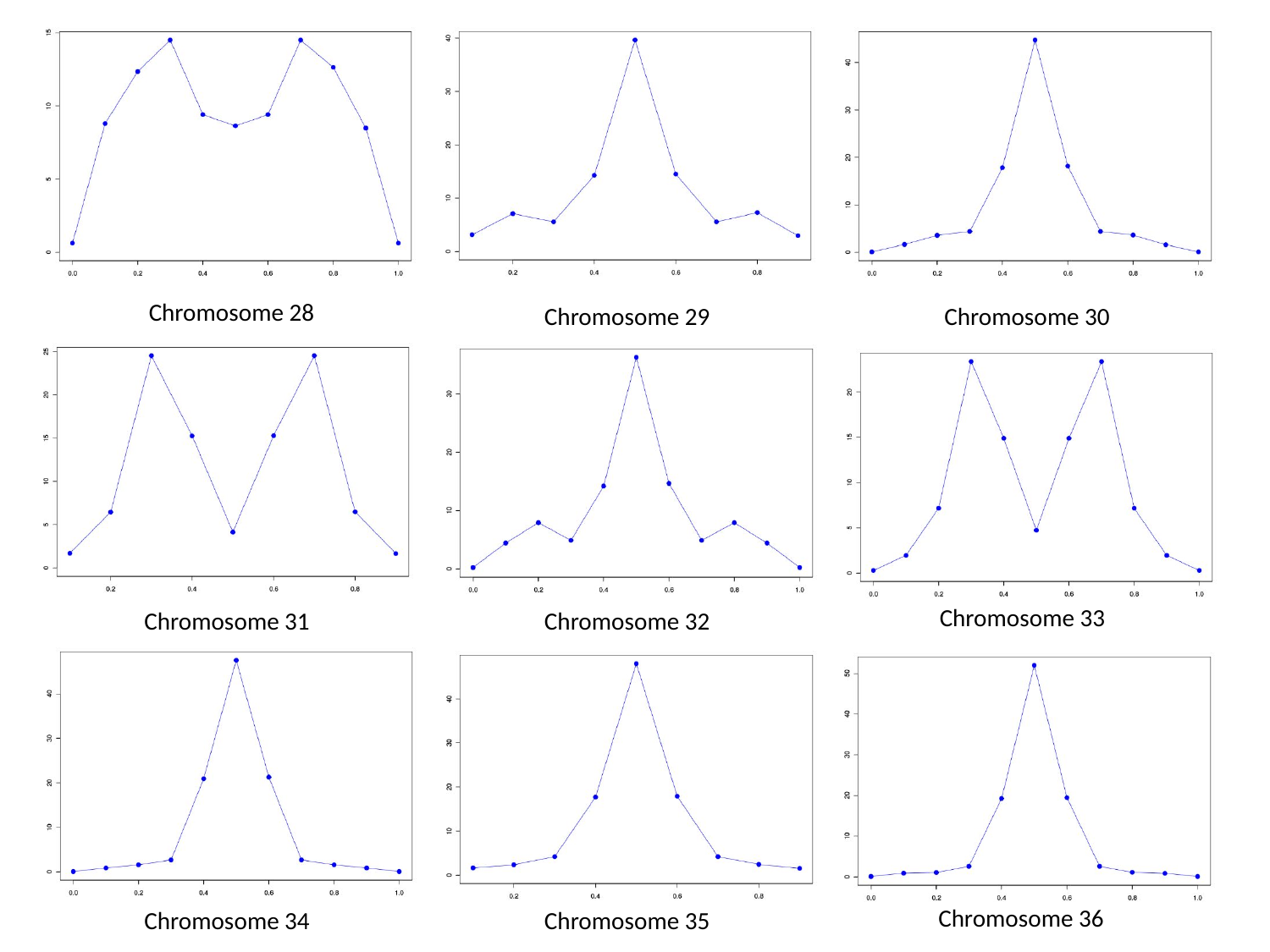

Chromosome 28
Chromosome 30
Chromosome 29
Chromosome 33
Chromosome 32
Chromosome 31
Chromosome 36
Chromosome 35
Chromosome 34

## Slide 37
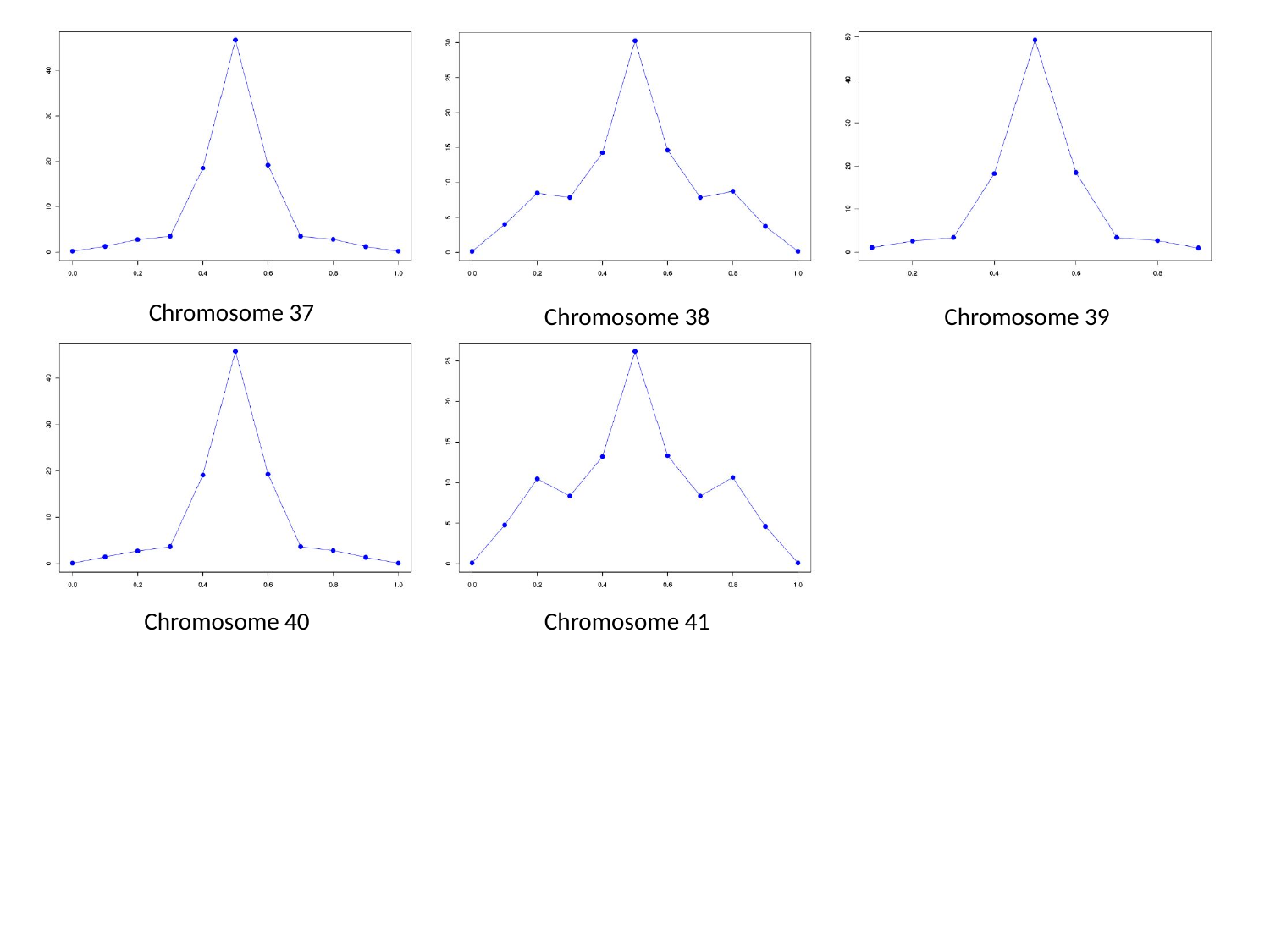

Chromosome 37
Chromosome 39
Chromosome 38
Chromosome 41
Chromosome 40
