# Supplementary material for: Chromosomal copy number variation reveals differential levels of genomic plasticity in distinct Trypanosoma cruzi strains
Source: BMC Genomics. 2015 Jul 4;16(1):499. doi: 10.1186/s12864-015-1680-4 (PMC4491234; doi:10.1186/s12864-015-1680-4)

## Slide 1
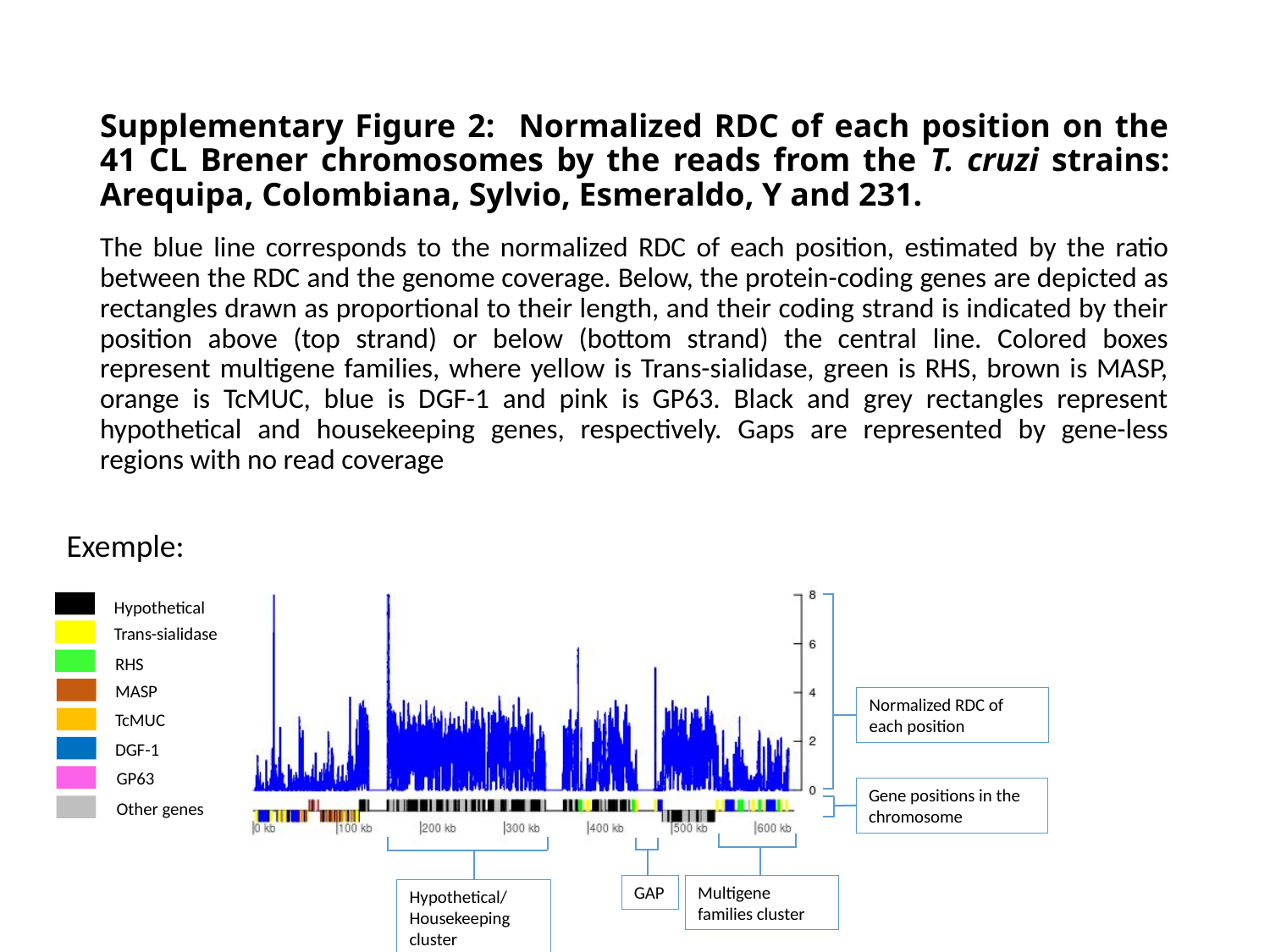

# Supplementary Figure 2: Normalized RDC of each position on the 41 CL Brener chromosomes by the reads from the T. cruzi strains: Arequipa, Colombiana, Sylvio, Esmeraldo, Y and 231.
The blue line corresponds to the normalized RDC of each position, estimated by the ratio between the RDC and the genome coverage. Below, the protein-coding genes are depicted as rectangles drawn as proportional to their length, and their coding strand is indicated by their position above (top strand) or below (bottom strand) the central line. Colored boxes represent multigene families, where yellow is Trans-sialidase, green is RHS, brown is MASP, orange is TcMUC, blue is DGF-1 and pink is GP63. Black and grey rectangles represent hypothetical and housekeeping genes, respectively. Gaps are represented by gene-less regions with no read coverage
Exemple:
Hypothetical
Trans-sialidase
RHS
MASP
TcMUC
DGF-1
GP63
Other genes
Normalized RDC of each position
Gene positions in the chromosome
GAP
Multigene families cluster
Hypothetical/Housekeeping cluster

## Slide 2
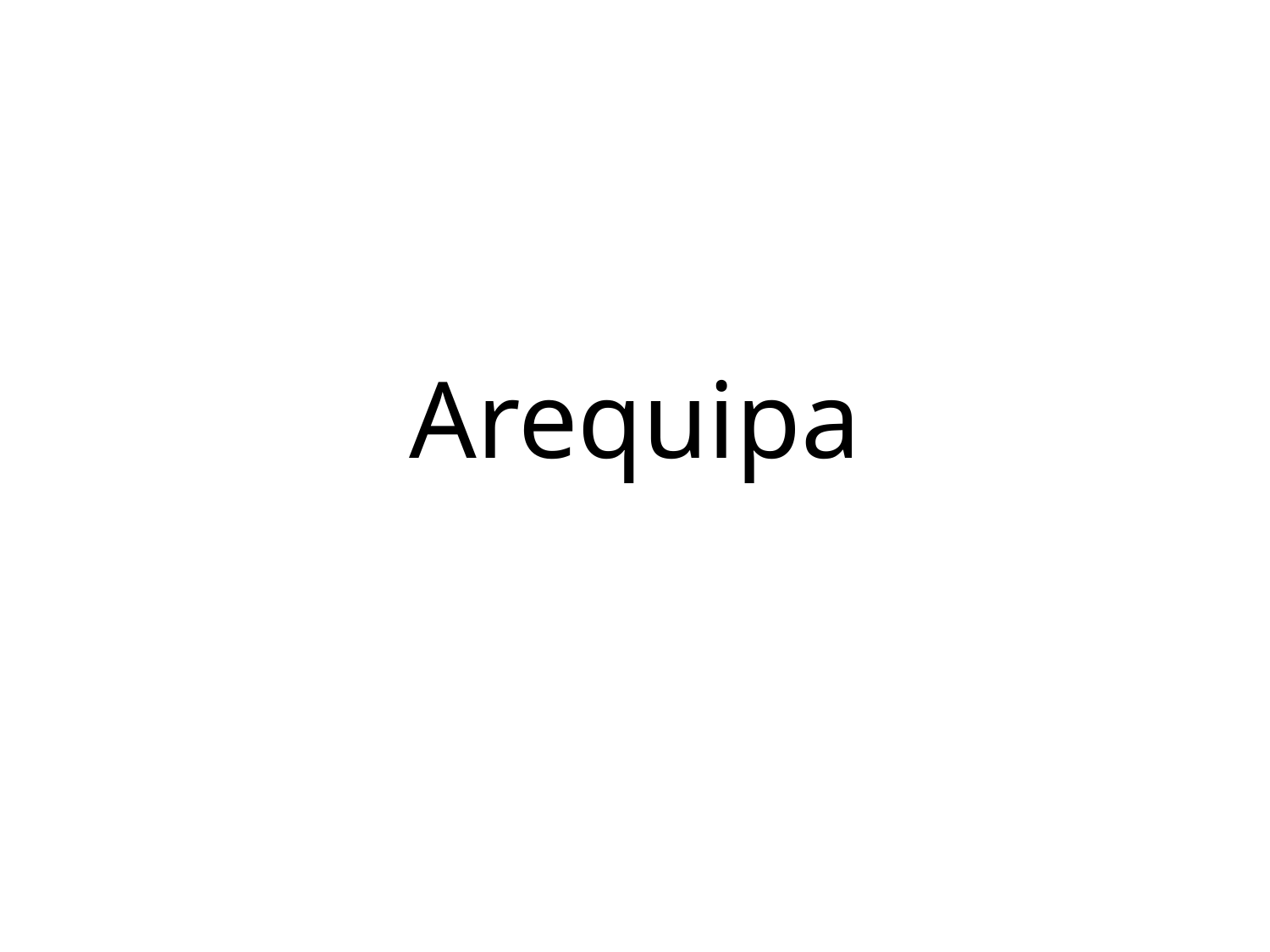

# Arequipa

## Slide 3
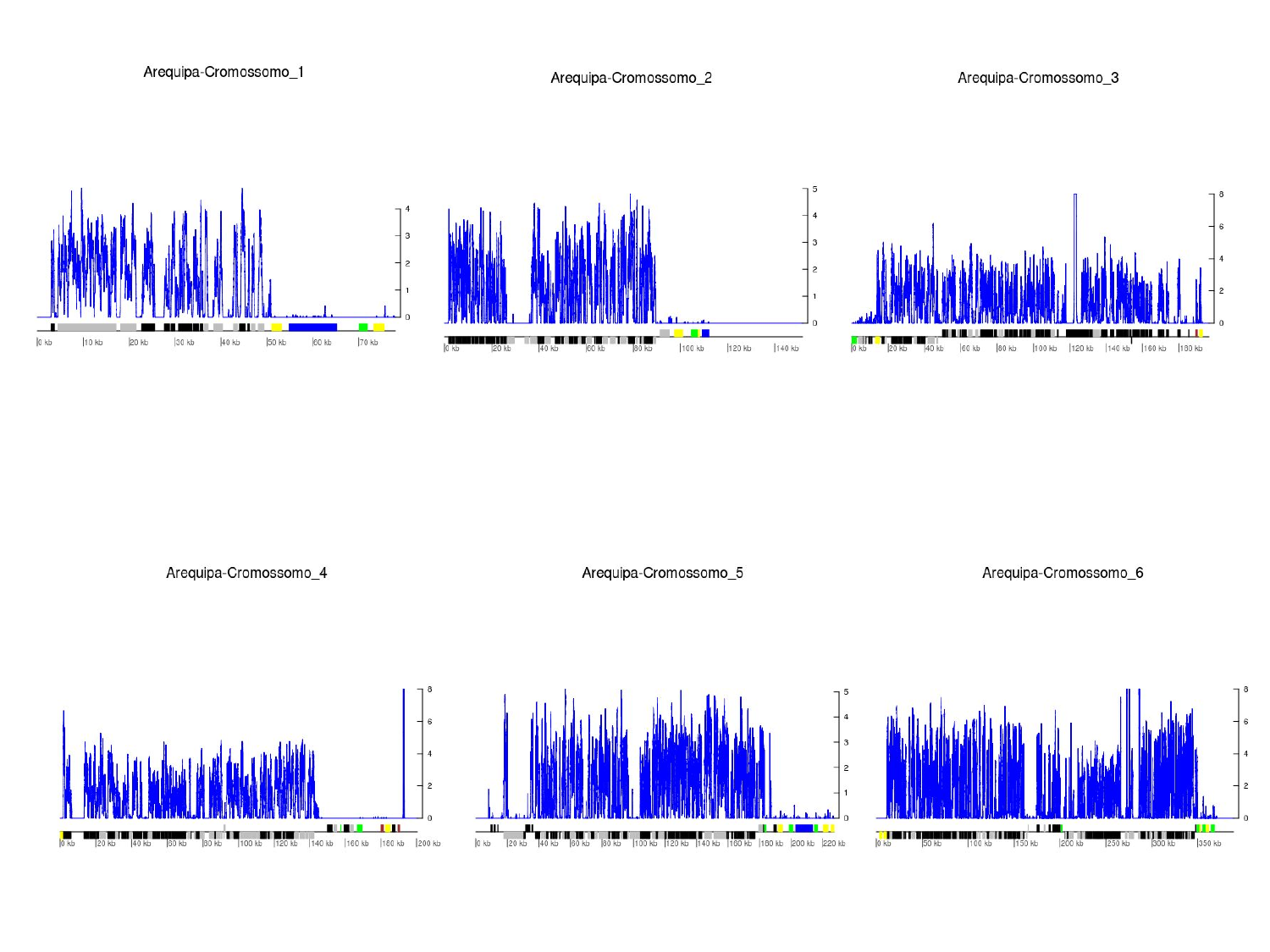

## Slide 4
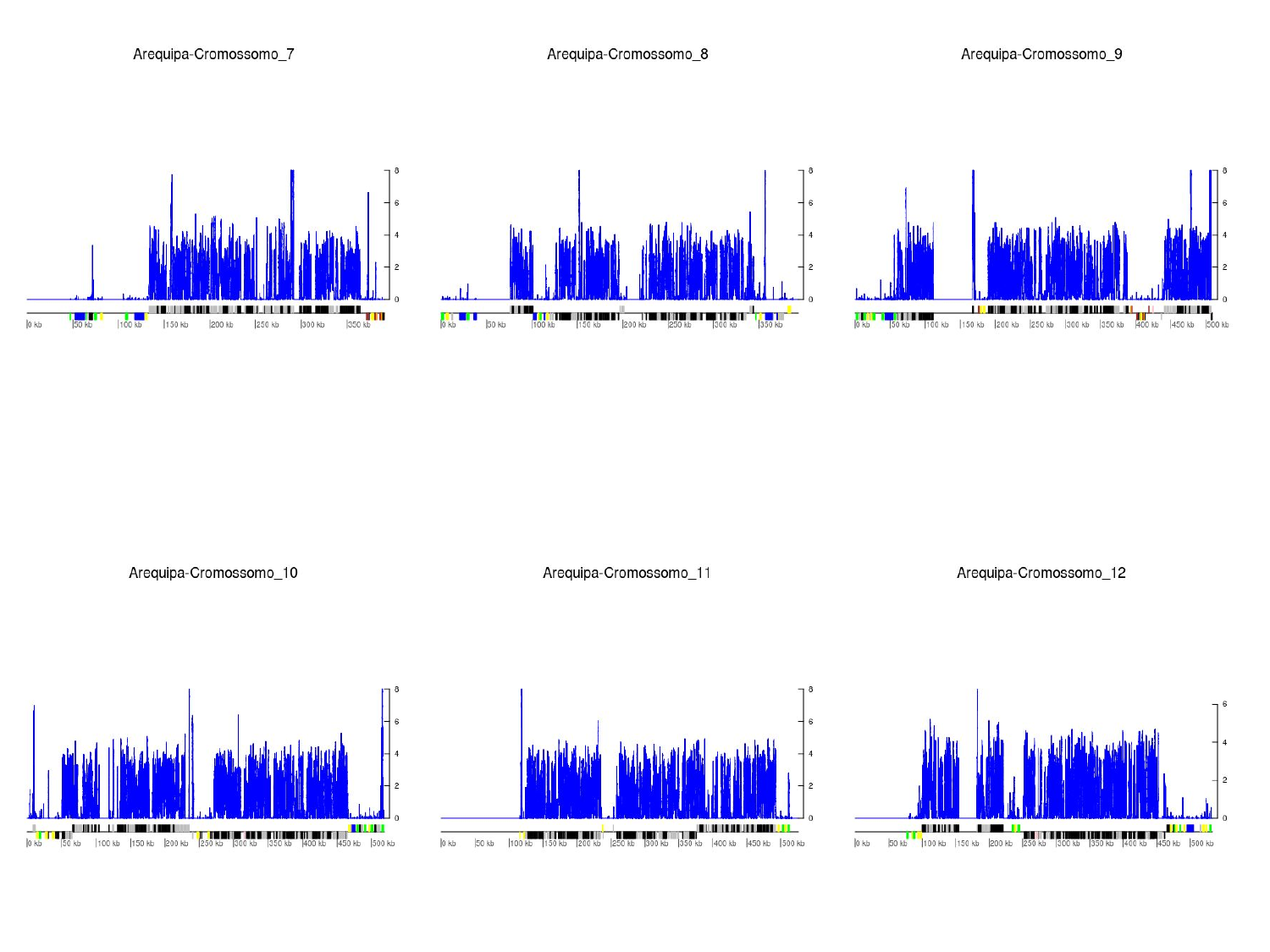

## Slide 5
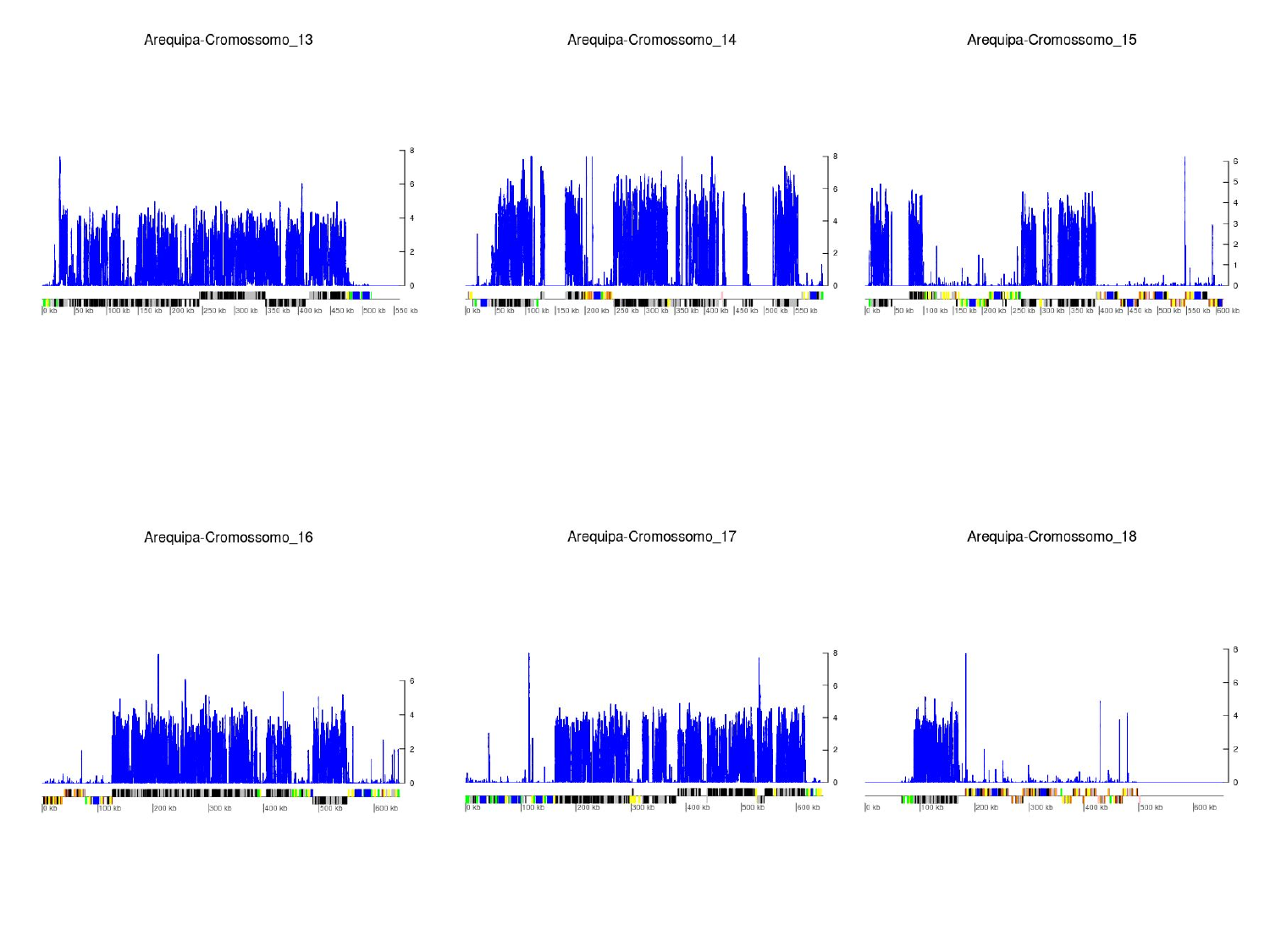

## Slide 6
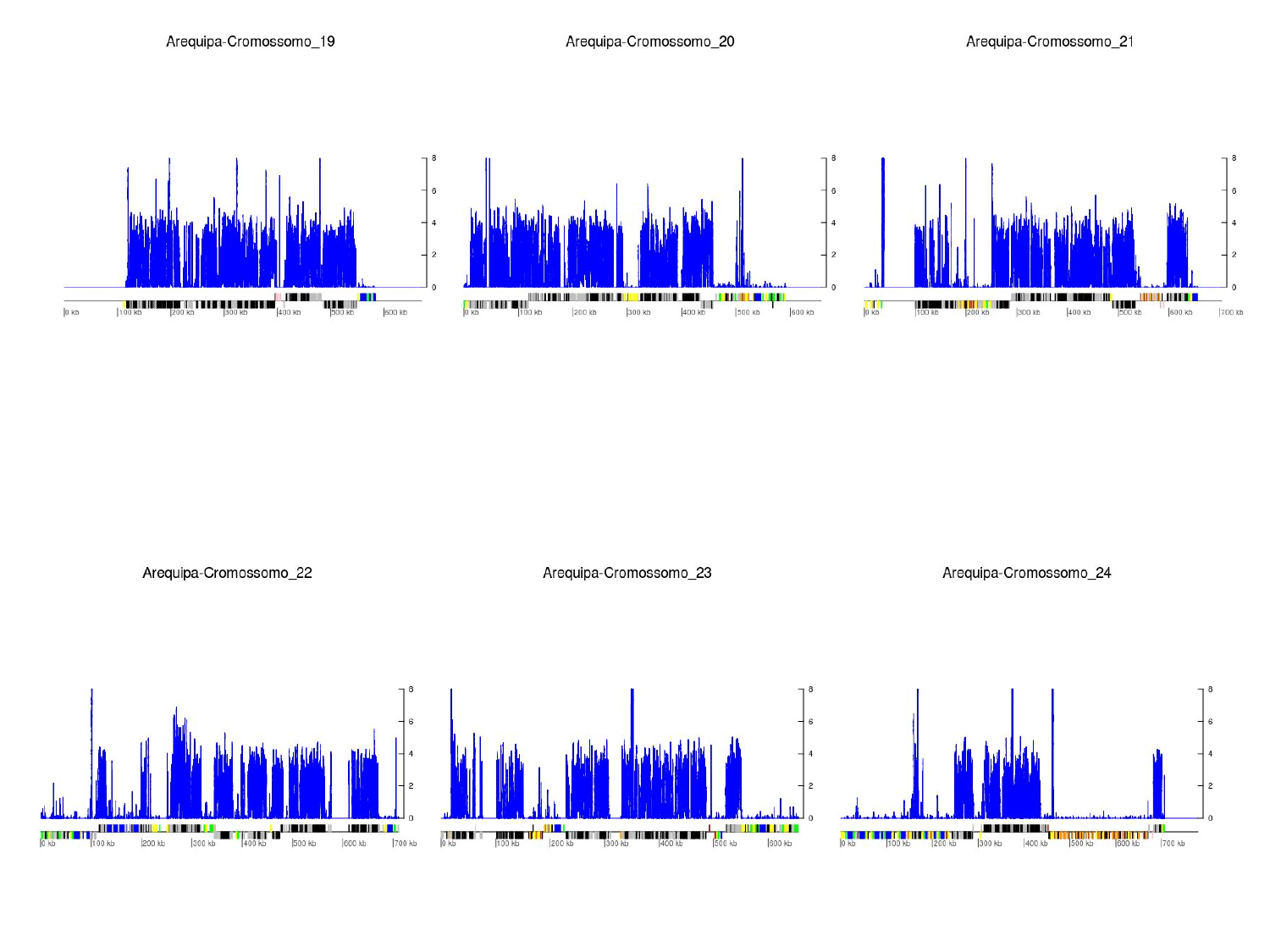

## Slide 7
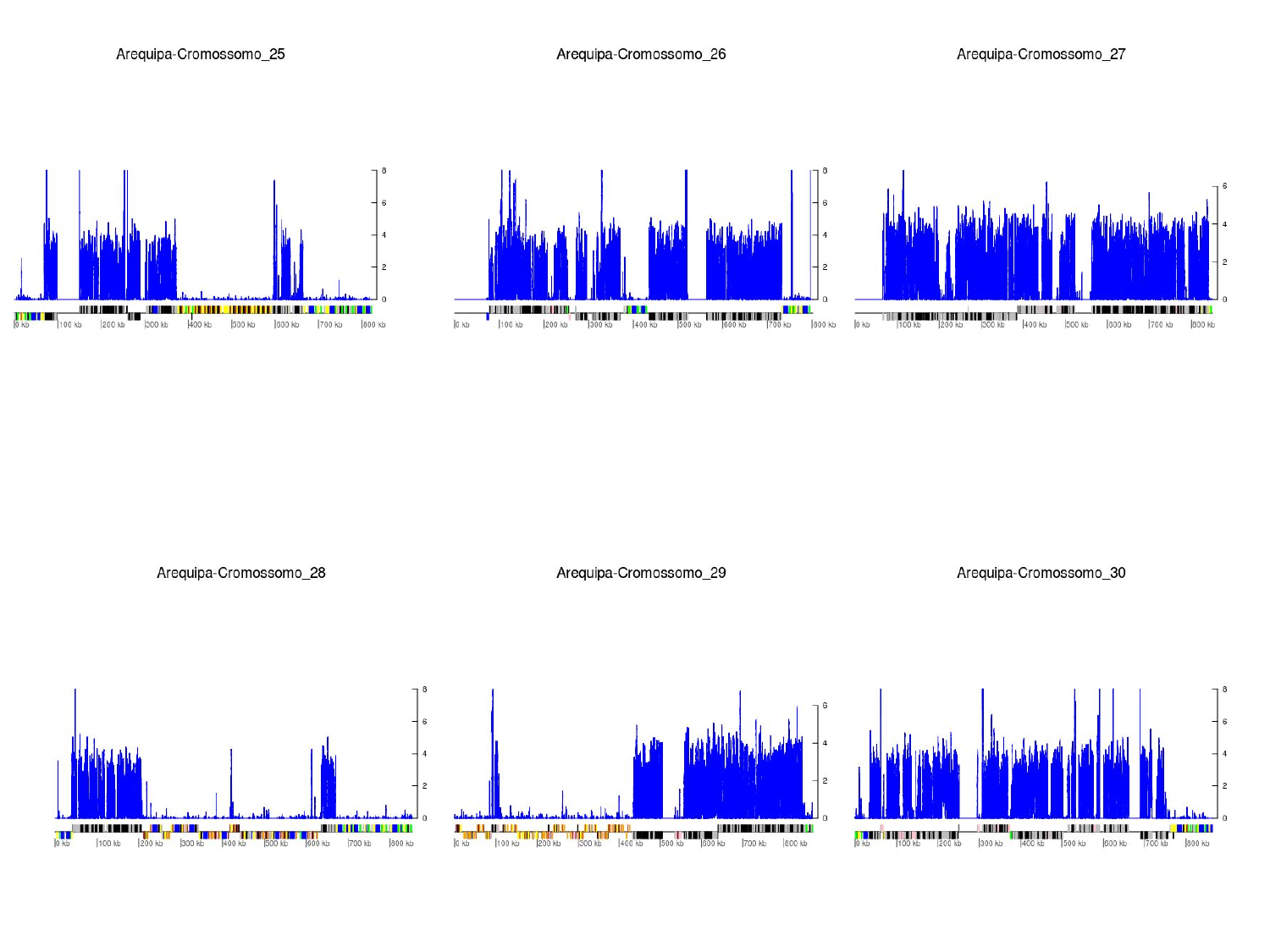

## Slide 8
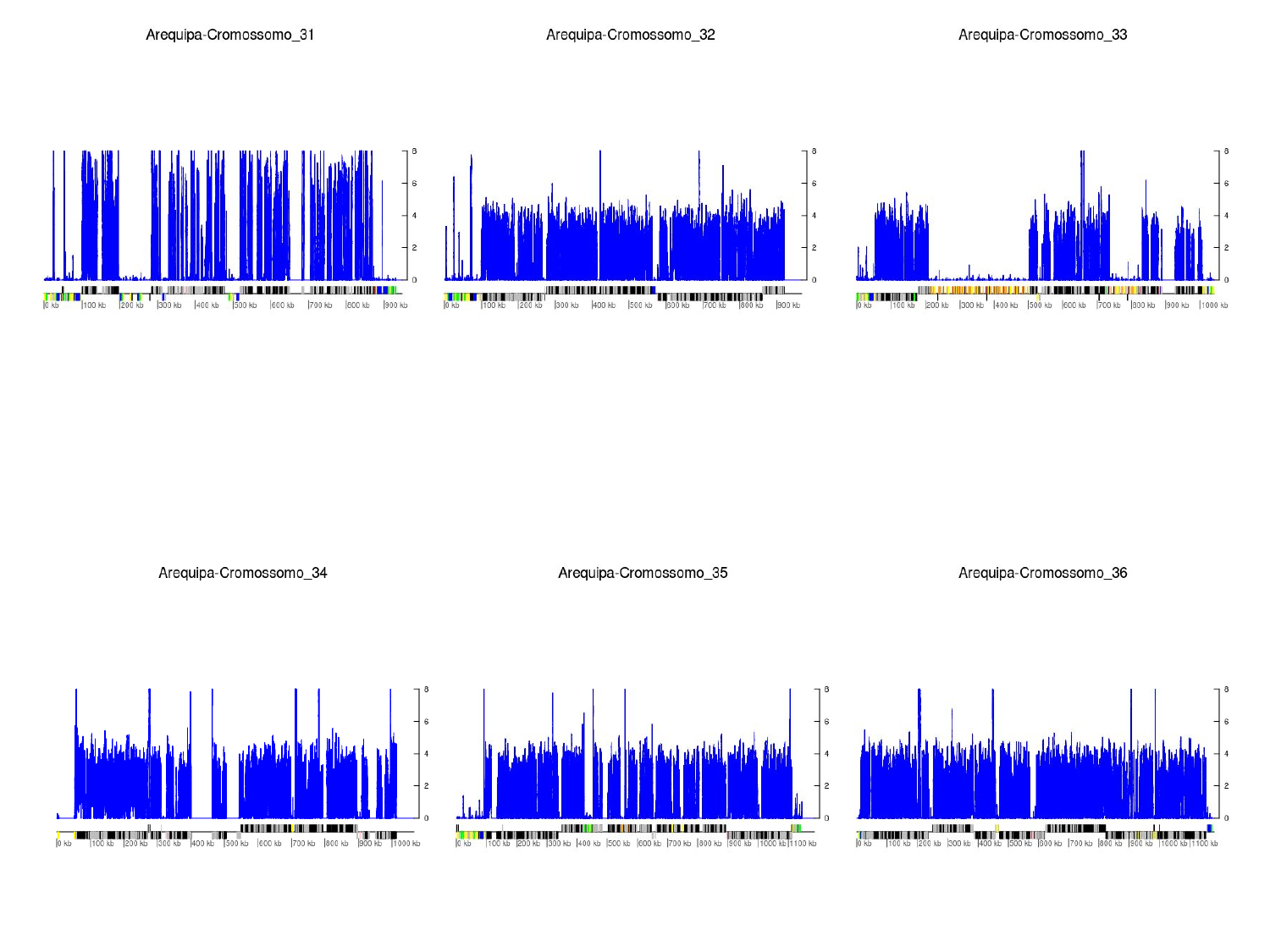

## Slide 9
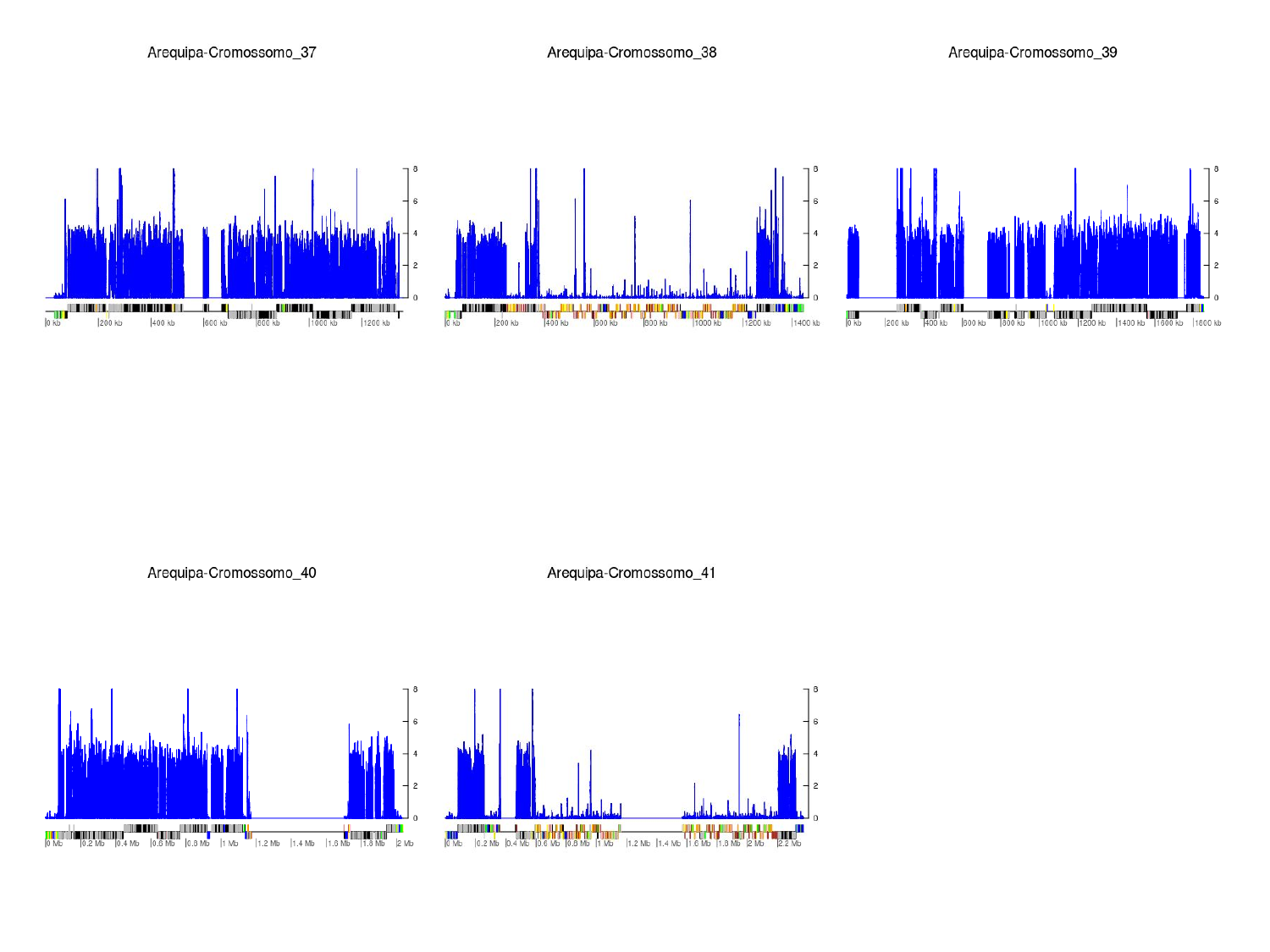

## Slide 10
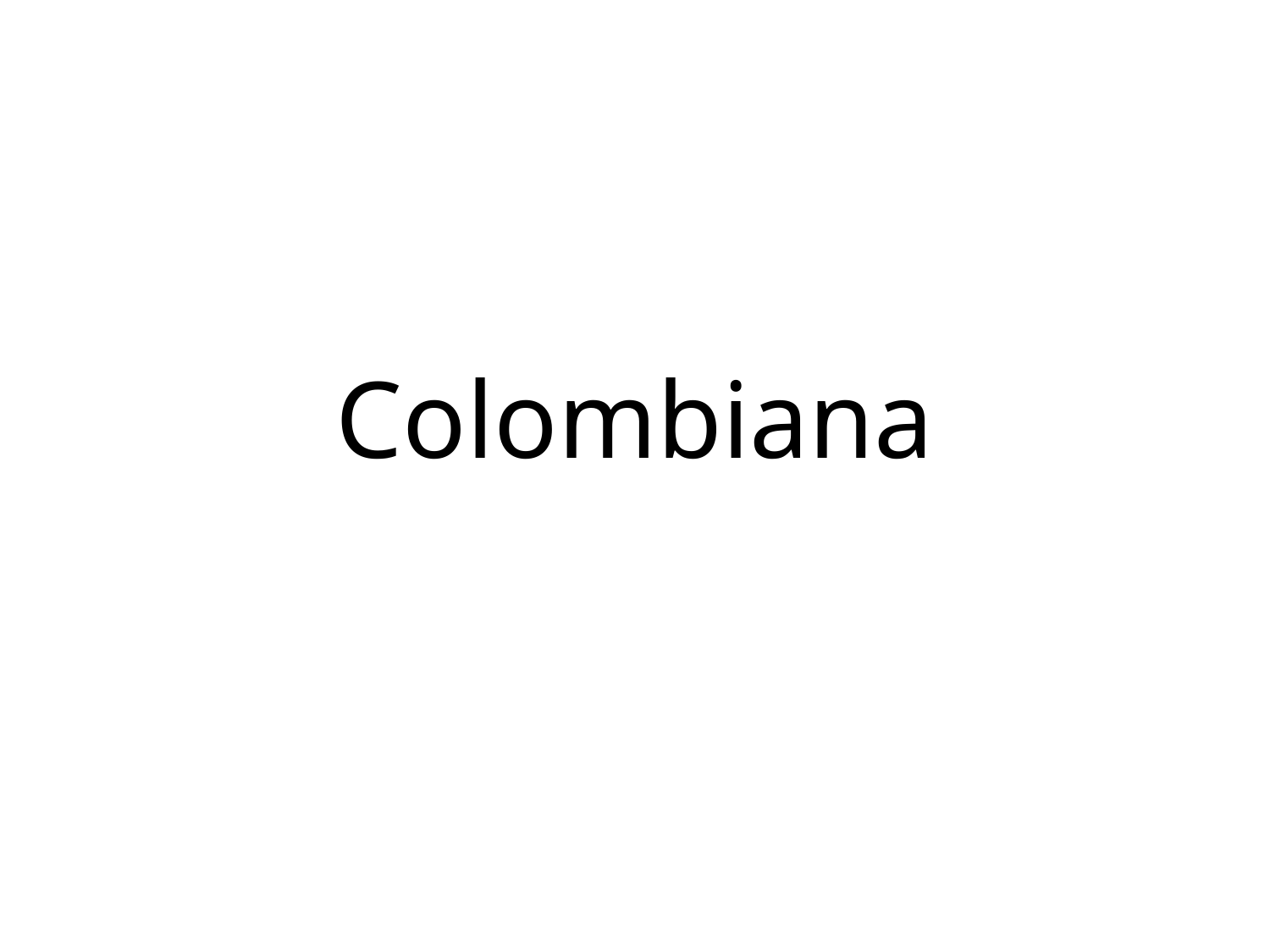

# Colombiana

## Slide 11
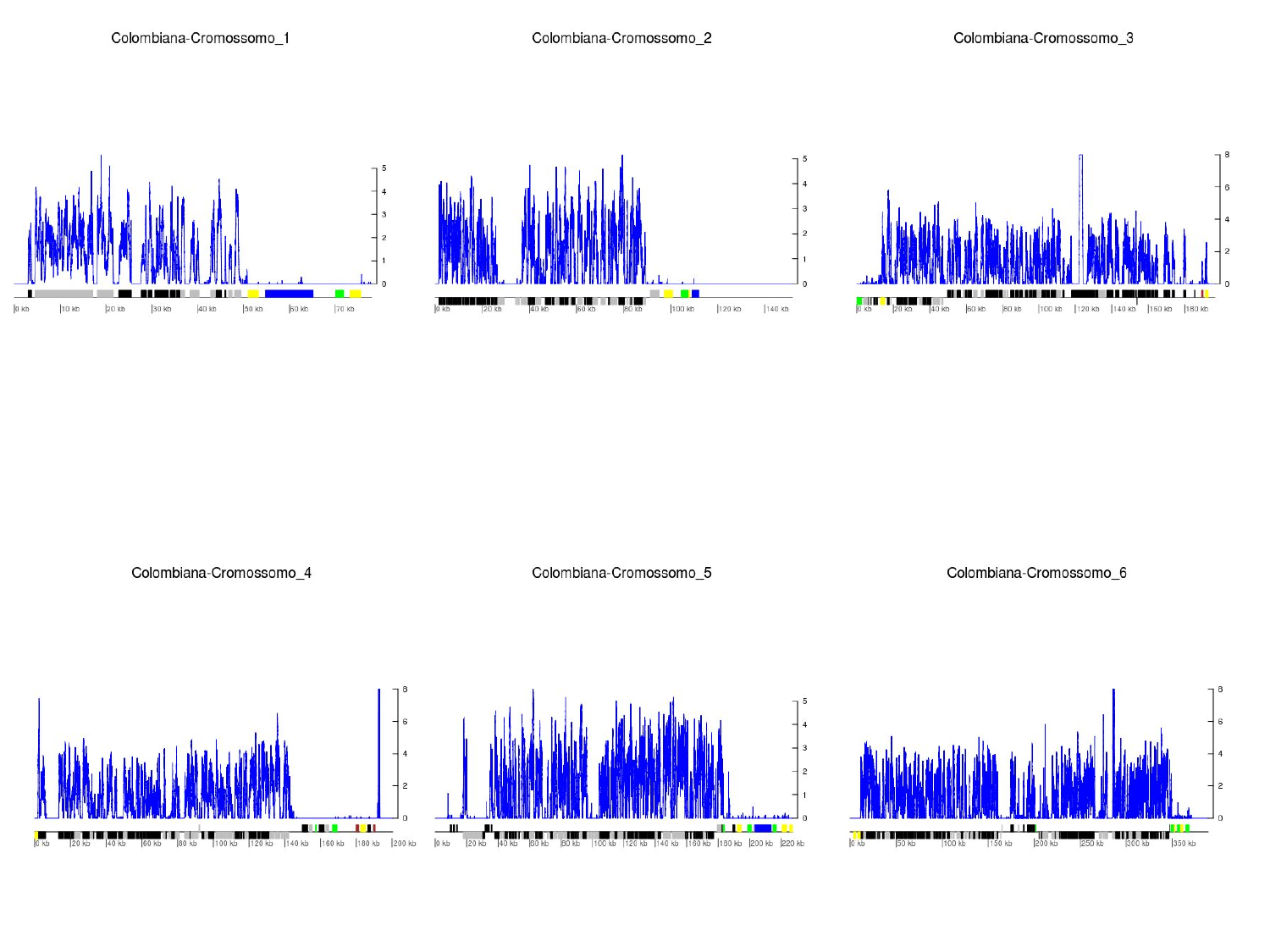

## Slide 12
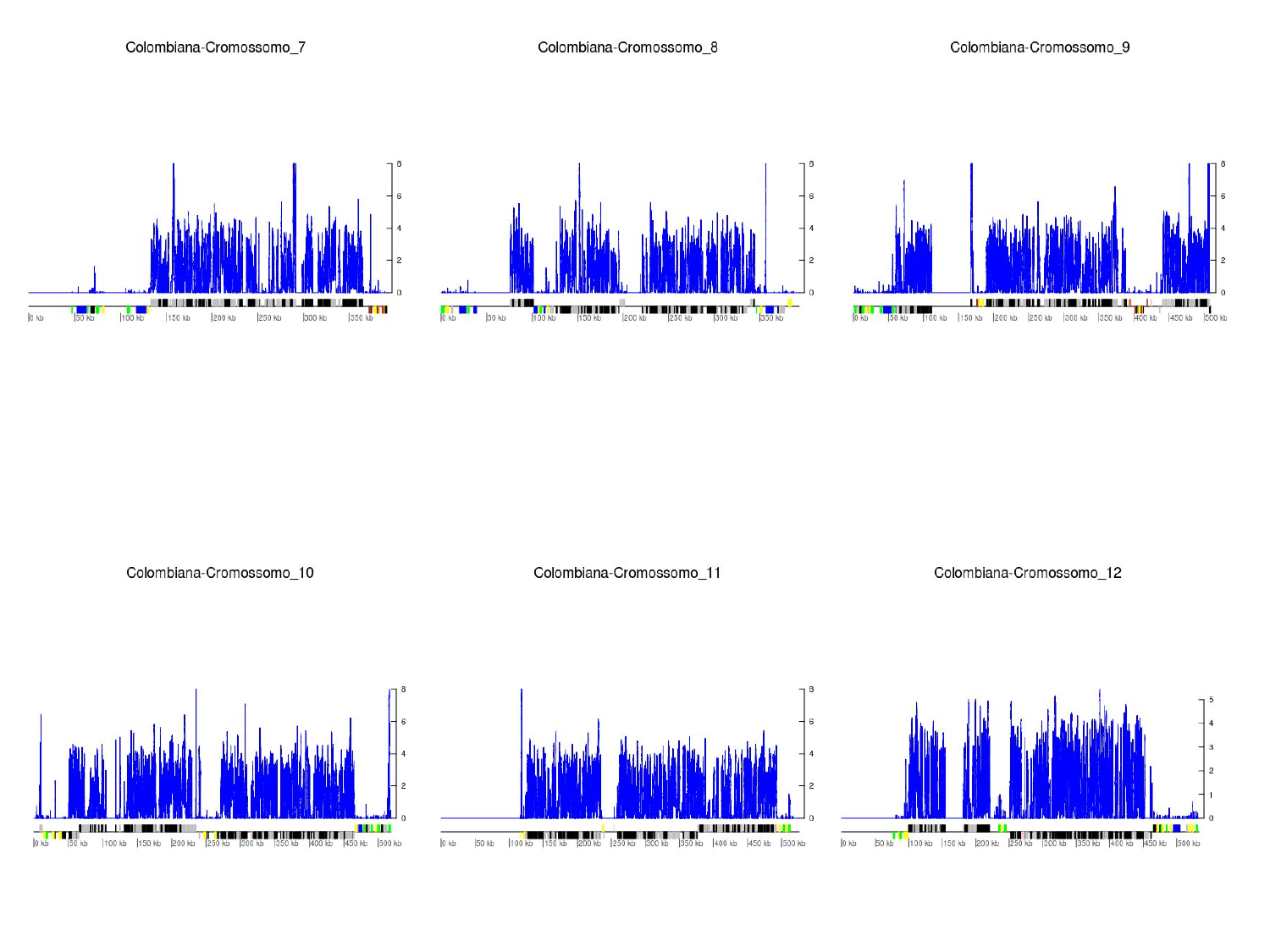

## Slide 13
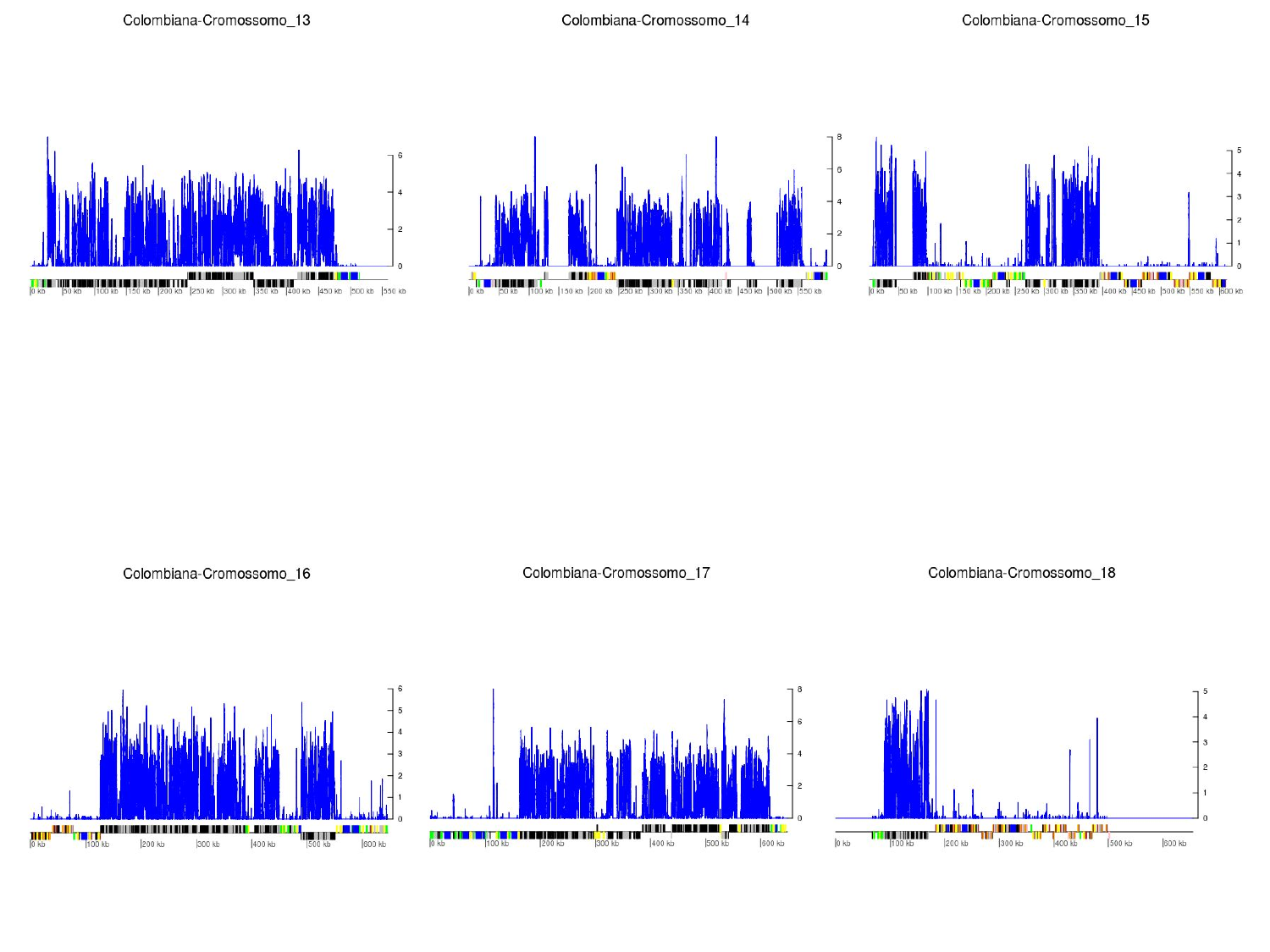

## Slide 14
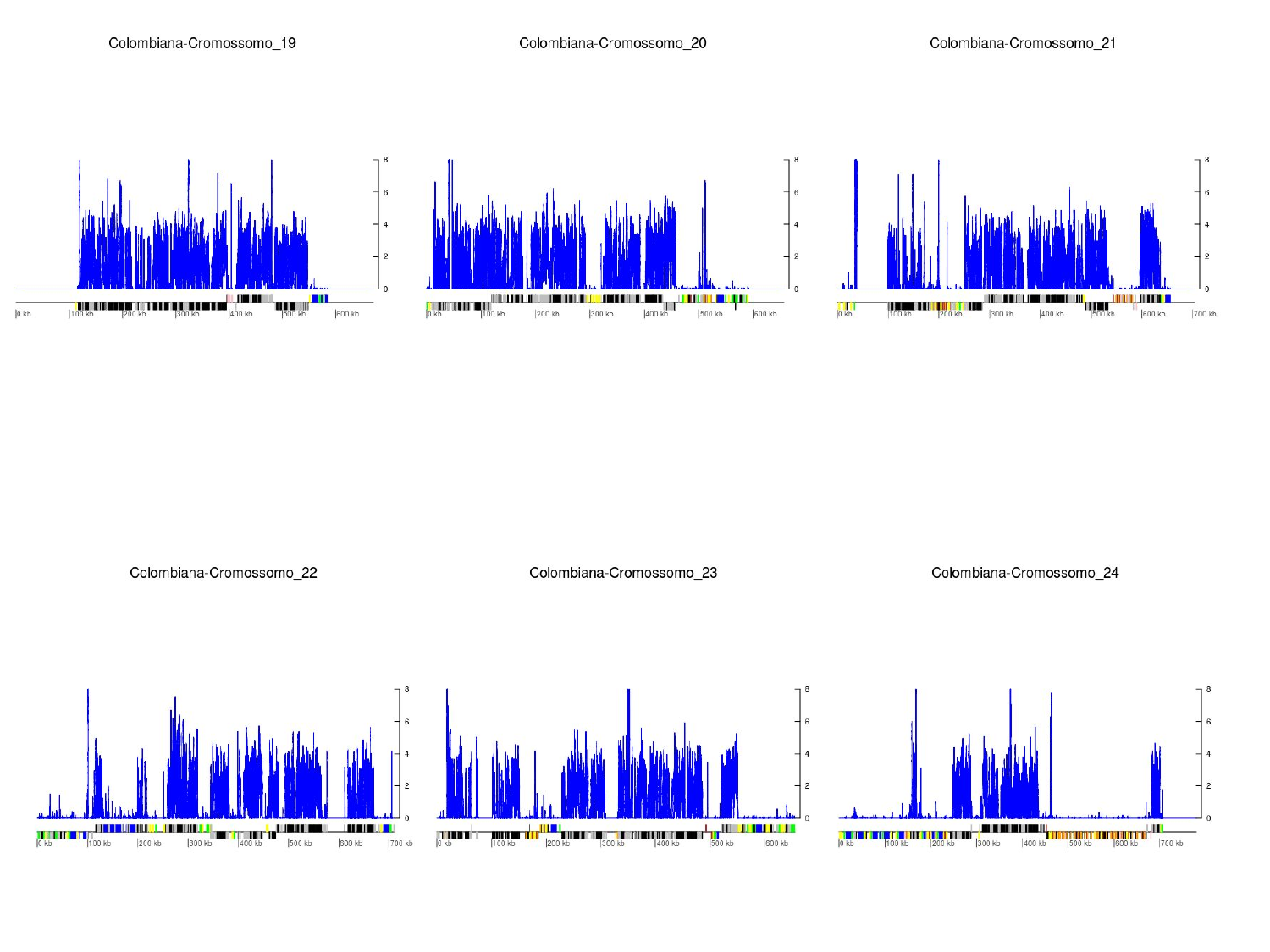

## Slide 15
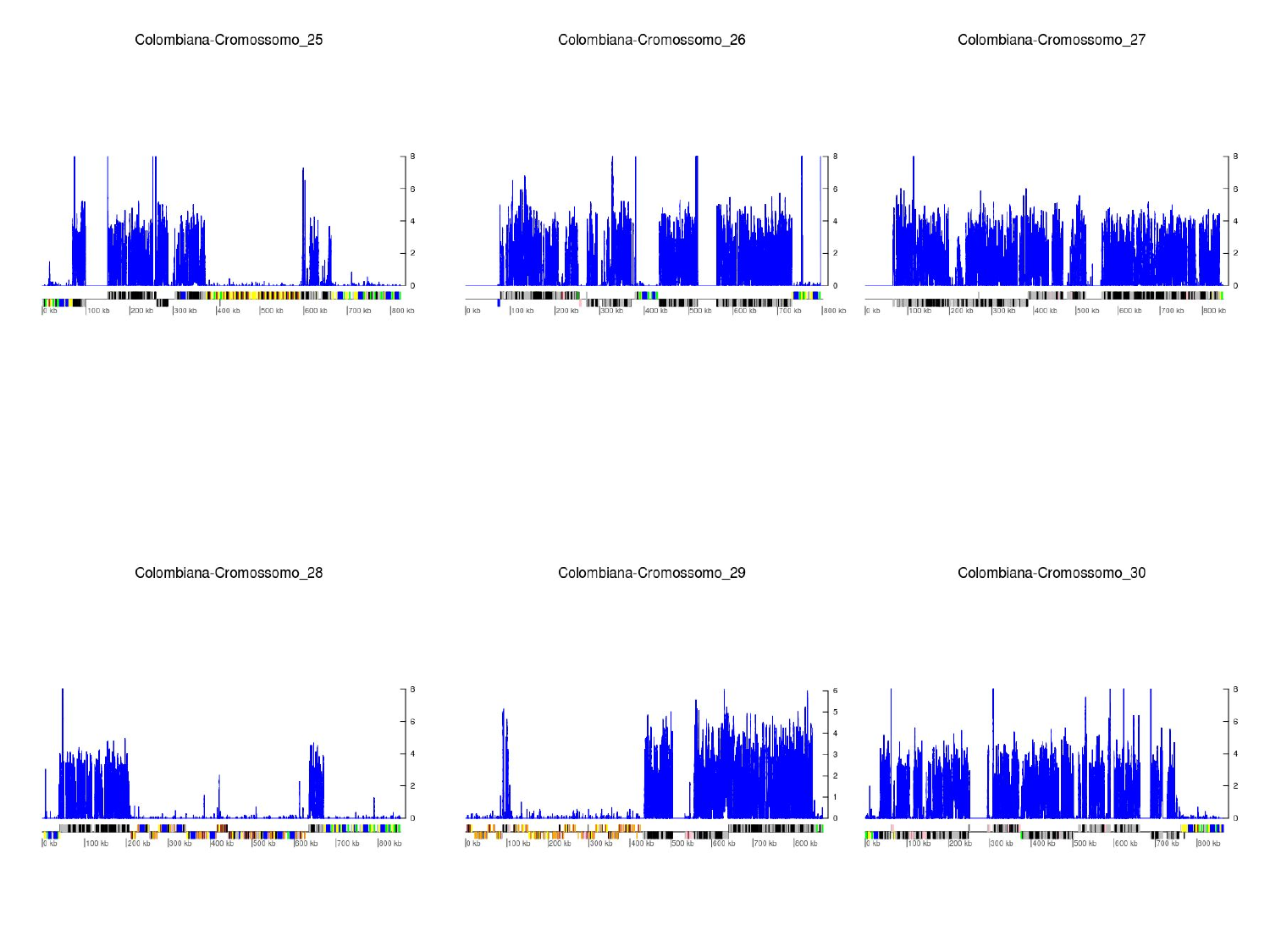

## Slide 16
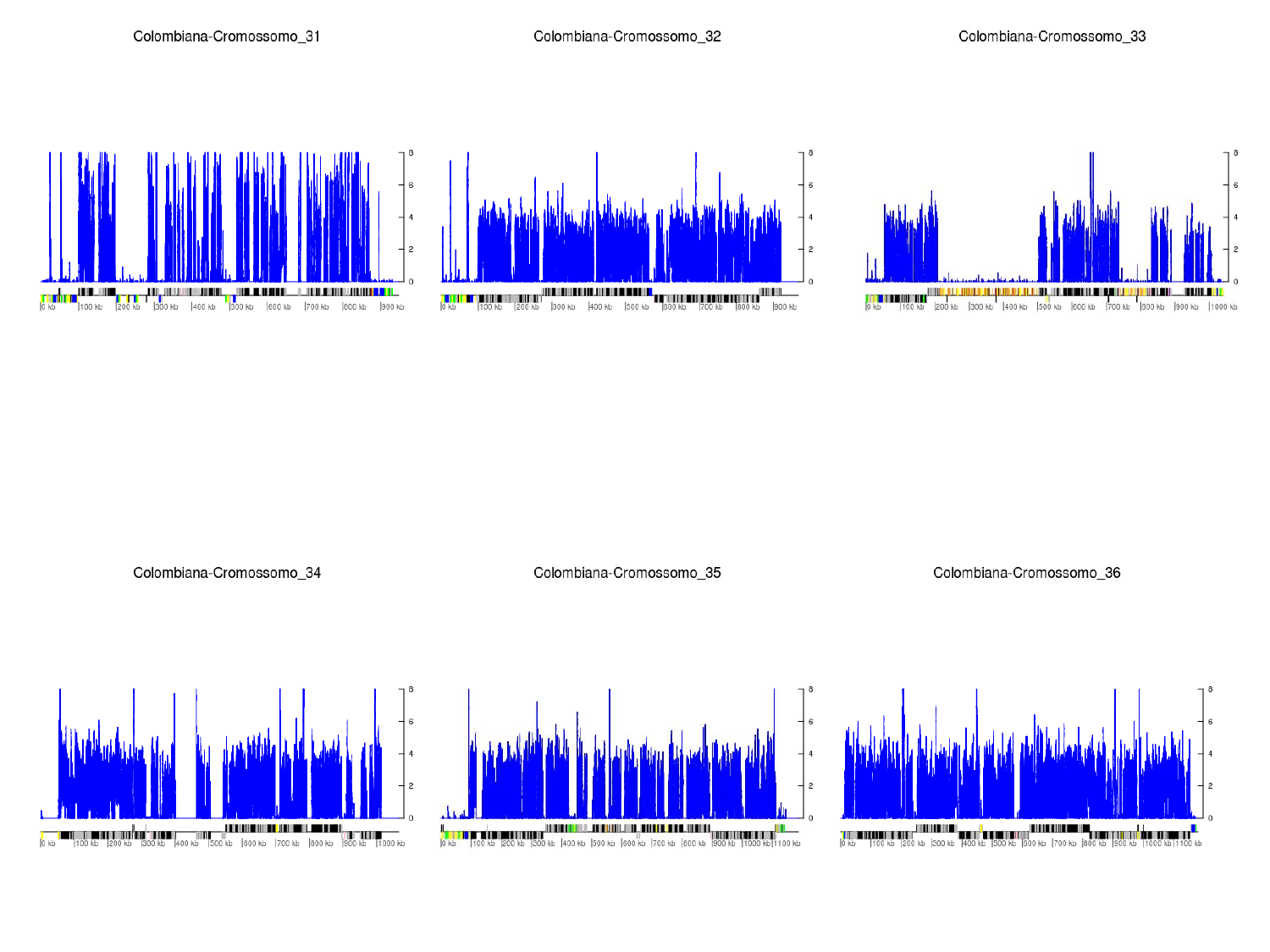

## Slide 17
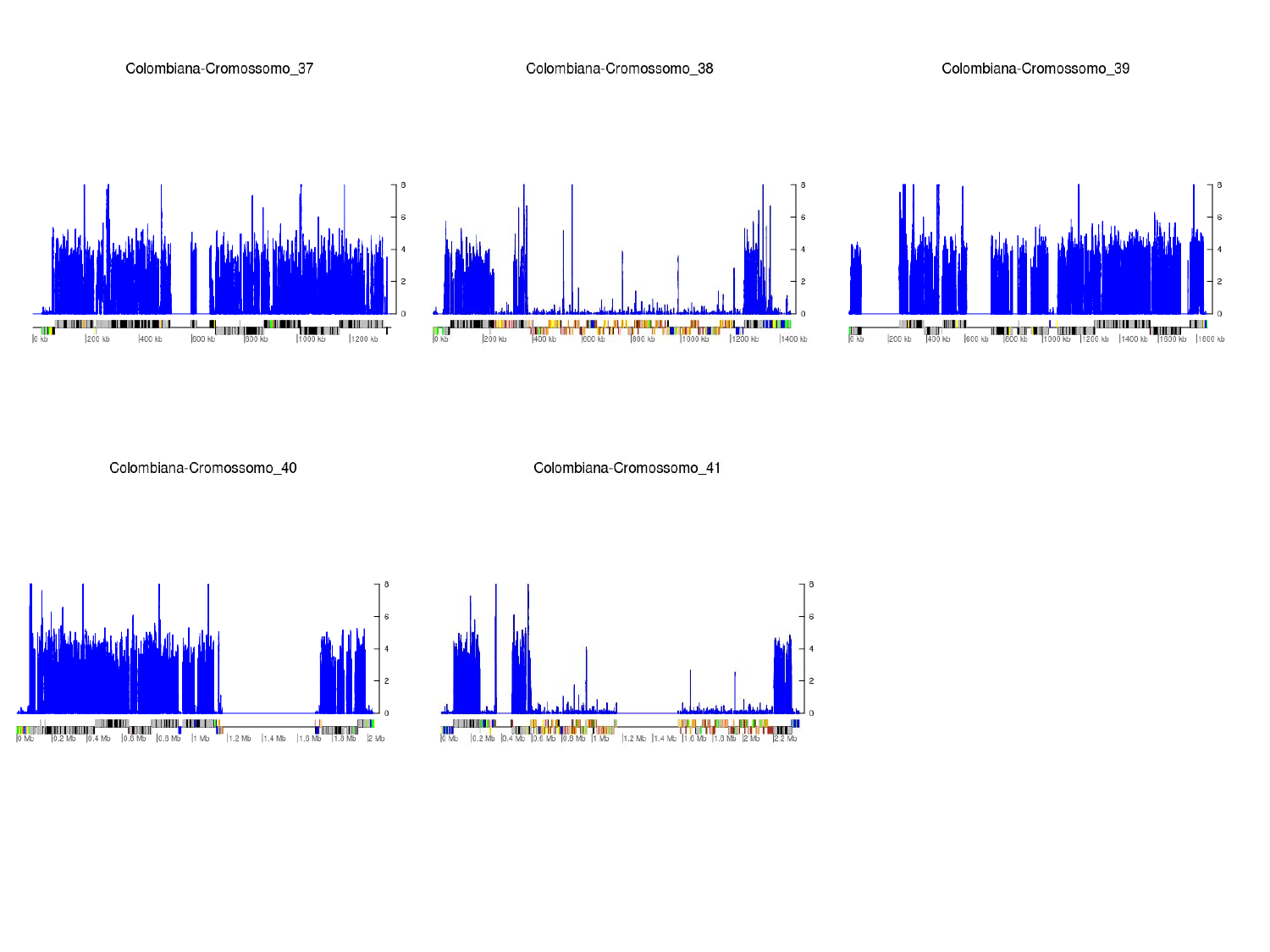

## Slide 18
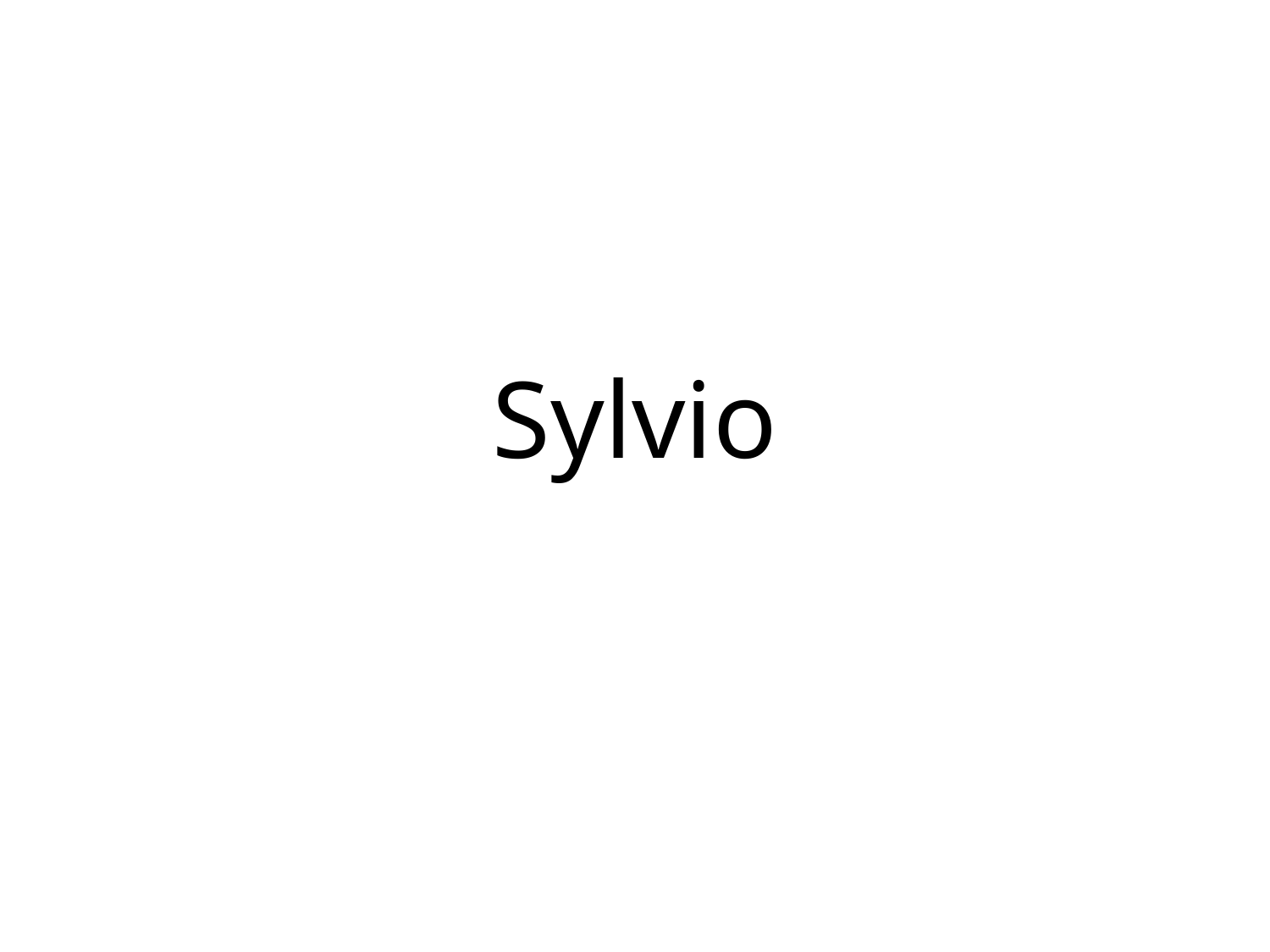

# Sylvio

## Slide 19
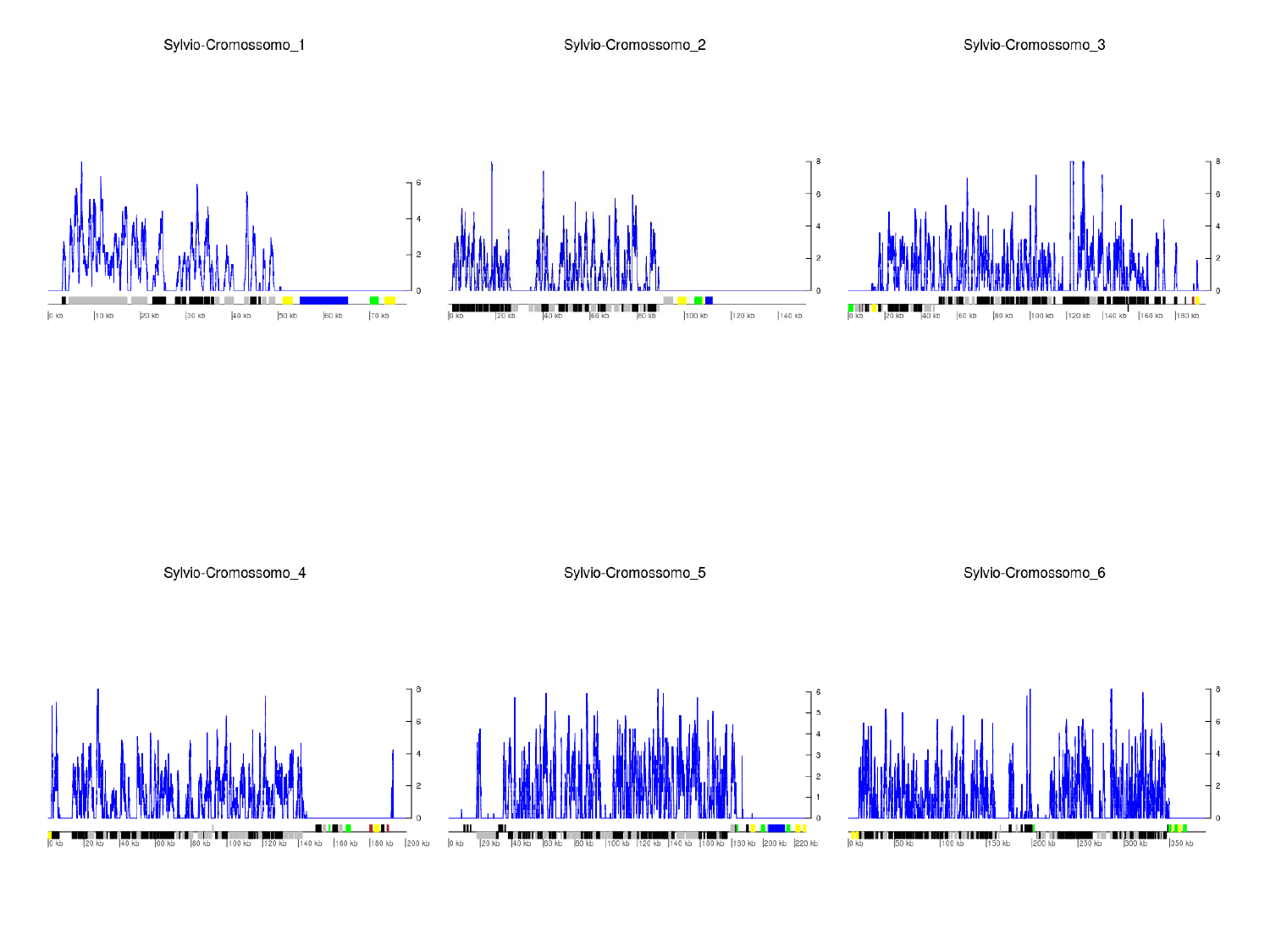

## Slide 20
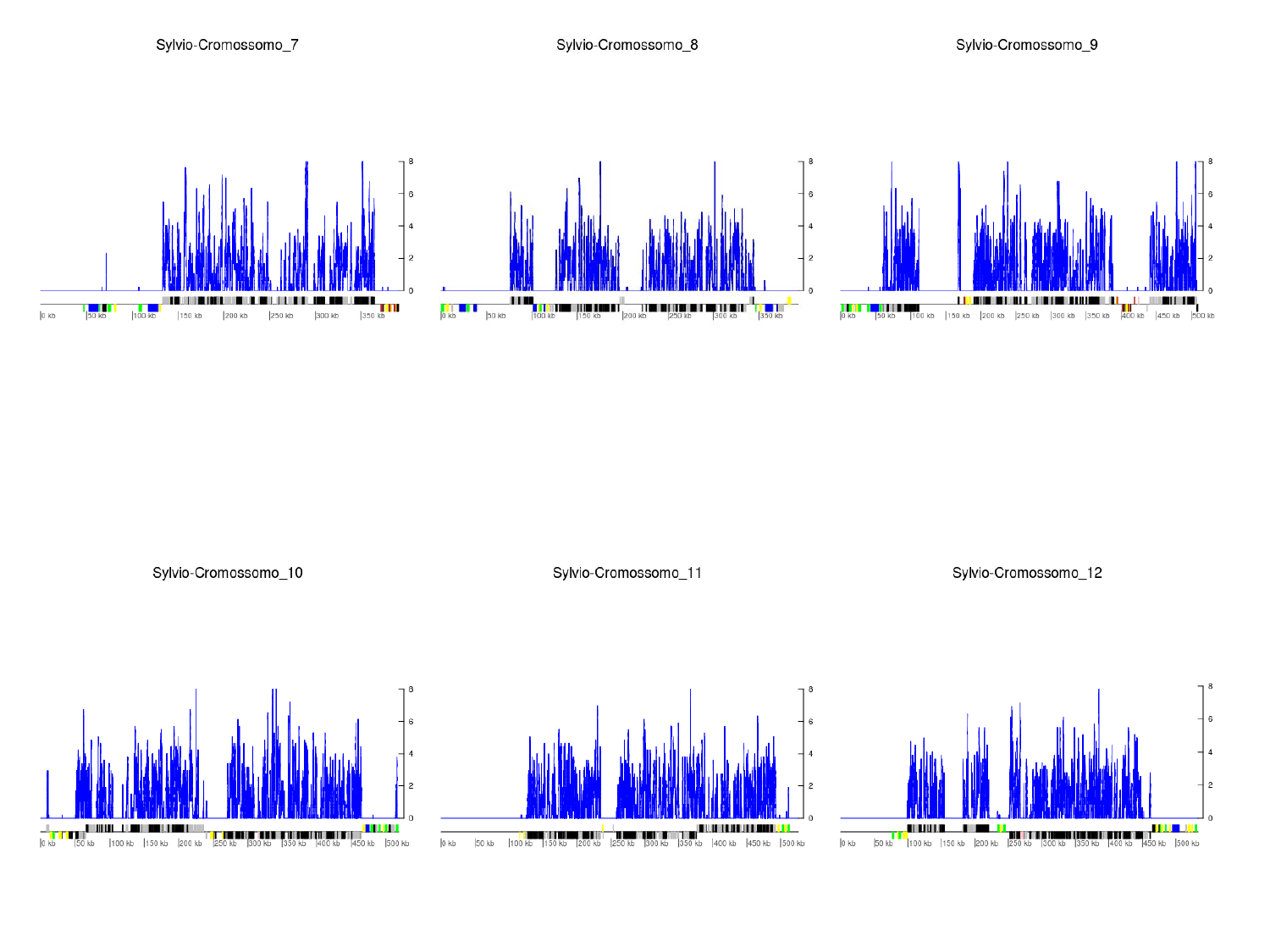

## Slide 21
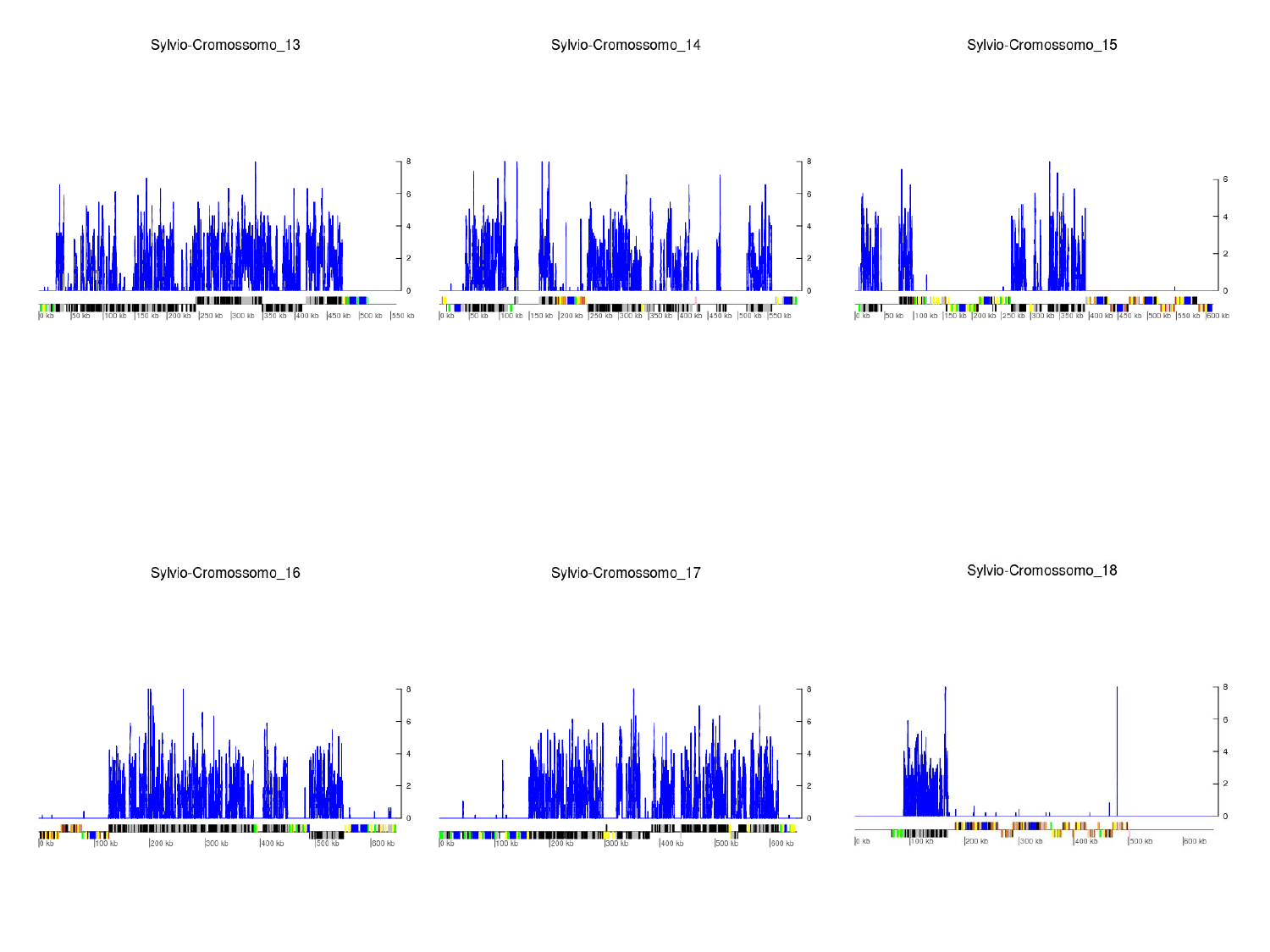

## Slide 22
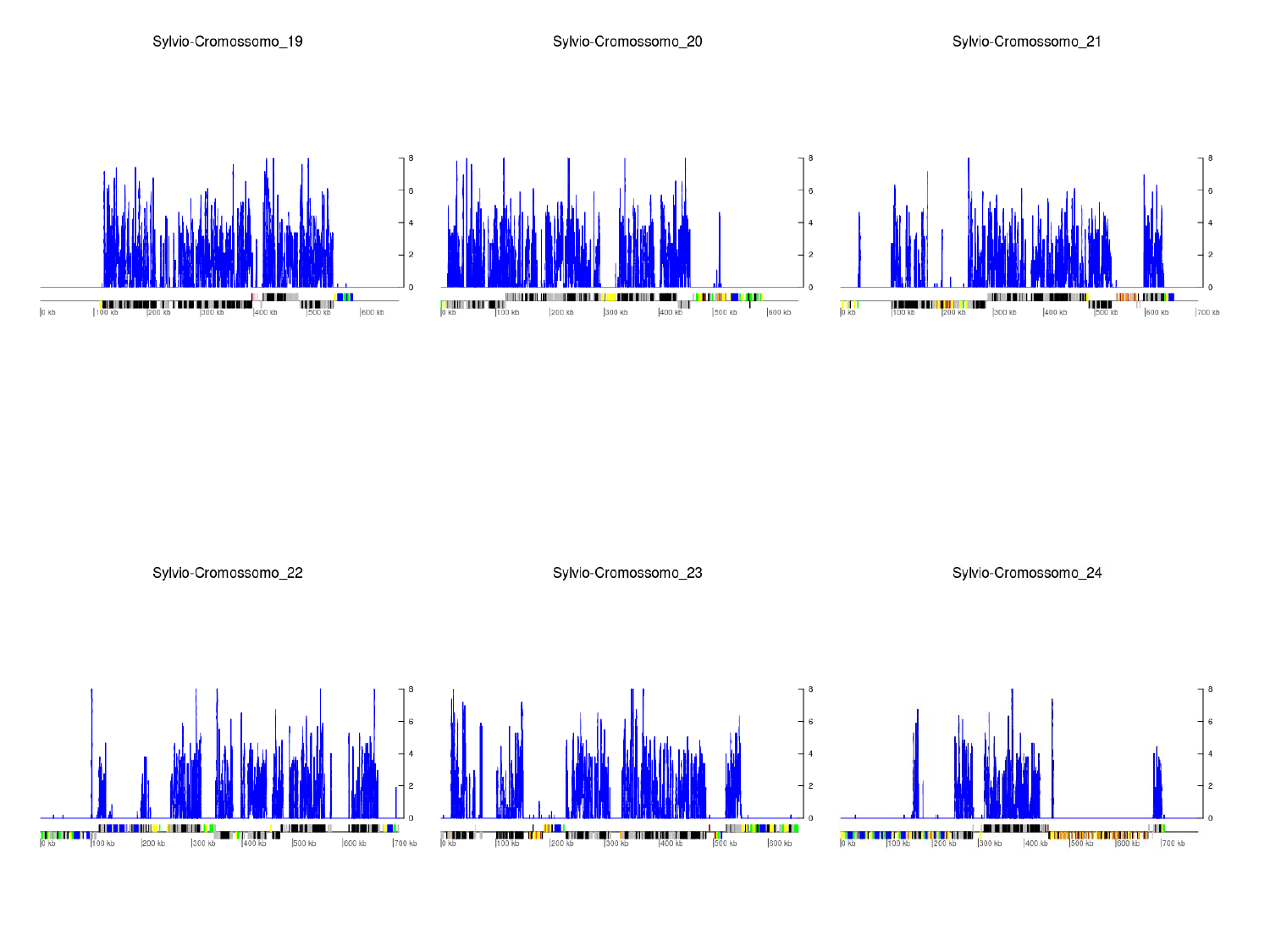

## Slide 23
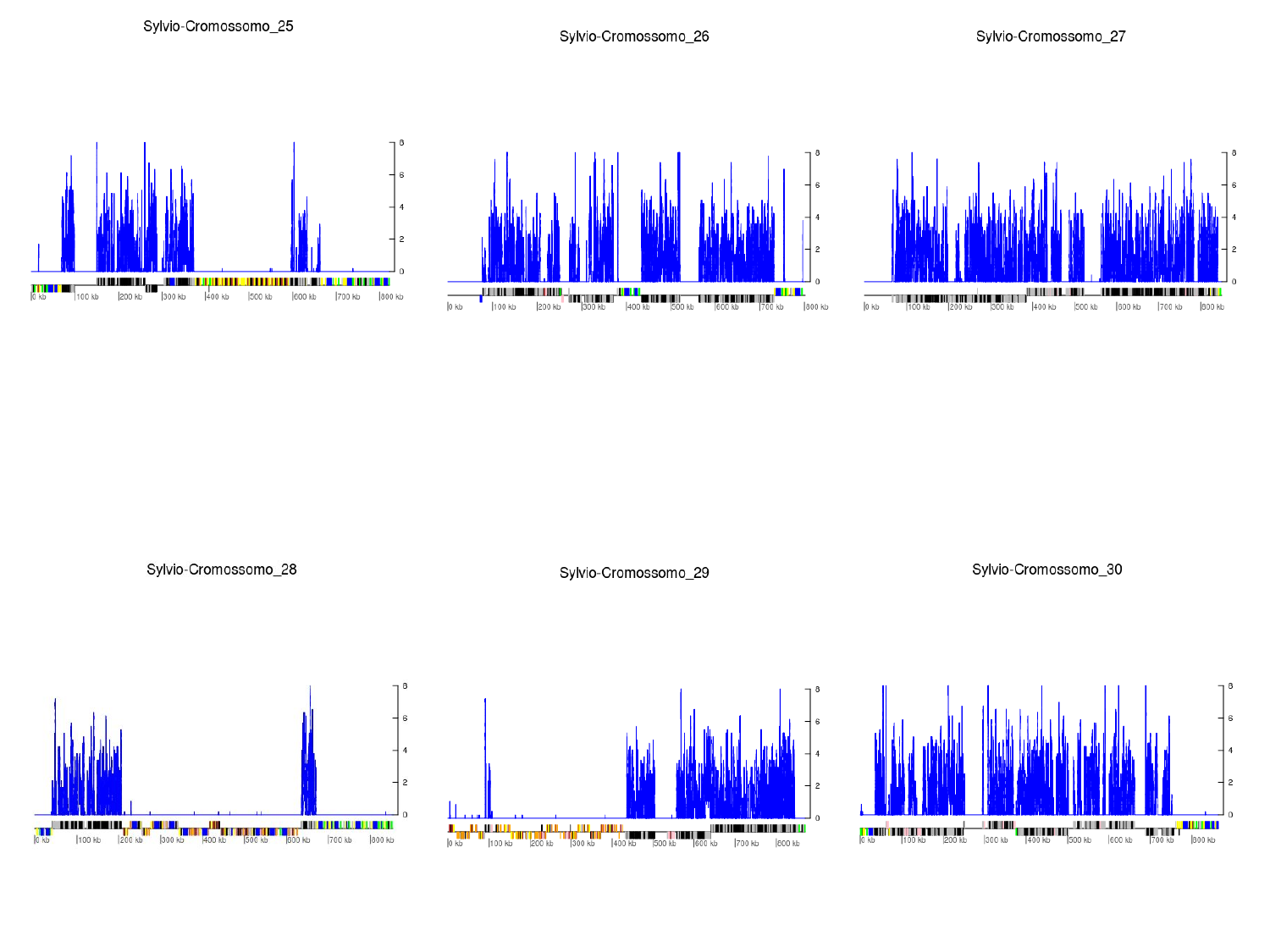

## Slide 24
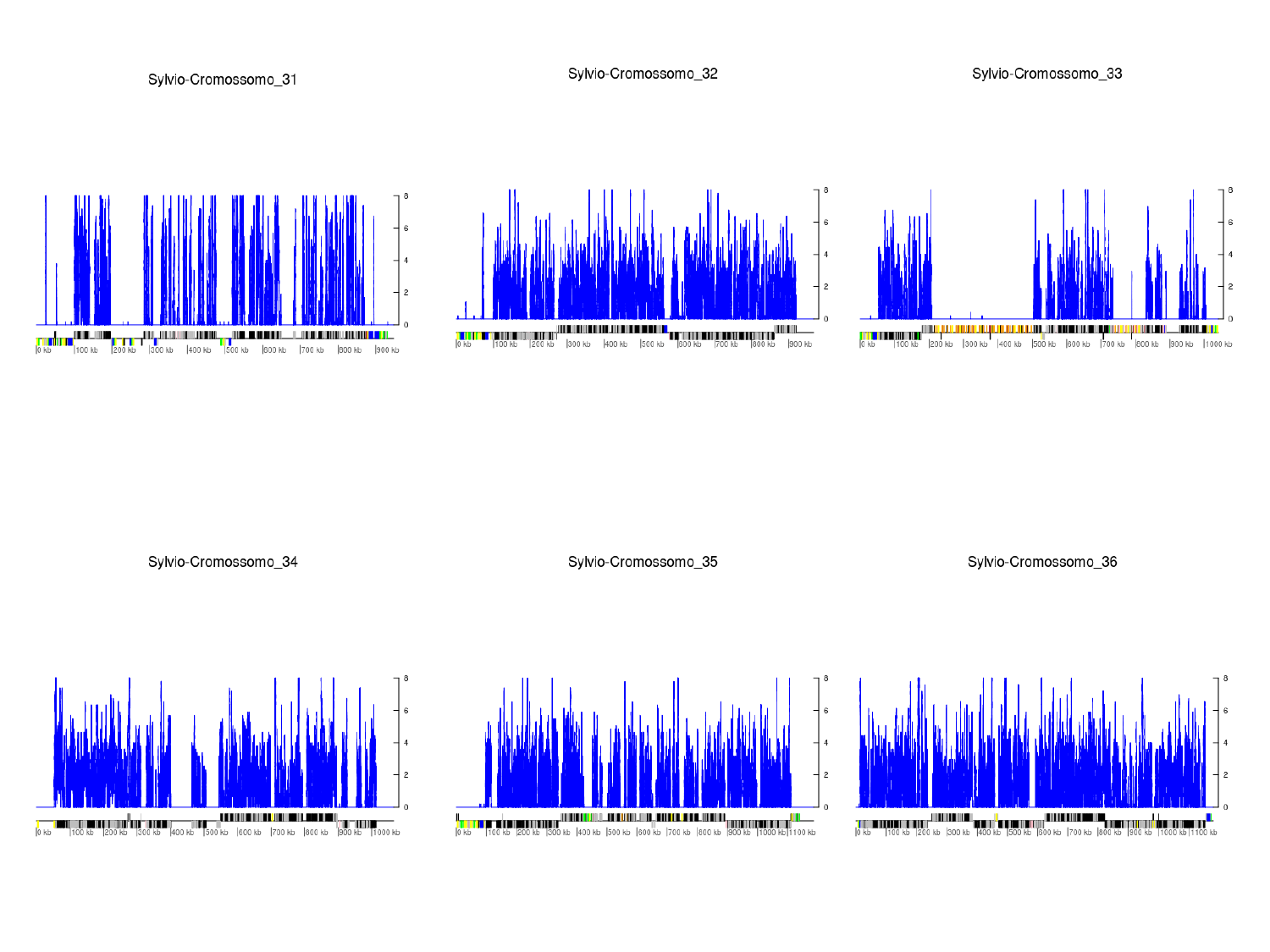

## Slide 25
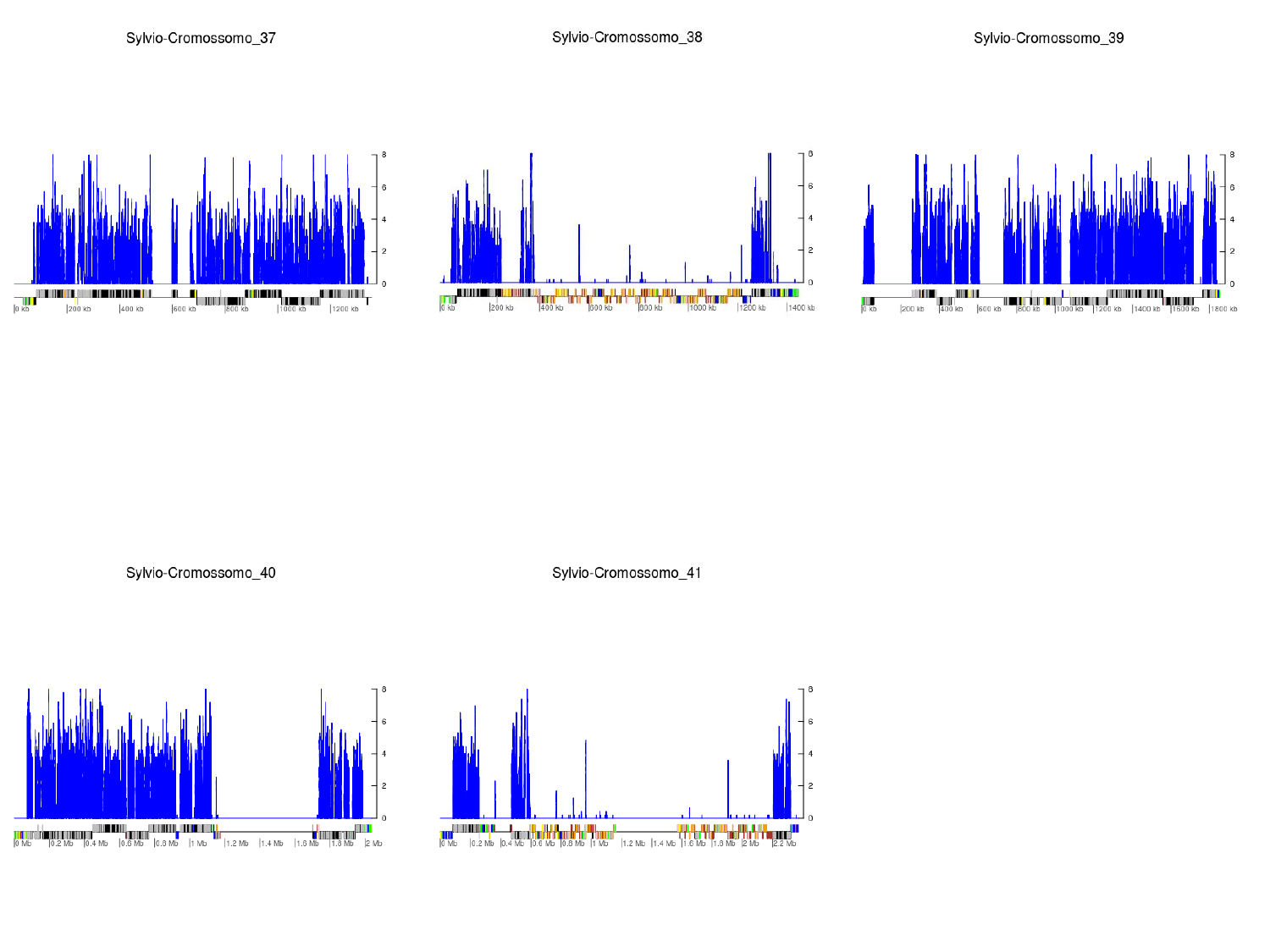

## Slide 26
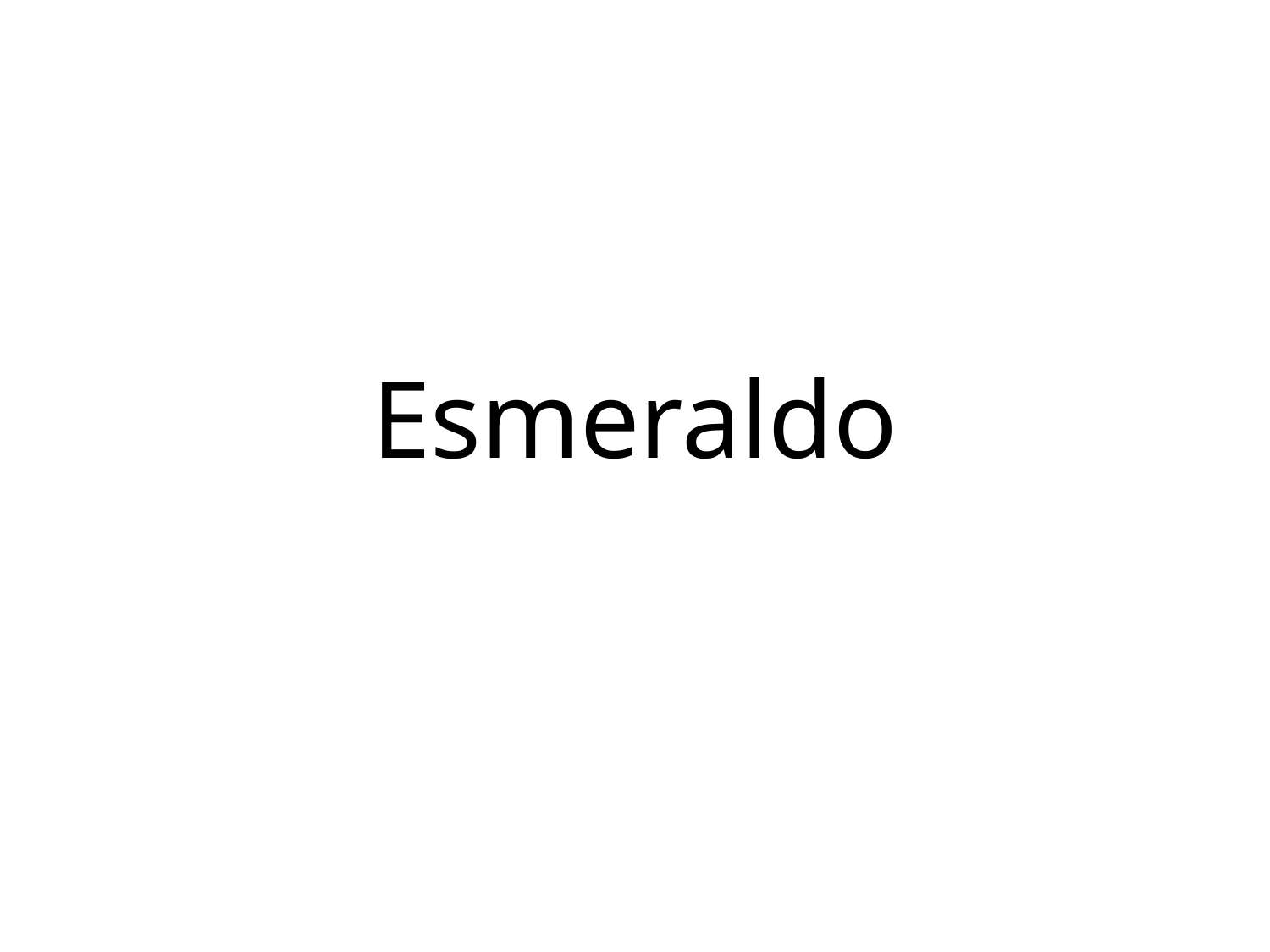

# Esmeraldo

## Slide 27
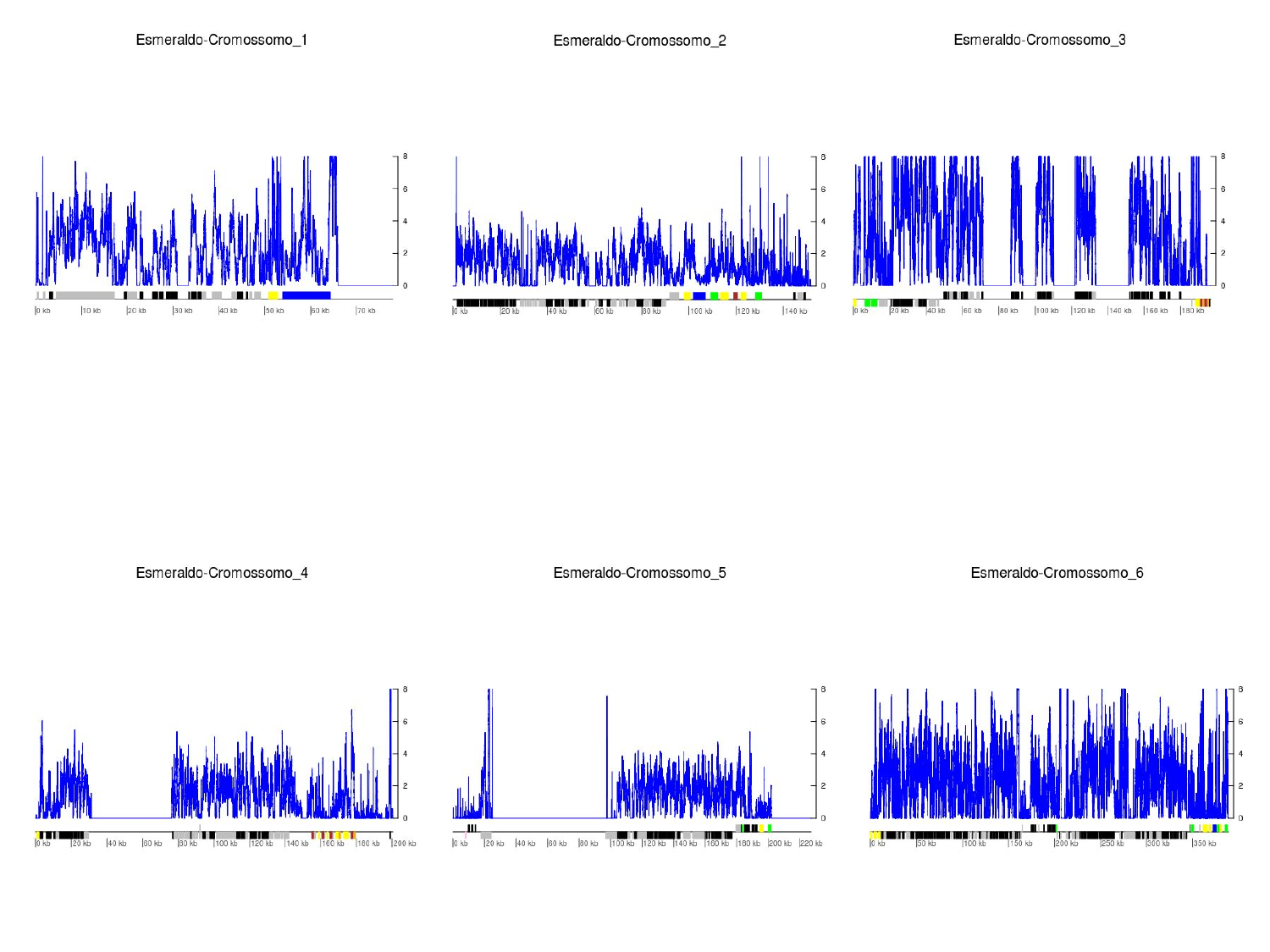

## Slide 28
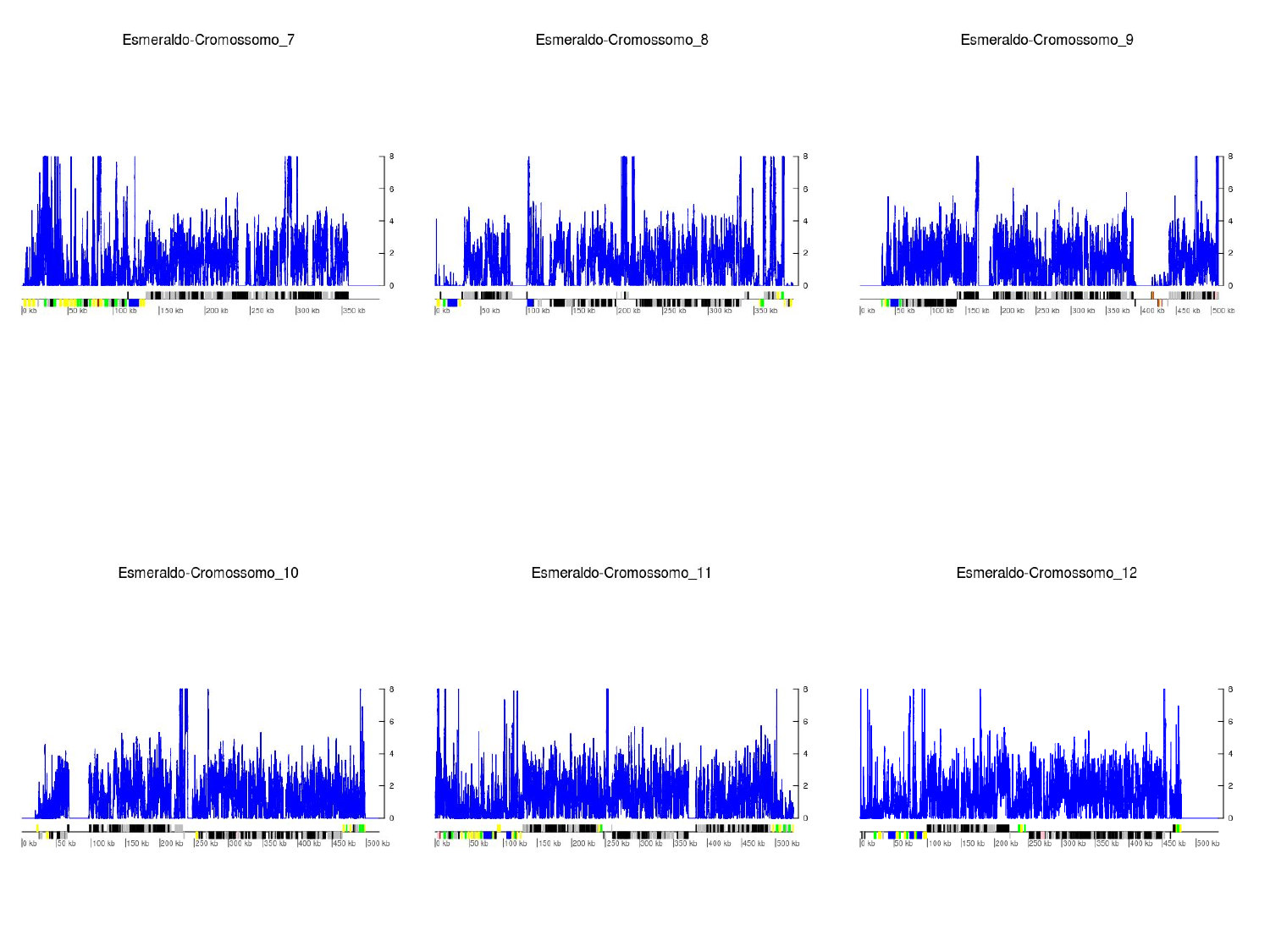

## Slide 29
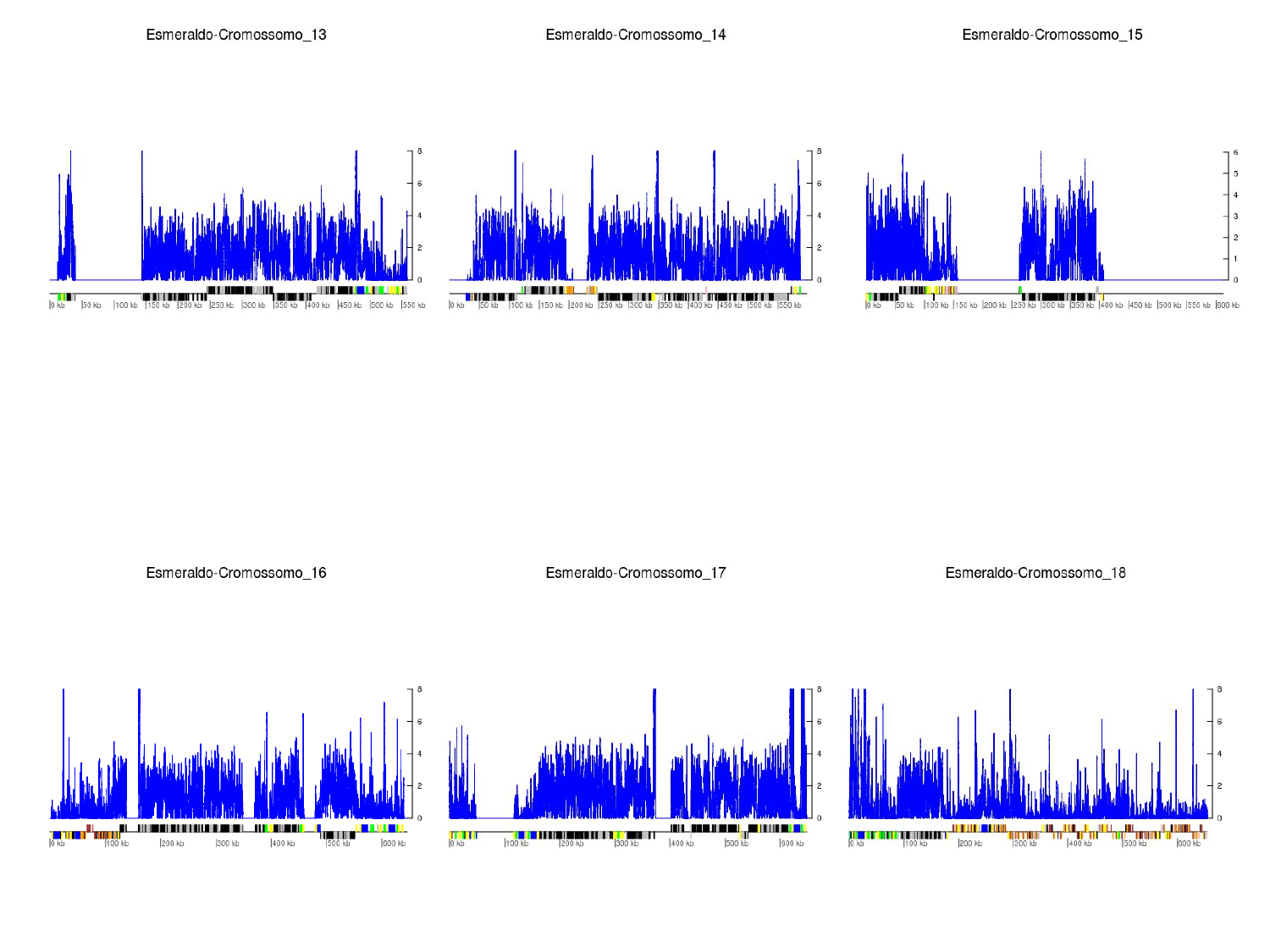

## Slide 30
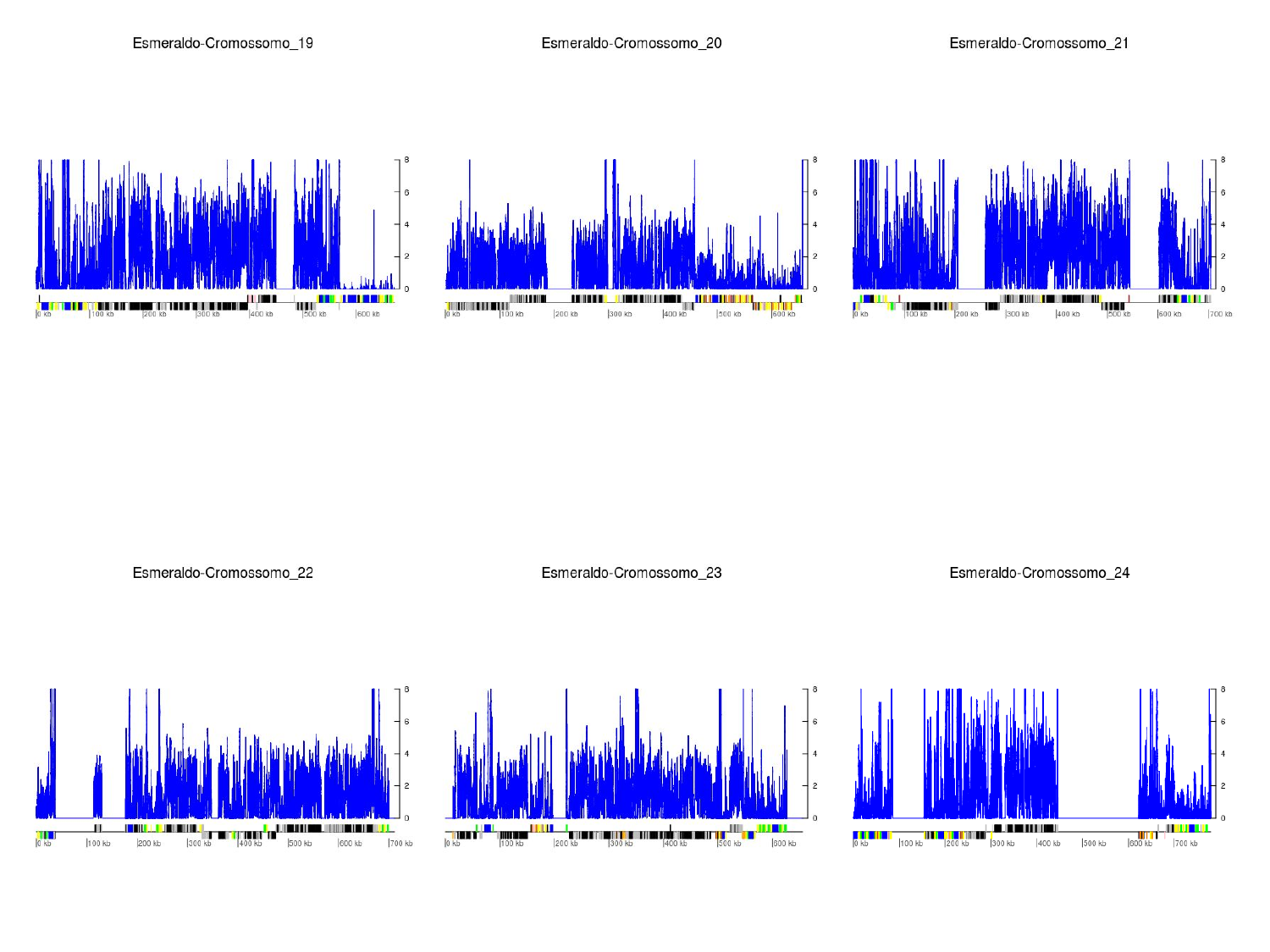

## Slide 31
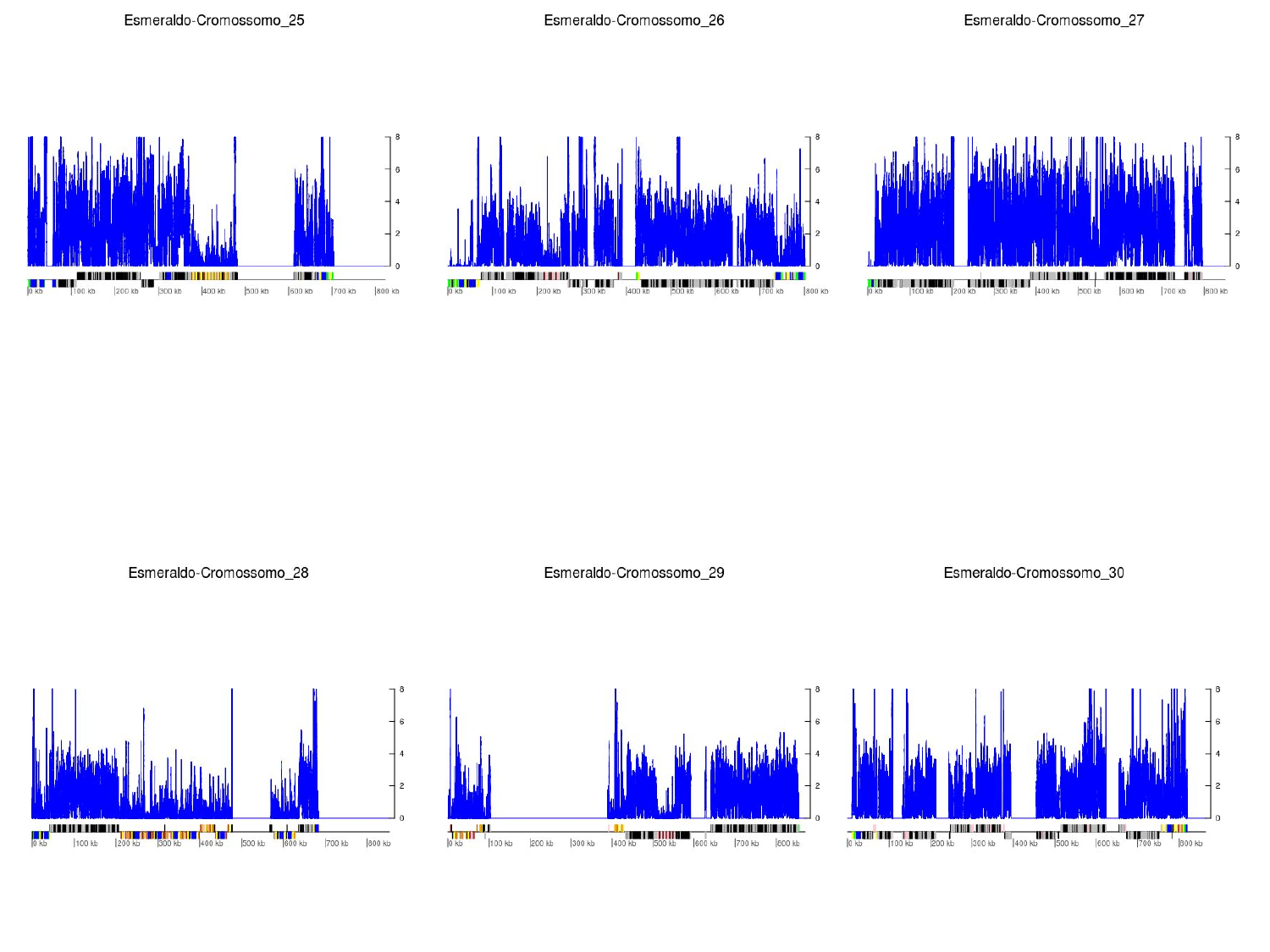

## Slide 32
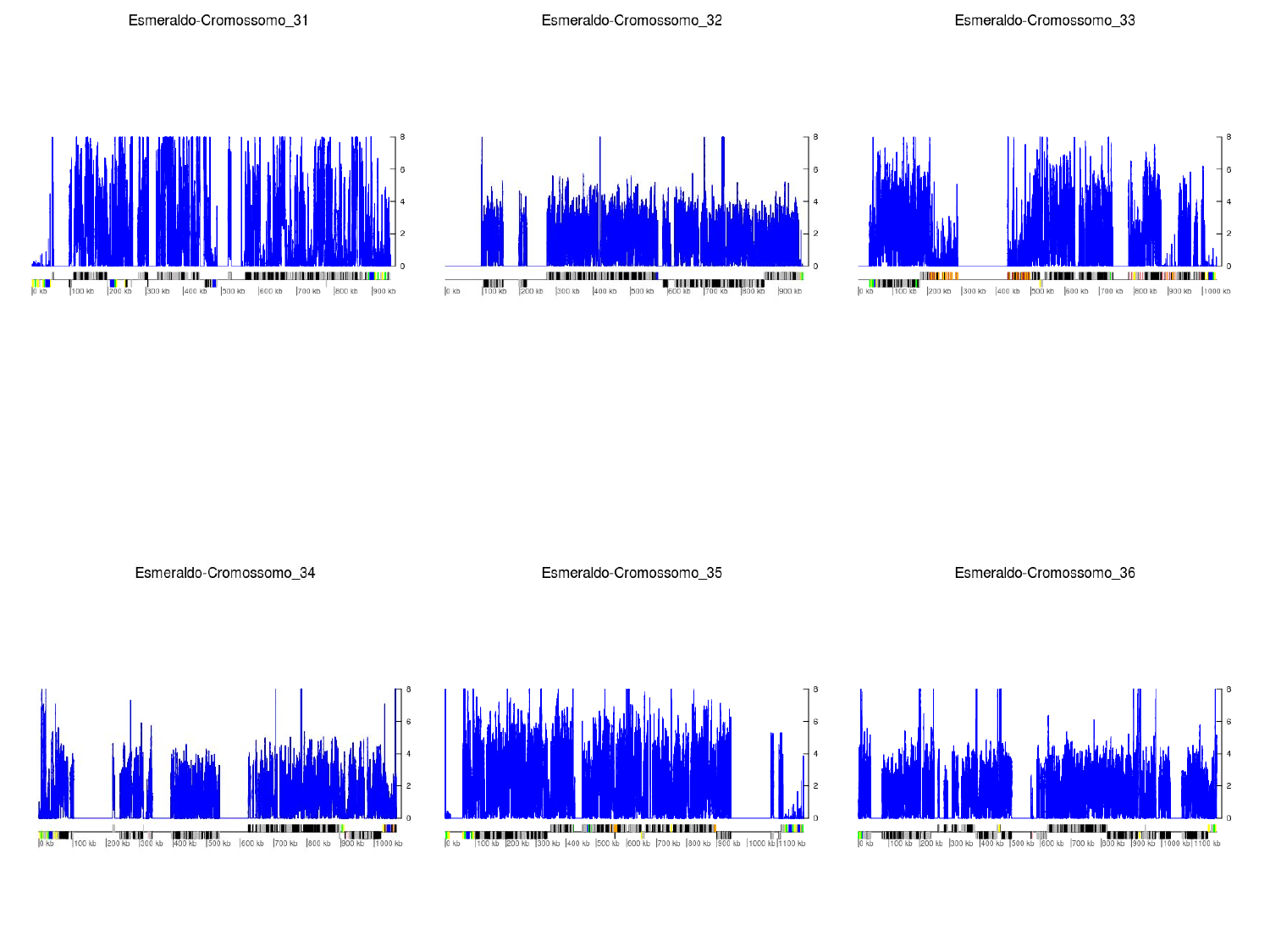

## Slide 33
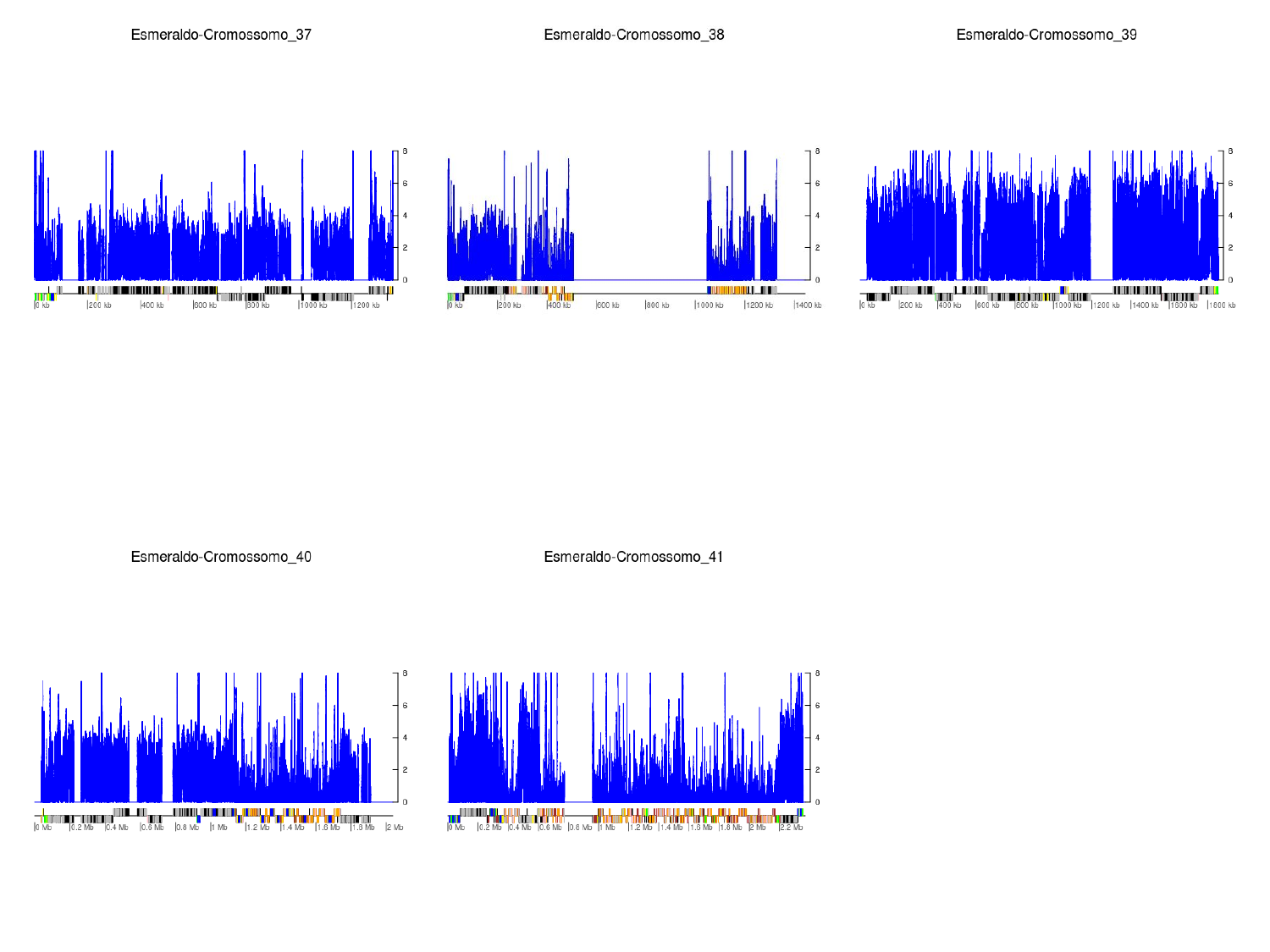

## Slide 34
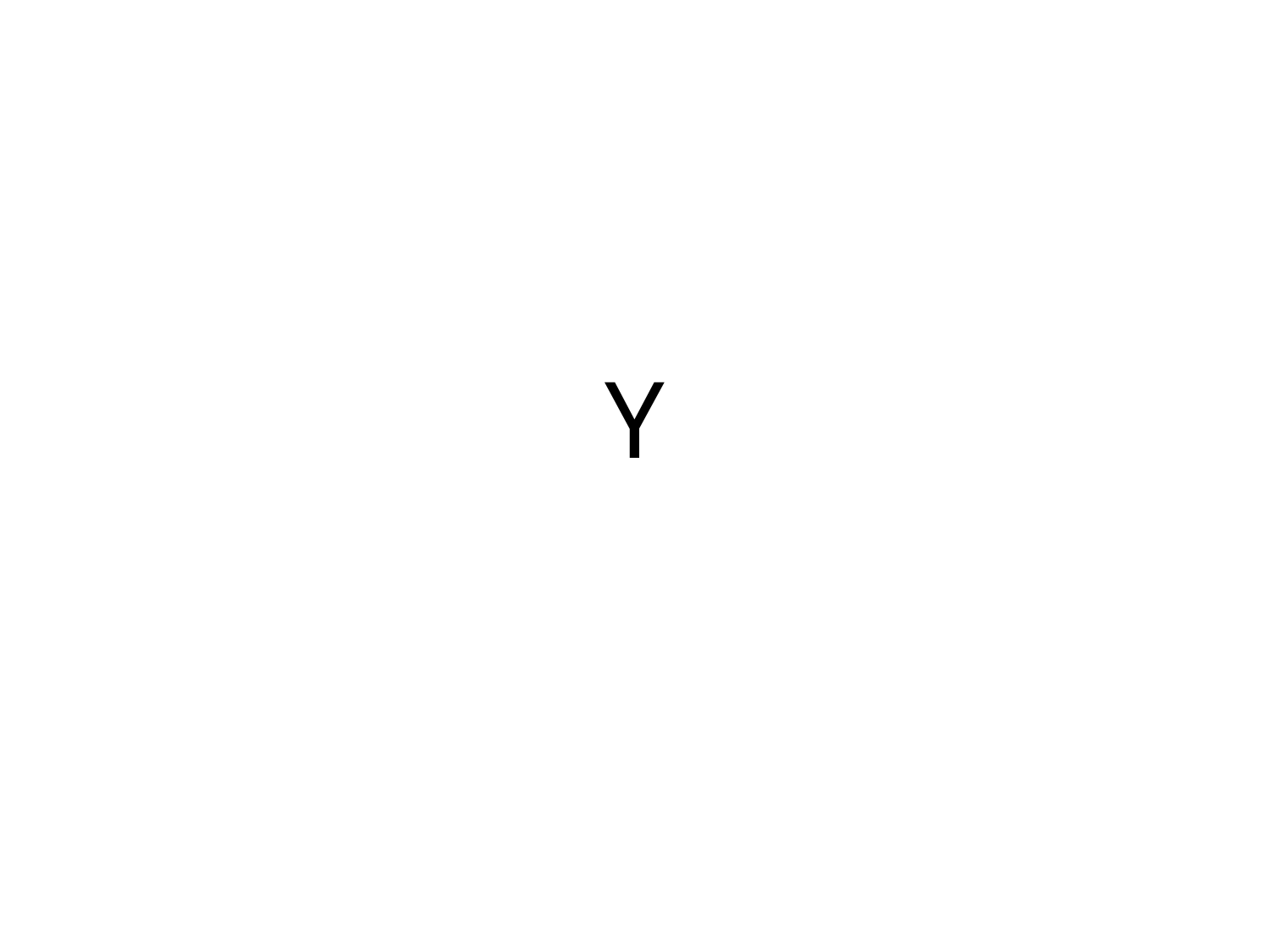

# Y

## Slide 35
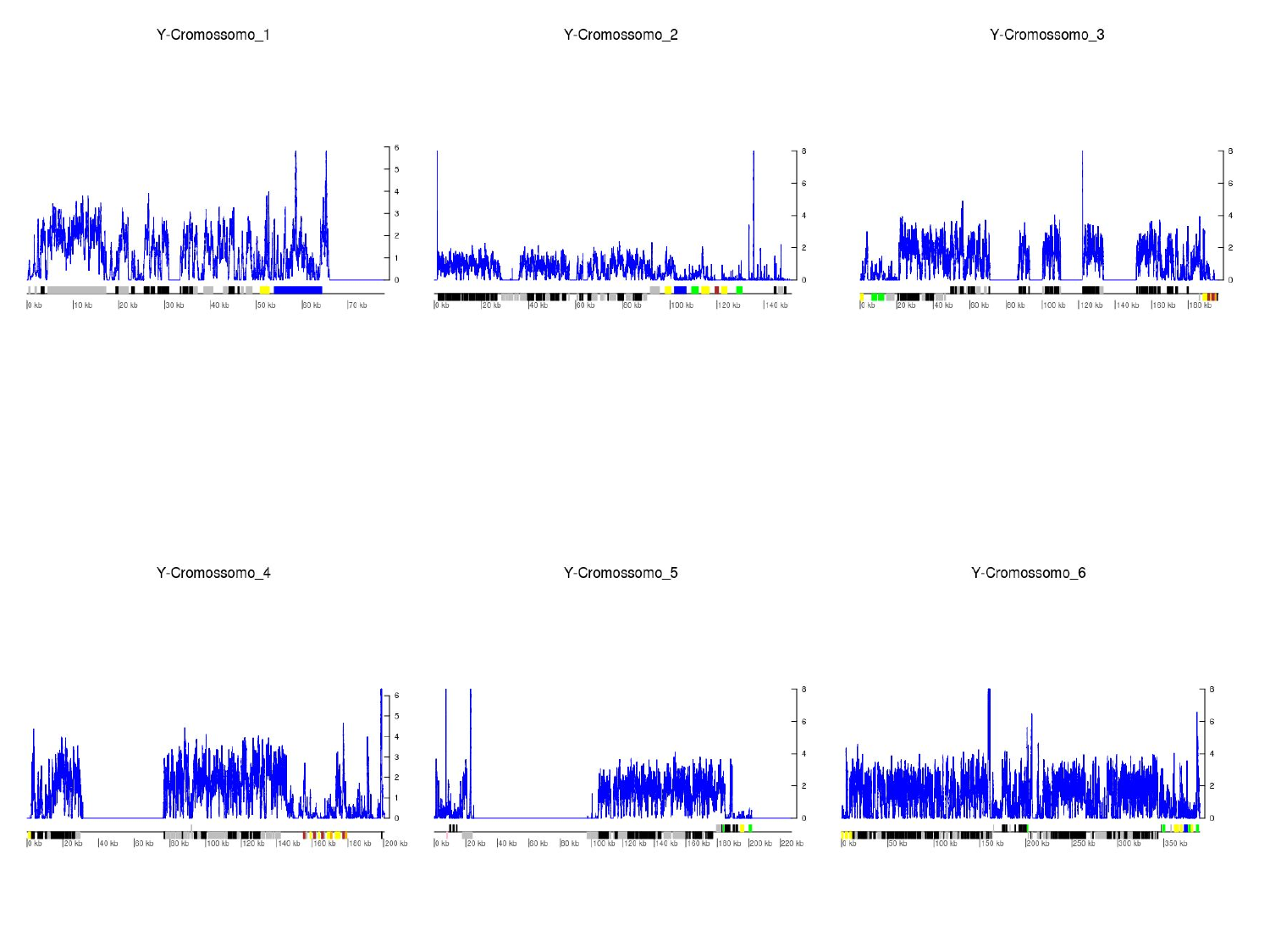

## Slide 36
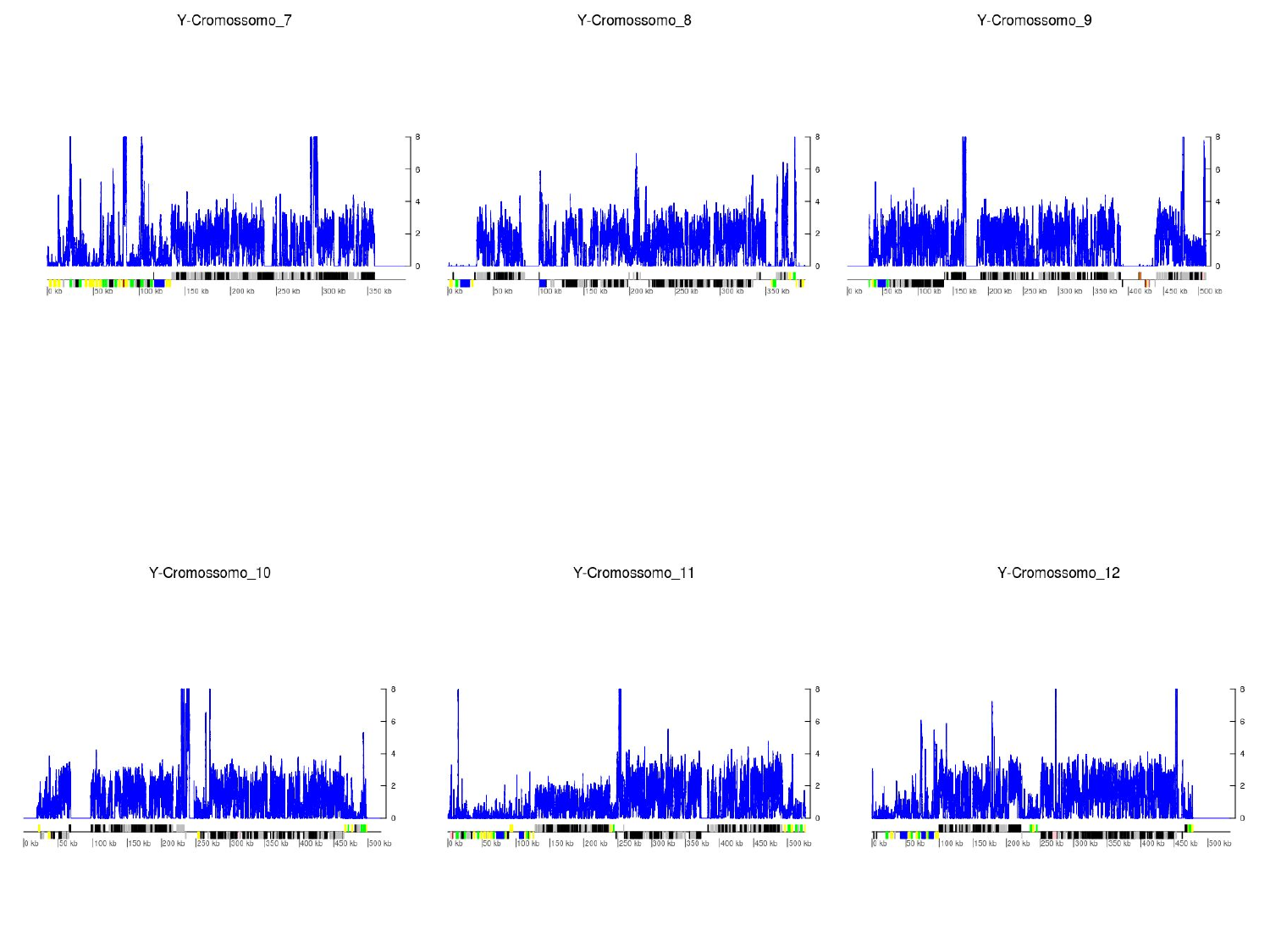

## Slide 37
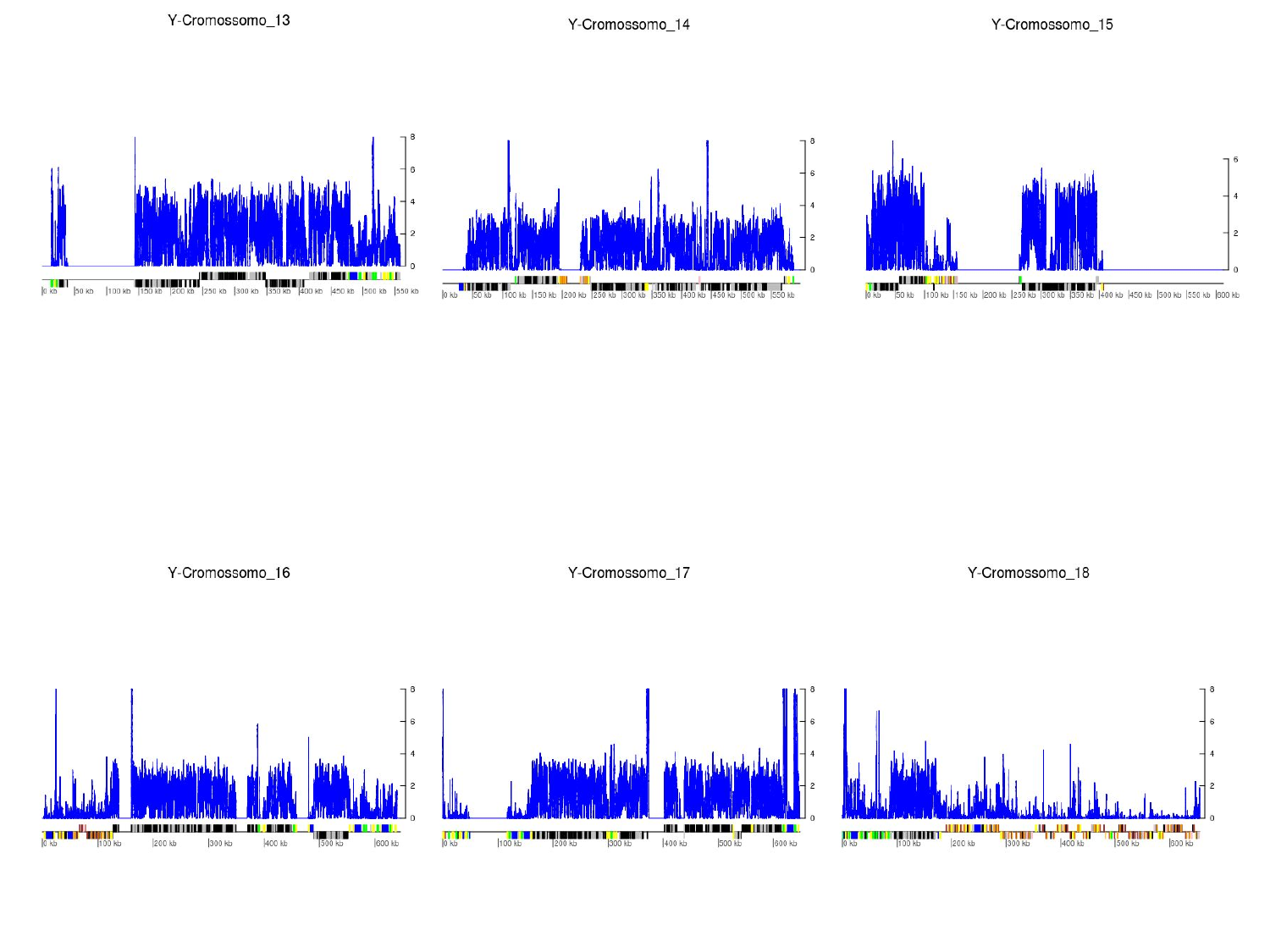

## Slide 38
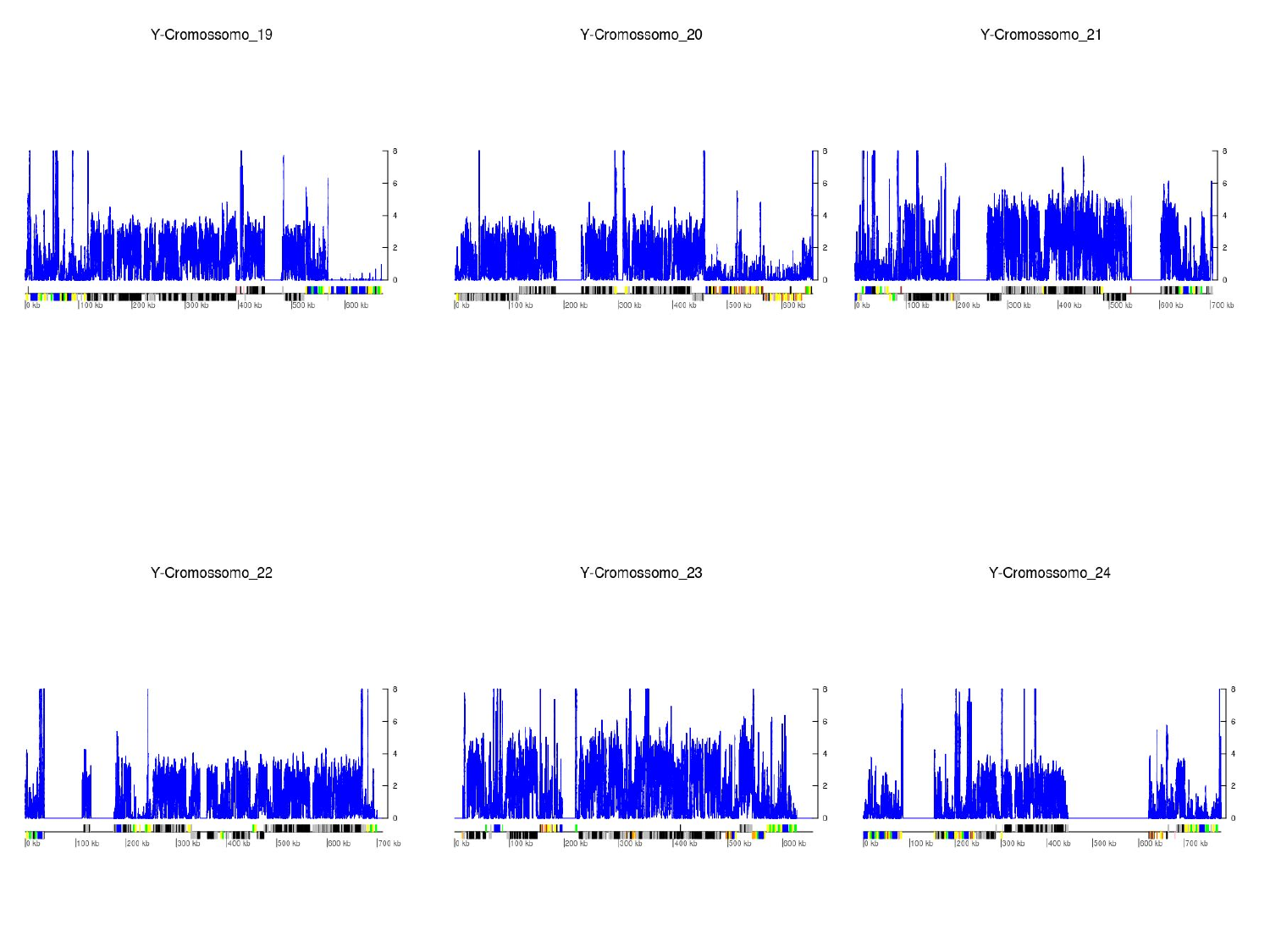

## Slide 39
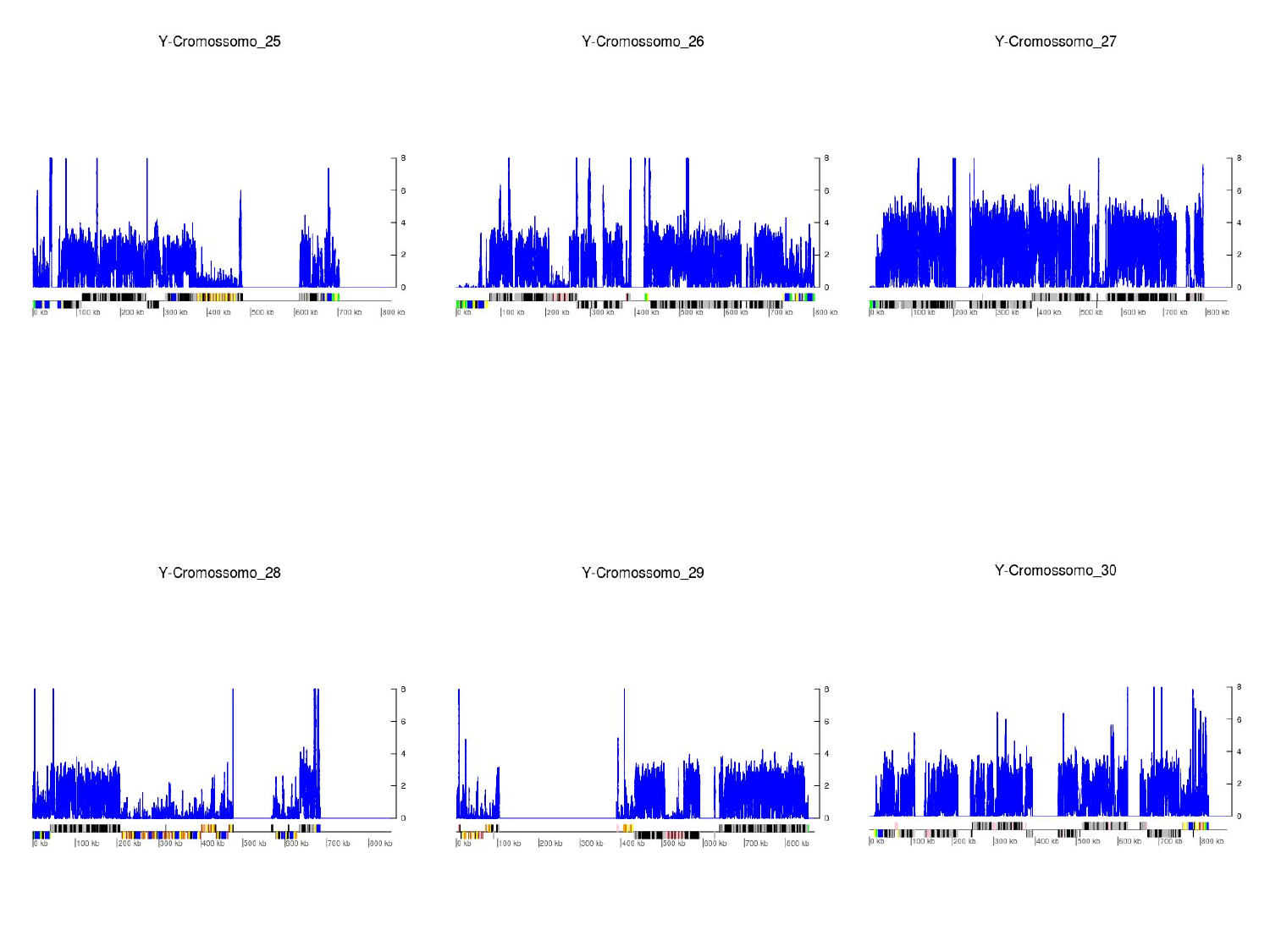

## Slide 40
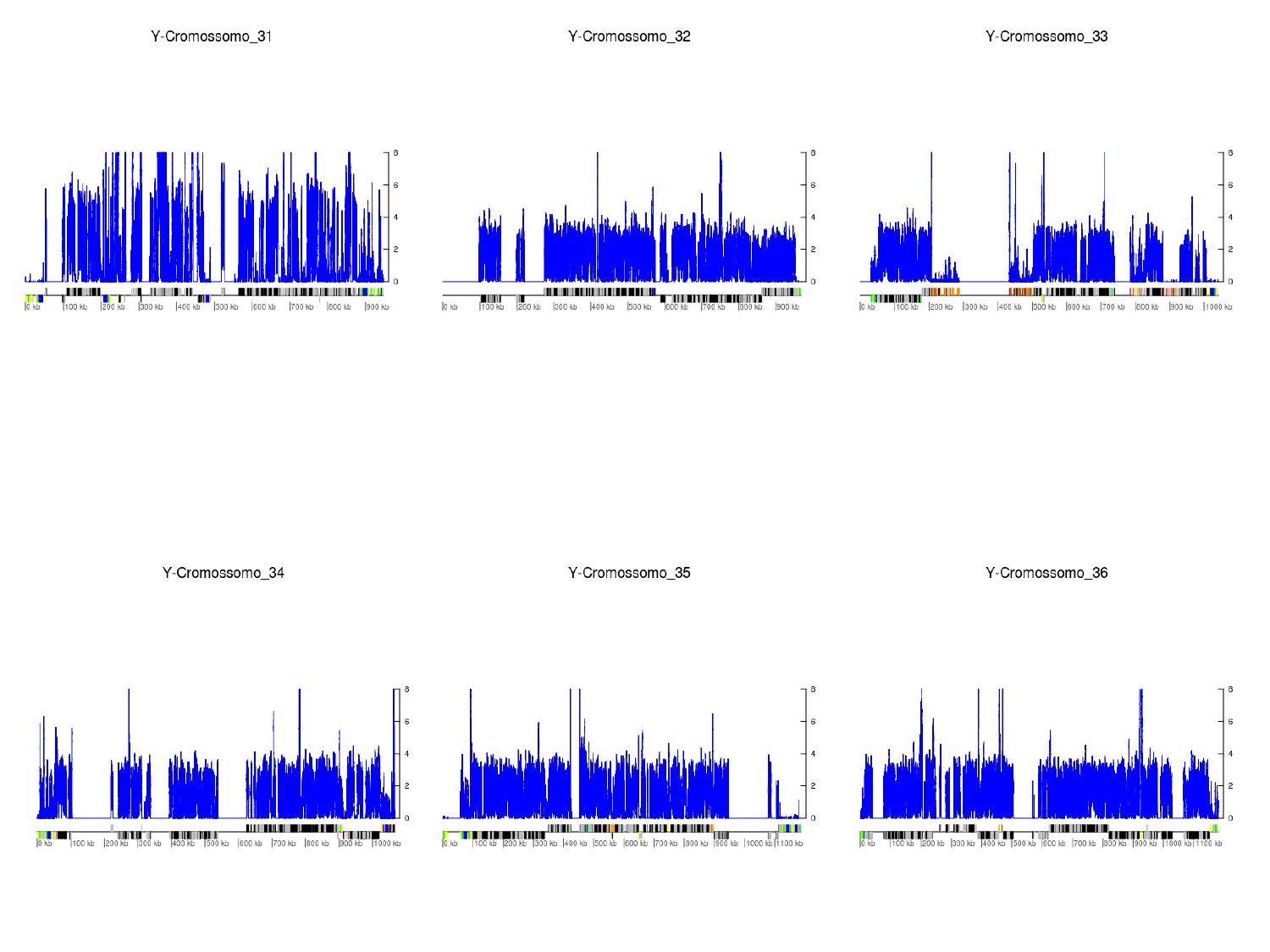

## Slide 41
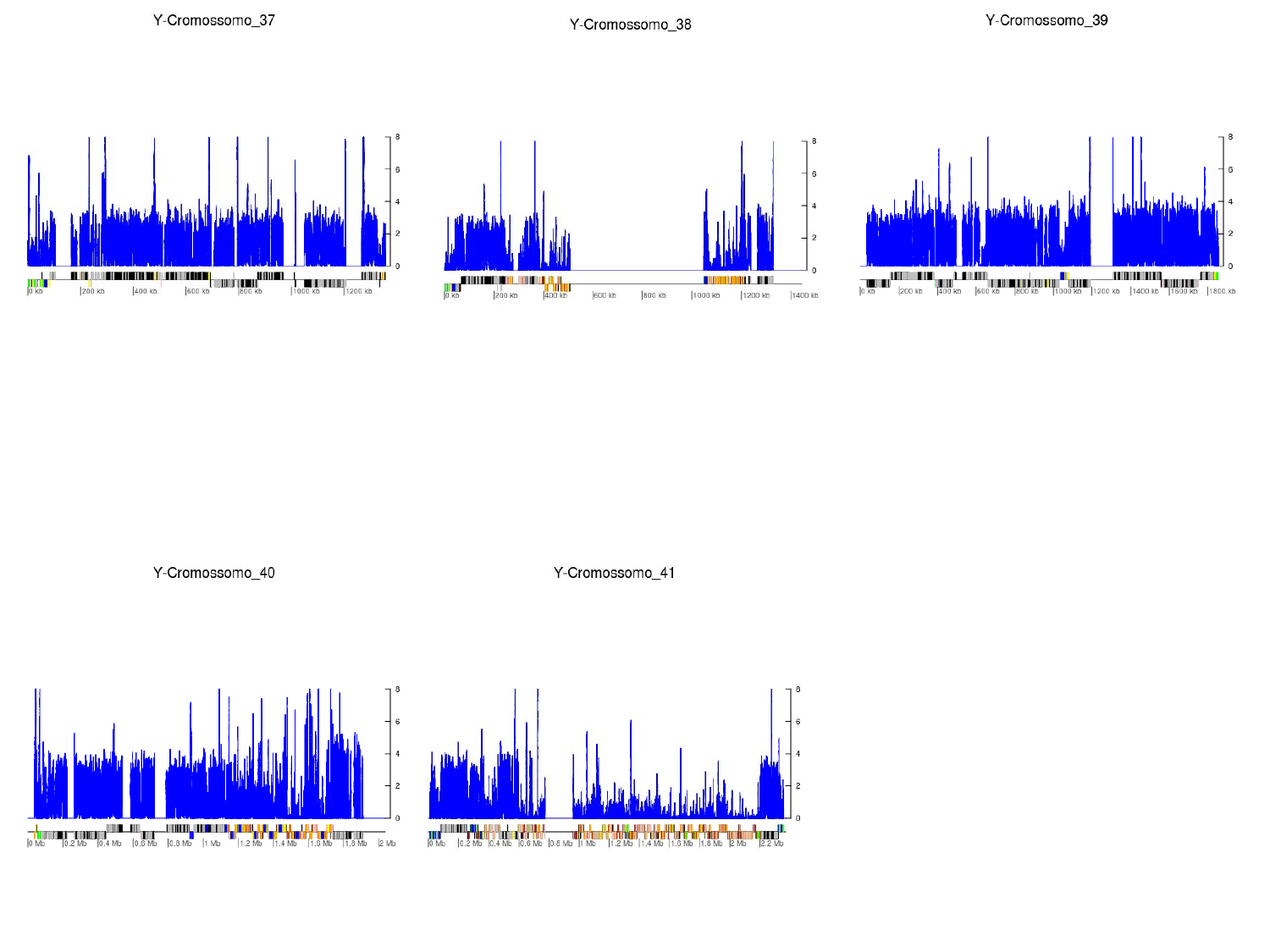

## Slide 42
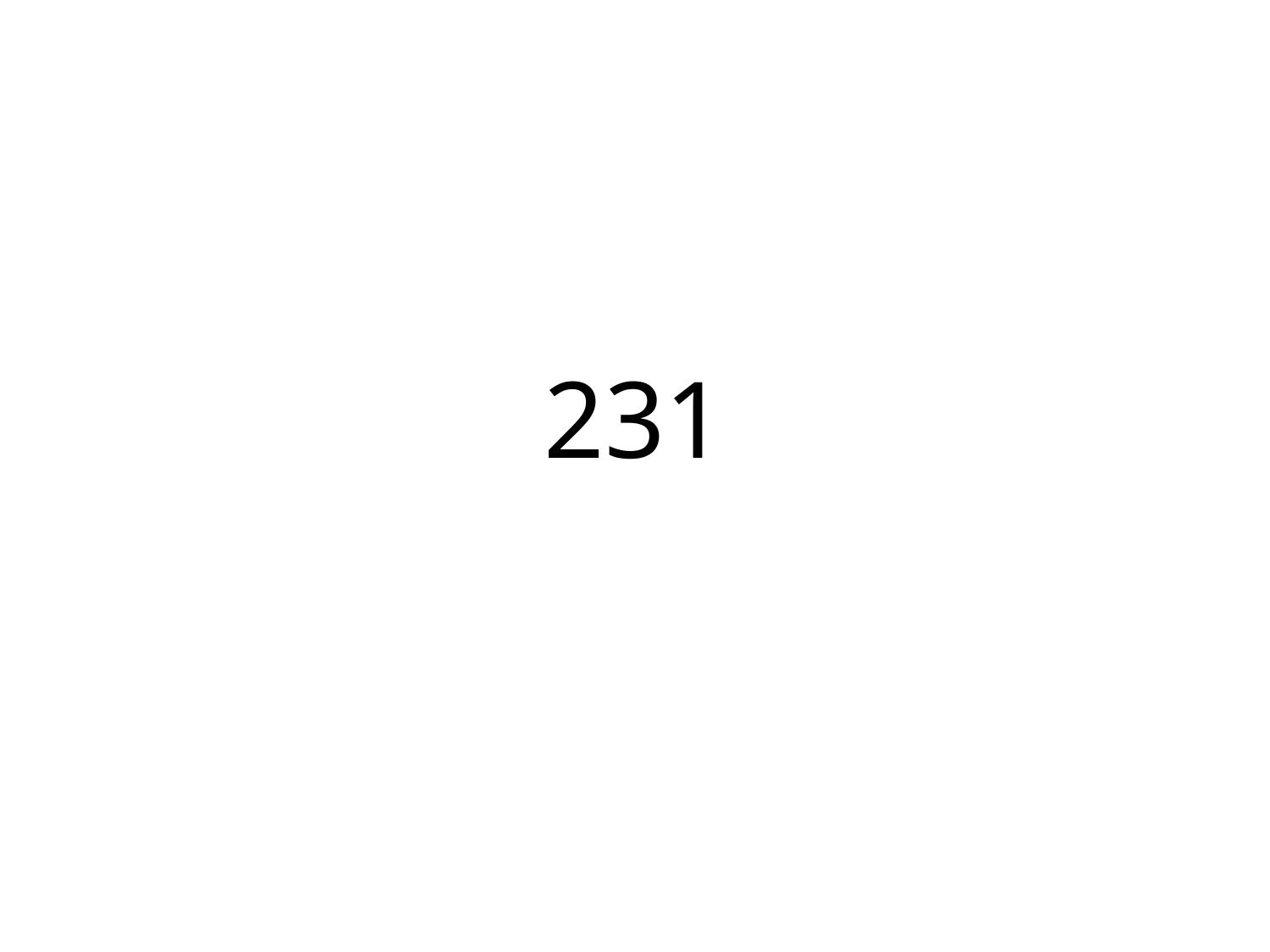

# 231

## Slide 43
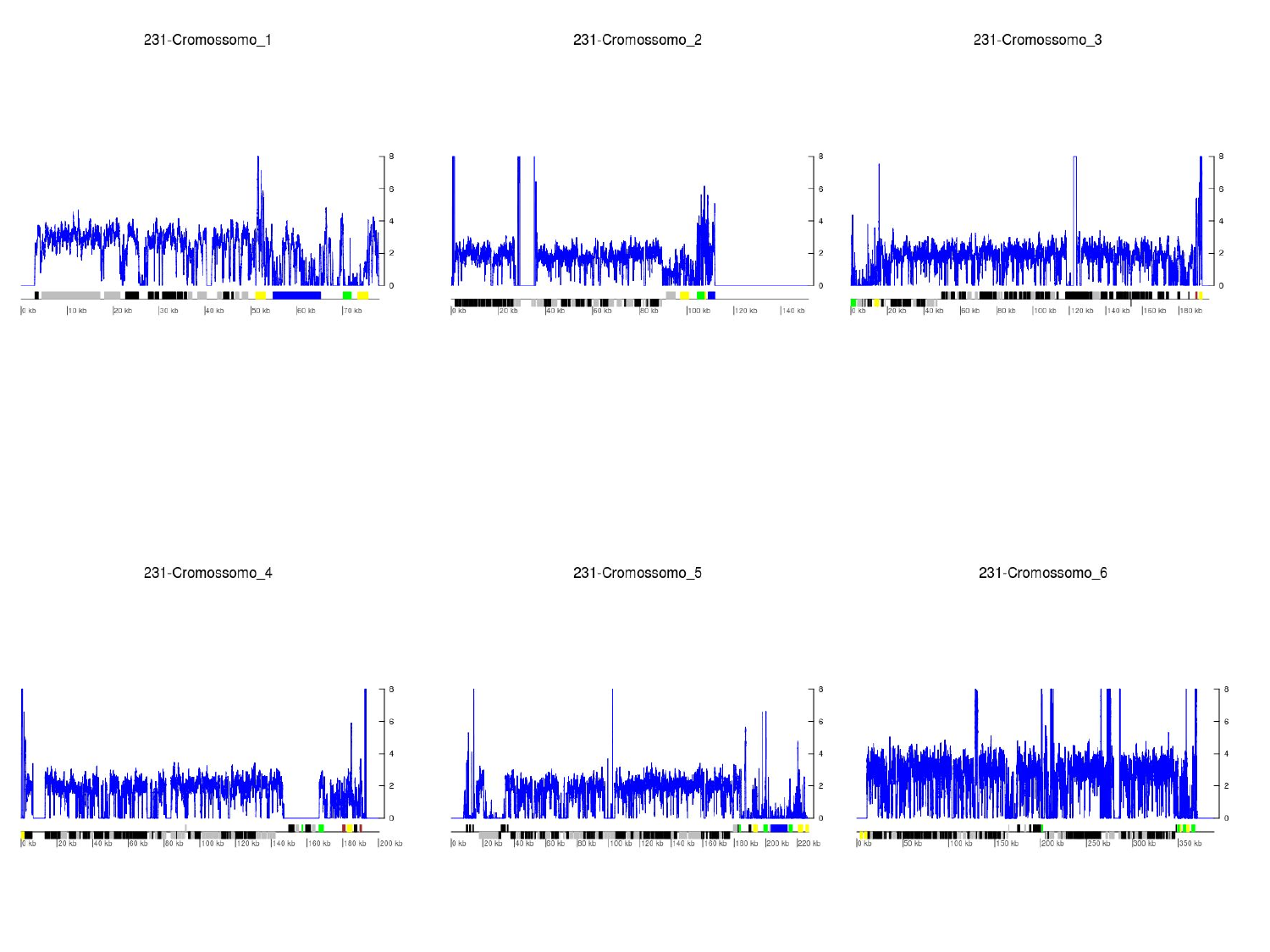

## Slide 44
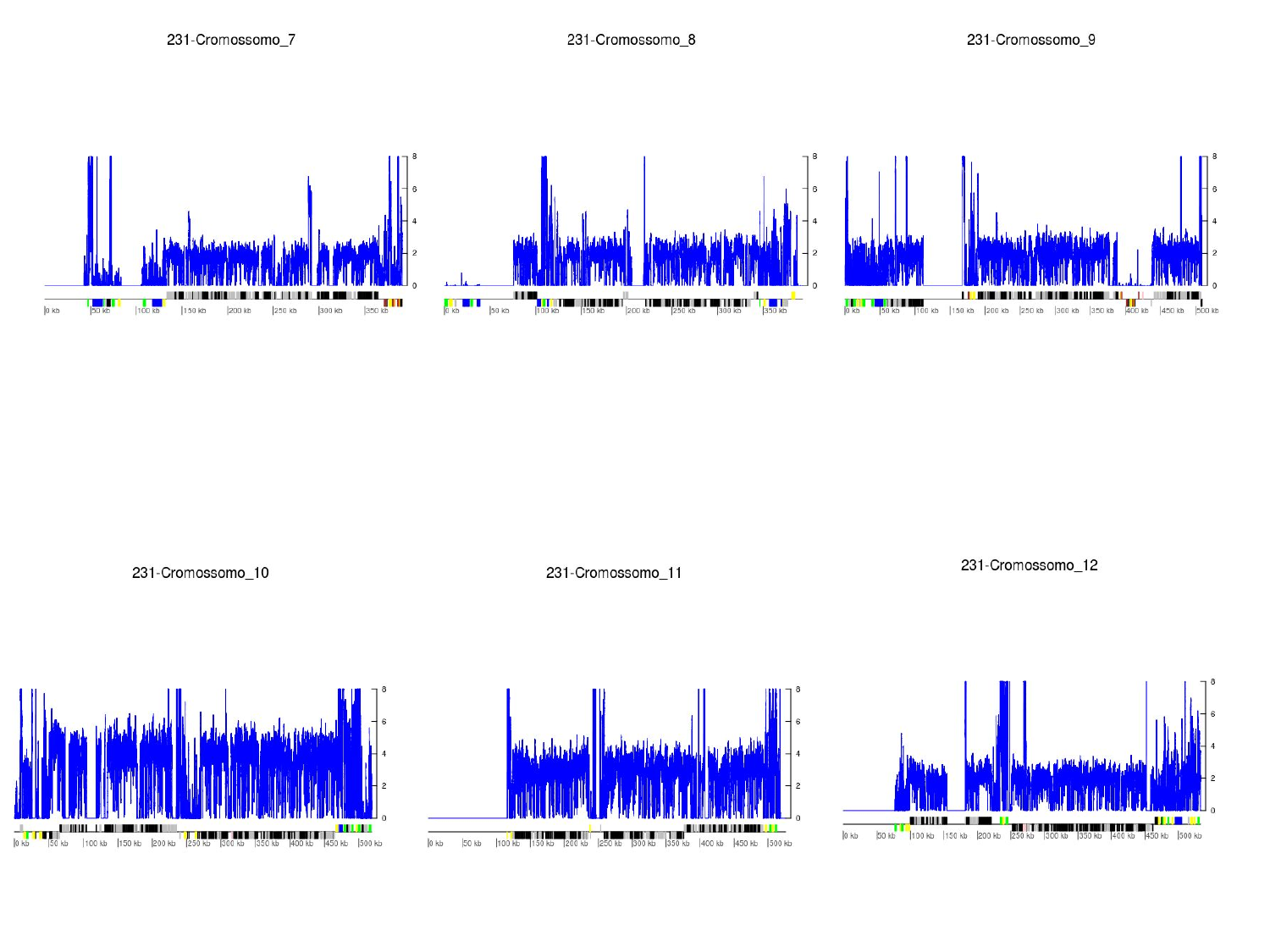

## Slide 45
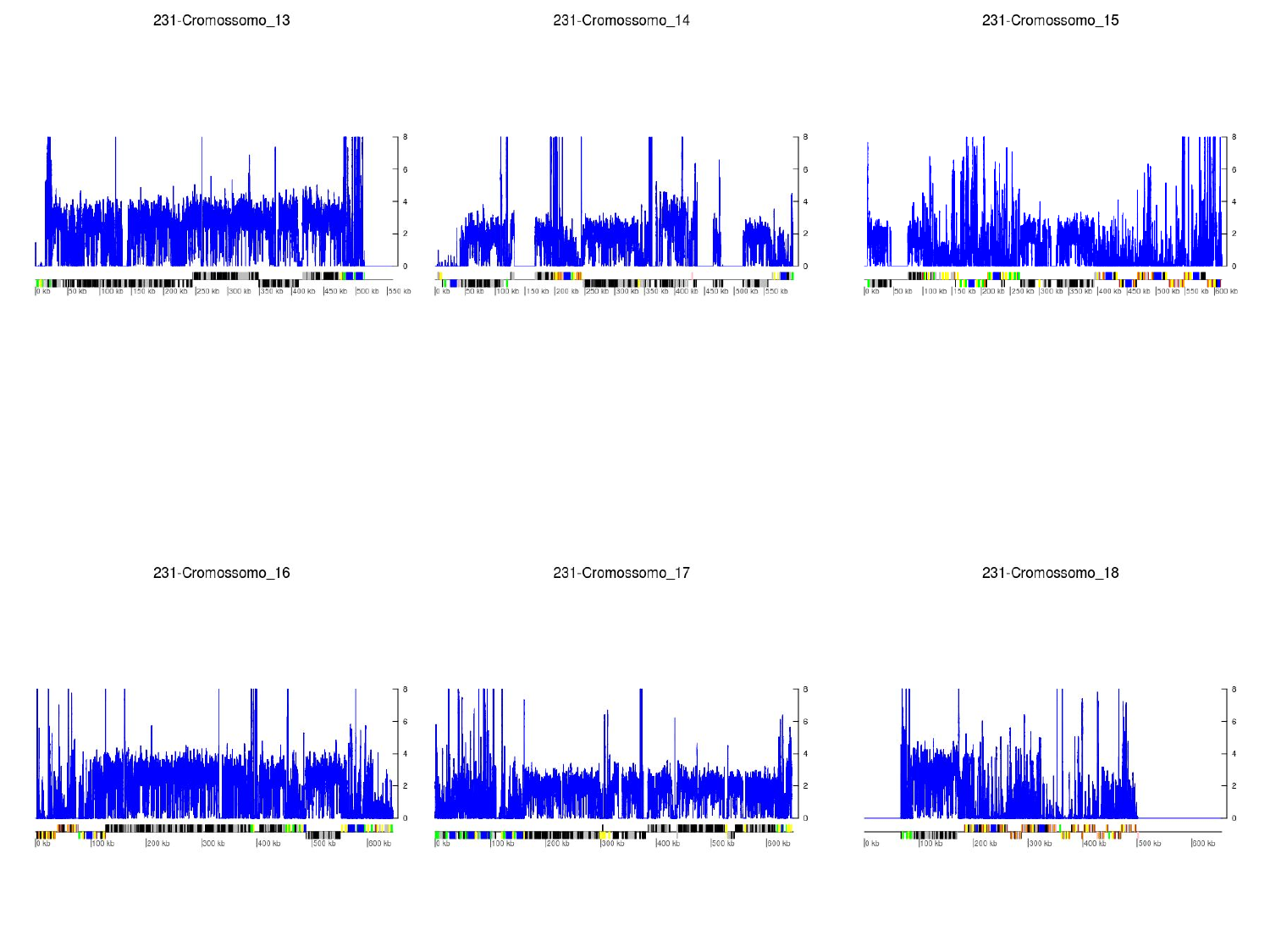

## Slide 46
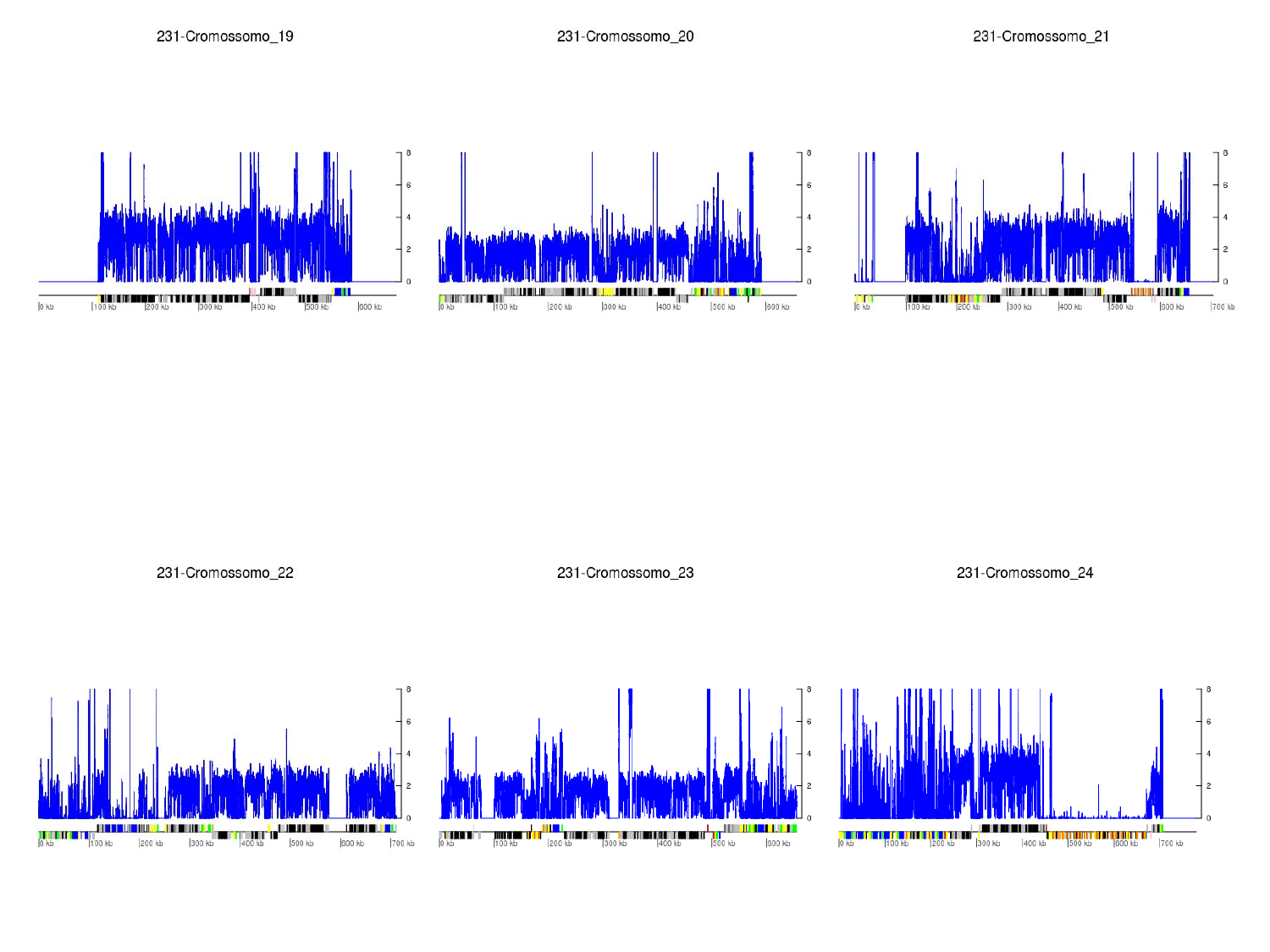

## Slide 47
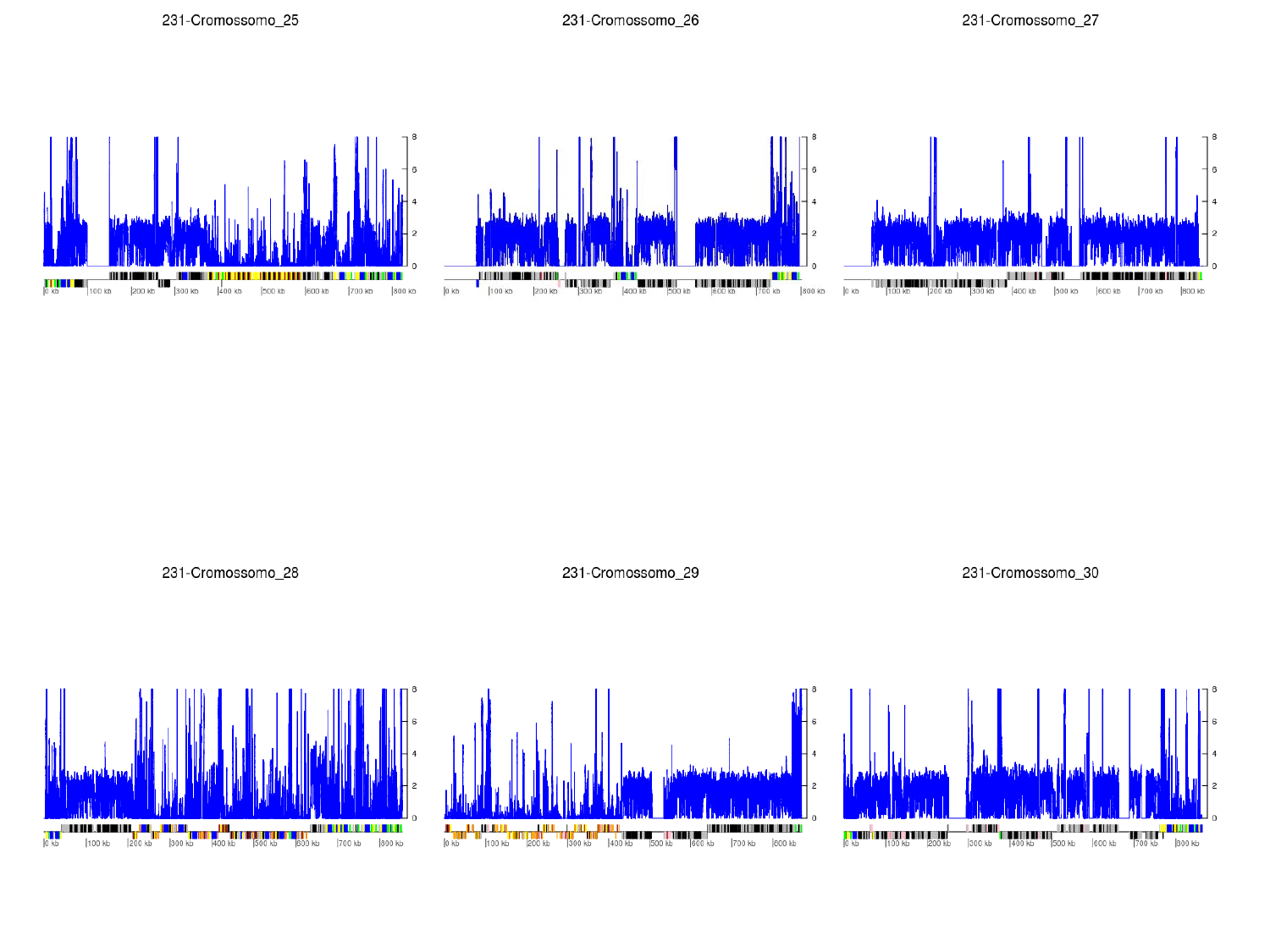

## Slide 48
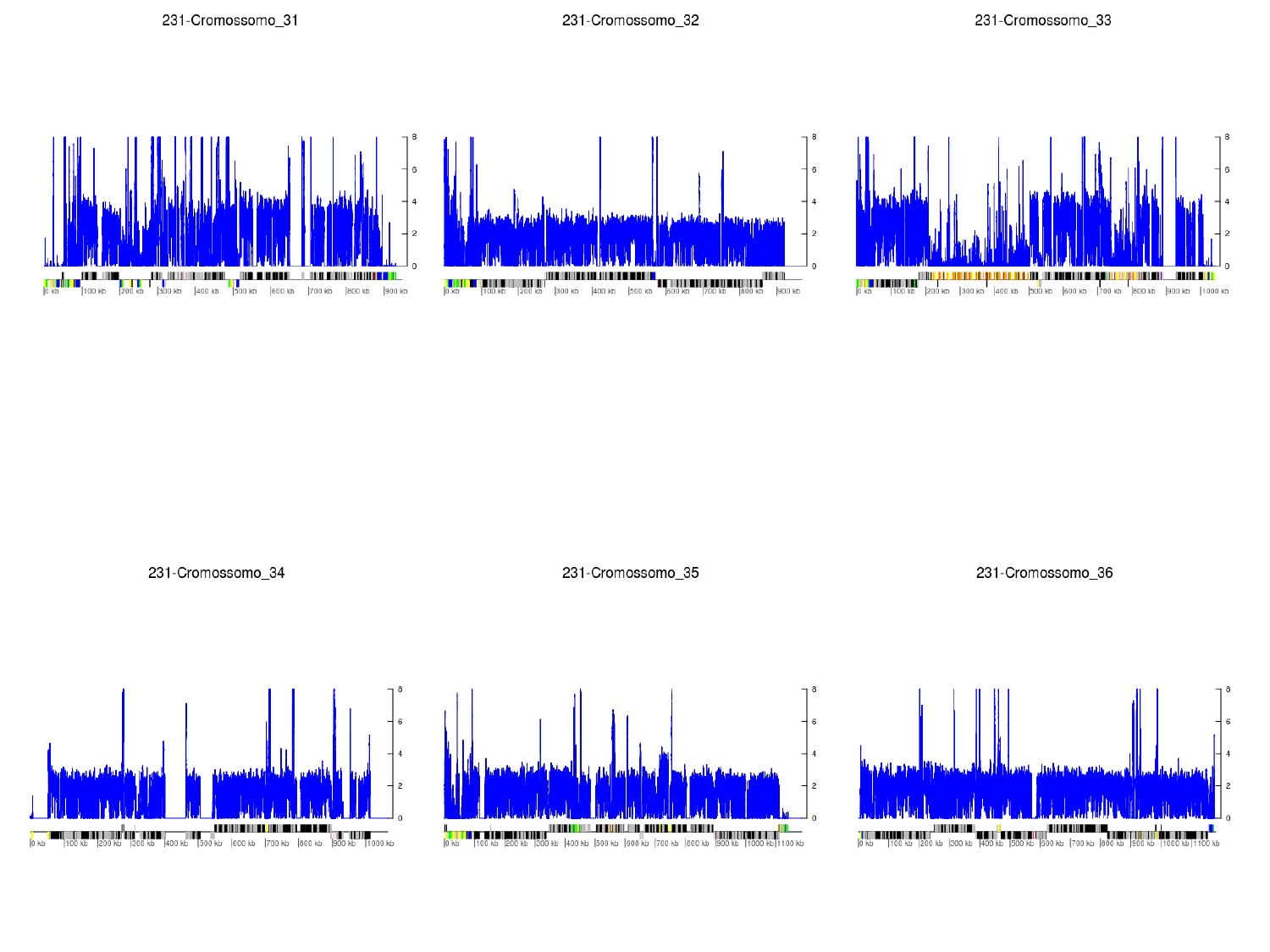

## Slide 49
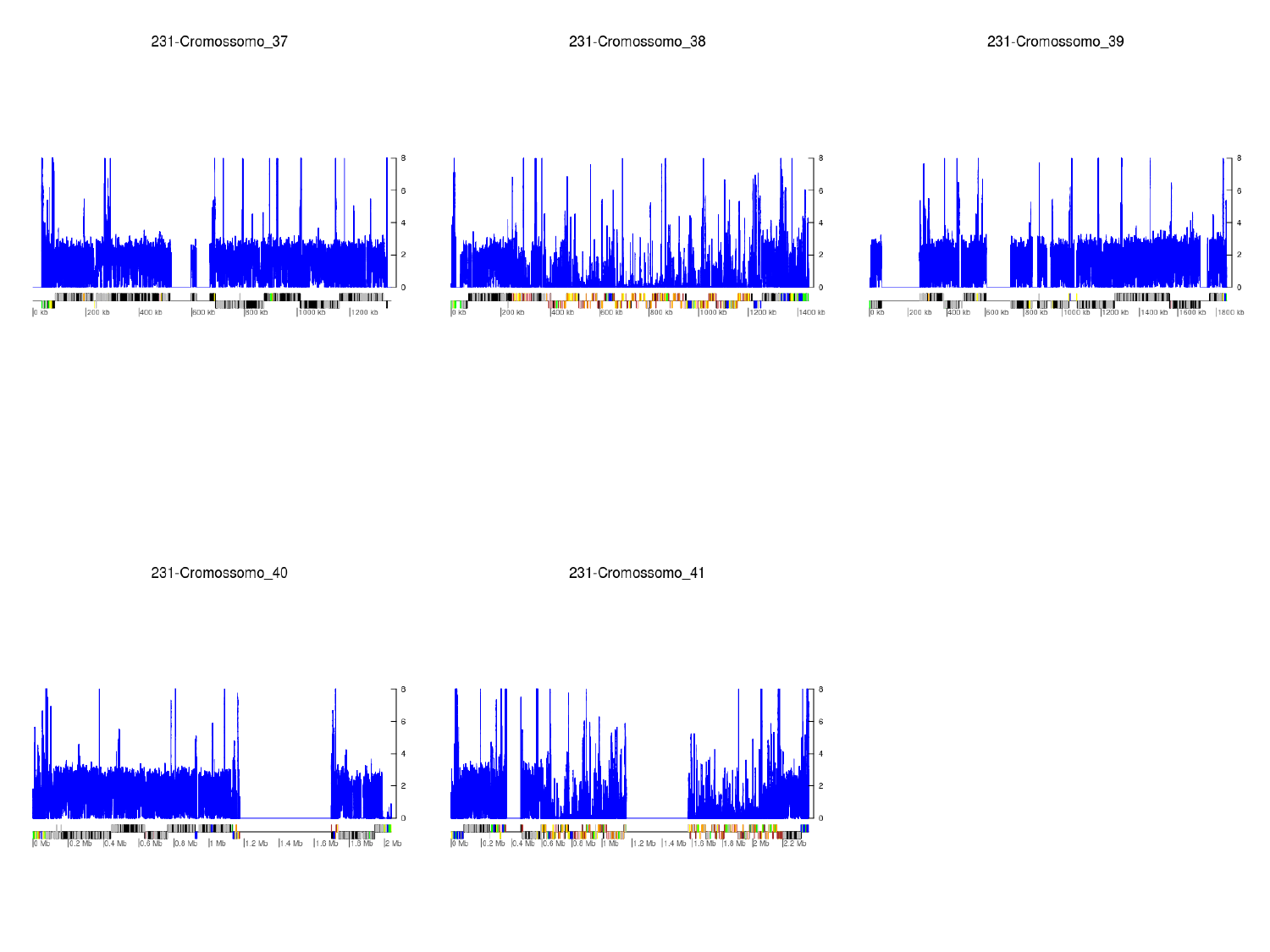

Supplement: Additional file 4: Figure S2. — Normalized RDC of each position on the 41 CL Brener chromosomes by the reads from the T. cruzi strains: Arequipa, Colombiana, Sylvio, Esmeraldo, Y and 231. [file 12864_2015_1680_MOESM4_ESM.pptx]
